# Leak Proof PDBBind: A Reorganized Dataset of Protein-Ligand Complexes for More Generalizable Binding Affinity Prediction

**Published:** 2023-08-18

**Authors:** Jie Li, Xingyi Guan, Oufan Zhang, Kunyang Sun, Yingze Wang, Dorian Bagni, Teresa Head-Gordon

**Affiliations:** † Pitzer Center for Theoretical Chemistry, Department of Chemistry, University of California, Berkeley, CA, USA; ‡ Departments of Bioengineering and Chemical and Biomolecular Engineering, University of California, Berkeley, CA, USA

## Abstract

Many physics-based and machine-learned scoring functions (SFs) used to predict protein-ligand binding free energies have been trained on the PDBBind dataset. However, it is controversial as to whether new SFs are actually improving since the general, refined, and core datasets of PDBBind are cross-contaminated with proteins and ligands with high similarity, and hence they may not perform comparably well in binding prediction of new protein-ligand complexes. In this work we have carefully prepared a cleaned PDBBind data set of non-covalent binders that are split into training, validation, and test datasets to control for data leakage. The resulting leak-proof (LP)-PDBBind data is used to retrain four popular SFs: AutoDock vina, Random Forest (RF)-Score, InteractionGraphNet (IGN), and DeepDTA, to better test their capabilities when applied to new protein-ligand complexes. In particular we have formulated a new independent data set, BDB2020+, by matching high quality binding free energies from BindingDB with co-crystalized ligand-protein complexes from the PDB that have been deposited since 2020. Based on all the benchmark results, the retrained models using LP-PDBBind that rely on 3D information perform consistently among the best, with IGN especially being recommended for scoring and ranking applications for new protein-ligand systems.

## Introduction

Scoring functions (SFs) are crucial in computer aided drug discovery, utilized for selecting the most probable binding pose geometry and/or free energy of binding between a ligand and a protein.^1,2^ There are a plethora of SFs being developed and widely used by computational chemists, but they can be broadly categorized into either physical scoring functions (PSFs),^3–13^ or machine learning scoring functions (MLSFs).^14–20^ PSFs are designed to model intermolecular interactions or missing free energy components, and can benefit both from better design of the functional form, many-body physics, as well as the availability of training data for parameterization of their semi-empirical or empirical functions.^3–9^ Knowledge based SFs by contrast are less reliant on physical interaction modeling and are far more dependent on experimental information,^11,21,22^ culminating in the current development and use of sophisticated machine learning models^14–20^ whose much larger parameter spaces are optimized on large, high quality datasets.

However, it is still an open question as to whether the new MLSFs have truly surpassed traditional PSFs in actual predictive performance, or how well any given PSF or MLSF performs on real-world protein-ligand binding applications.^23–26^ It is already known that any SF model can be overtrained so that it has exceptional performance on the training dataset, a problem which can be mitigated by several known regularization strategies to provide better generalization to an independent test set.^27,28^ Equally important, however, is to control for data leakage into the test set itself, without which can lead to false confidence in predictive capacity when the training dataset has high similarity to the test set, but manifests as poor generalization when the sequence or structure similarity is low.^24^

The majority of protein-ligand interaction SF predictors, whether physical or machine-learned, have been trained on the PDBBind dataset.^29^ The Comparative Assessment of Scoring Functions (CASF) benchmark, which assesses the scoring power, ranking power, docking power and screening power of various SFs, was also conducted on the PDBBind core set.^30^ More specifically PDBBind is a curated set of ~20K protein-ligand complex structures and their experimentally measured binding affinities, in which the ”general” and ”refined” data subsets are used for training, and a separate ”core” set is used for testing. However, not all protein-ligand complex structures in the general and refined data sets are as high-quality as those in the core set which contains protein-ligand complexes with the best structural resolution and most reliable binding affinity data.^29^ Additionally, the average size of the ligands in the core set is also smaller than that in the rest of the PDBBind dataset, which may indicate that the core set are easier prediction targets.^31^

To make more fair comparisons, Stärk, et al. suggested splitting PDBBind according to a time cutoff in the creation of the EquiBind dataset.^31^ This idea mimics a ”blind test” setting, by which the model can only be trained with data released in year 2019 or earlier, and predictions are made with data after year 2019, so that the test data will never be covered by training data. This time-based data splitting marks a significant step forward in reporting more realistic evaluation performance of the tested SFs. However as we show below, the majority of the core data records in the PDBBind dataset have identical proteins and/or ligands with that found in the general and/or refined sets. As such, most empirical PSF and MLSF models have been trained with significant data leakage, and thus their reported performance on the core set is only a true measure of new protein-ligand complexes with high similarity, but will inevitably have limited transferability to low-similarity ligand-proteins scenarios. Hence a purely time based splitting will not solve the problem of data leakage. Specifically, since new drugs are being developed that can interact with popular protein targets that have been established for years,^32,33^ and existing drug molecules may also be tested on new proteins,^34^ it is rather frequent that we see almost identical proteins or ligands in the latest experiments with earlier assays. Under this circumstance, a time based splitting of the dataset is still not an ideal solution.

In this paper, we aim to reorganize PDBBind data into a new set of train, validation and test datasets which we call Leak Proof PDBBind (LP-PDBBind), by incorporating a carefully designed algorithm to control the data similarity between the train, validation and test dataset. Furthermore we also carefully clean the PDBBind data to eliminate covalent bound ligand-protein complexes (thus focusing on non-covalent binding), certain ligands with very low frequency occurrences of certain atomic elements, to remove obvious steric clashes, as well as maintaining consistency in reported binding free energies and their units. We then use the cleaned LP-PDBBind data for the development of new versions of several popular SFs, including AutoDock vina,^3^ RF-Score,^14^ IGN,^15^ and DeepDTA.^18^ Furthermore, in order to provide a true independent benchmark for the new SFs that result from retraining using the cleaned LP-PDBBind, we created a new evaluation dataset, BDB2020+. BDB2020+ is compiled based on data records in the BindingDB dataset^35^ that were deposited after year 2020, and further filtered according to the same similarity control criteria used for the development of the new LP-PDBBind. As a further test of ranking power, we additionally prepared two sets of experimental binding affinity data for different ligand complexes of the SARS-CoV-2 main protease (Mpro)^36^ and epidermal grow factor receptor (EGFR),^37^ neither of which was included in the PDBBind dataset (although Mpro has similar SARS-CoV-1 protease proteins in PDBBind).

The results show that 3-dimensional structure based SFs like AutoDock vina, IGN and RF-Score are able to improve significantly when the model is trained using the new training set provided by LP-PDBBind, while purely data-driven approaches that only use 1-dimensional string representations for the proteins and ligands, such as DeepDTA, perform worse, indicating their reduced capability to generalize. Given that the same model architecture or functional form can still achieve enhanced performance with our new splitting of PDBBind, which contained even less training data than what these models originally used, we believe the cleaned and resplit LP-PDBBind data provides a better way of utilizing the existing PDBBind dataset, and provides a more realistic and meaningful benchmark to help develop higher quality and more generalizable SFs for real-world applications. All data and analysis scripts are available in our lab’s github repository (https://github.com/THGLab/LP-PDBBind/tree/master).

## Method

### Description for processing the PDBBind dataset

The PDBBind v2020 dataset includes 14108 protein-ligand complexes in the general set, 5050 complexes in the refined set and 285 complexes in the core set.^30^ All PDB files were downloaded from RCSB to recover the original headers, and the categories of the proteins were defined according to whether the keywords occurred in the header. The categories we considered include transport proteins, hydrolases, transferases, transcription proteins, lyases, oxidoreductases, isomerases, ligases, membrane proteins, viral proteins, chaperone proteins, and metalloproteins. A protein file with none of these keywords occurring in the header were categorized into a generic ”other” category. Protein sequences were extracted directly from the PDBBind dataset files using the SEQRES records, and sequences for proteins with multiple chains were concatenated using a column (:) symbol. Additionally, the SMILES strings for each of the ligands were extracted using the rdkit package^38^ from either the .mol2 or .sdf file provided in the original PDBBind dataset. The .mol2 files were used with higher priority due to their generally better description of bond orders in the ligands compared with the .sdf files in the PDBBind dataset. The latter were used instead when rdkit failed to read in .mol2 files.

### Identification of Covalent and Non-covalent Binders in PDBBind

It is important to treat covalent and non-covalent binders separately in PDBBind, because most existing algorithms that predict protein-ligand binding primarily focus on non-covalent interactions. As far as we are aware, there has been no systematic study of whether the binders in PDBBind are covalent or non-covalent. Relying on the CovBinderInPDB^39^ repository, we have identified covalent binders in the PDBBind dataset. The ligand names were extracted from the PDB files by comparing the minimum distance between any atom from the ligands in PDBBind database and residues in the PDB files downloaded from the RCSB database. If the minimum distance is less than 1Å, the matched residue name was compared with the record in CovBinderInPDB to identify whether the ligand is a covalent binder. If the minimum distance is more than 1Å or the residue name did not match the record in CovBinderInPDB, the structures were manually checked to identify whether the ligands are covalent binders or not. Ultimately 893 covalent binders and 18550 non-covalent binders were identified in the PDBBind dataset. The identifiers of the 893 covalent binders are reported in the Supplementary Information.

### Cleaning the PDBBind dataset

**Figure 1:**
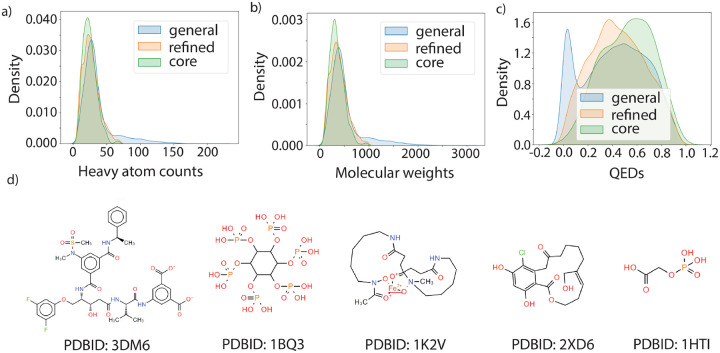
Property distributions and example ligands in the PDBBind dataset. (a) Distribution of number of heavy atoms, (b) molecular weights and (c) QED values for ligands in general, refined and core set of PDBBind dataset. (d) Example of ligand structures in the PDBBind dataset, with the corresponding PDBIDs

The majority of protein-ligand affinity prediction models are designed for drug-like ligands. However, a careful investigation of the ligands in the PDBBind dataset elucidated that not all the ligands are not necessarily drug like. Figure 1(a–c) shows the distributions of the number of heavy atoms, molecular weights and quantitative estimate of drug-likeness (QED)^40^ for ligands in the general, refined and core set in the PDBBind dataset. Some randomly sampled ligand structures are visualized in Figure 1(d). Specifically, some structures contain unusually large macrocycles, are peptide like, or contain long aliphatic chains. Although these protein-ligand complexes may still provide information about structure-affinity relationships, they may also contaminate the dataset and introduce non-transferable bias into the machine learning models trained on them. A small portion of the complex structures in PDBBind dataset also contained steric clashes, and there was one structure for which the ligand was far away from the protein (PDBID: 2R1W), and should be excluded from the dataset due to the low quality of the complex structures. The list of PDBIDs containing steric clashes (minimum heavy atom distance <1.75Å) are also provided in Supplementary Information.

Additionally, some proteins and ligands in the dataset contain uncommon elements. The proportion of occurrence for each element that occurred in the PDBBind dataset is summarized in Supplementary Table 1 for proteins and Supplementary Table 2 for ligands. It is difficult for machine learning models to learn protein-ligand interactions when uncommon elements are present in the PDBBind dataset. Therefore, we have defined a ”cleaner” version of PDBBind which only contains all data with ligand QED values larger than 0.2, protein and ligand elements that have at least 1‰ (more than 19 occurrences in the dataset), and minimum heavy-atom distance between protein and ligand fall within 1.75Å and 4Å. We call this subset of the data Clean Level 1 (CL1).

The binding affinity in terms of Δ*G* is directly related to the dissociation coefficient *K*_*d*_ or *K*_*i*_ through the formula Δ*G* = −*RT* ln(*K*).^41^ However, a large portion of the data in PDBBind is reported in terms of IC_50_, which cannot be easily translated to Δ*G*s due to its dependence on other experimental conditions and inhibition mechanisms.^42^ The IC_50_ values for the same protein-ligand complex can vary up to one order of magnitude in different assays. In addition, some data in PDBBind were not reported as exact values. Therefore, a second clean level (CL2) was defined on top of CL1, that additionally requires the target values are converted from records with “*K*_*d*_ = xxx” or “*K*_*i*_ = xxx”, to ensure the reliability of experimental binding free energy data. Finally, considering the original splitting of PDBBind into general/refined/core was based on structure quality,^29^ we also defined a third and highest quality level of data (CL3) that only retained data from refined and core set of PDBBind while still complying with all other quality-control metrics that we have defined. We did not use CL3 in this study, but identifiers of these protein-ligand complexes are available at https://github.com/THGLab/LP-PDBBind/tree/master along with the Cl1, Cl2 data sets.

### Similarity calculation of protein and ligands in the PDBBind dataset

Similarities for each ligand to any other ligand in the whole PDBBind dataset were calculated based on the Morgan fingerprints of the ligands using 1024 bits,^43^ and the Dice similarity is reported for the ligand pairs according to the following:^44^

(1) $$sim=\frac{2\left|A\cup B\right|}{\left|A\right|+\left|B\right|}$$

where |*A*∪*B*| counts the number of bits set to ON in the fingerprints of both ligands A and B, and |*X*| counts the number of bits set to ON in an single ligand X. A radius of 4 was first used when calculating Morgan fingerprints of the ligands; if the similarity between two ligands was calculated to be 1, it is recalculated using Morgan fingerprints of the ligands with radius of 10 to allow more careful validation of ligand identity. If the similarity was still 1 with the extended radius for calculating the fingerprint, the canonical SMILES string of the two molecules were compared and any discrepancy in the canonical SMILES will enforce the similarity to 0.99. These steps were designed to ensure ligands with similarity of 1 are strictly identical.

Similarities for proteins were calculated based on the aligned sequences of the proteins. Considering that it is unlikely two proteins belonging to different functional categories are similar, the sequence similarities were calculated only for proteins in the same category (i.e. transport proteins, hydrolases, etc.), and any two proteins that belong to different categories were defined to have similarity of 0. Within each category, every pair of protein sequences were aligned using the Needleman-Wunsch alignment algorithm,^45^ and the similarity was calculated as the number of aligned residues divided by the total length of the aligned sequence.

### New splitting of PDBBind dataset

We have formulated a new splitting of the PDBBind dataset to minimize the overlap between training, validation and test data as much as possible in order to eliminate the risk of data leakage. Data splitting was first done inside each protein category, and an iterative process was employed to separate the dataset step by step using the following algorithm.

In the first iteration, 5 complexes were randomly selected as seed data in the test set, and all data in the same protein category that have protein sequence similarity greater than 0.9 or ligand similarity greater than 0.99 were added to the test set as well. If protein-ligand complex data that have protein sequence similarity greater than 0.5 or ligand similarity greater than 0.99 were found, they too were added to the validation set. Any data newly added to the test set in this iteration will become seed data in the next iteration, and the iteration continues until no new data are added to the test set. Next, a similar process was applied to the validation data, adding all remaining data that have protein sequence similarity greater than 0.5 or ligand similarity greater than 0.99 into the validation set. The remaining data is then defined as the training set.

The number of training, validation and test data under the LP-PDBBind in each category is reported in Table 1. After data splitting by category, the protein-ligand complexes for training, validation, and testing were combined to define the splitting for the whole dataset. However, after combination the ligand similarity might still exceed 0.99 for data from different categories. Therefore, any data in the combined training set that has ligand similarity greater than 0.99 to any other data in the validation or test set were discarded altogether. The resulting number of data in the training, validation, and test set were 11513, 2422 and 4860, respectively, after this cleaning step. The new splitting procedure ensures the data in the training set has a maximum protein sequence similarity of 0.5 and maximum ligand similarity of 0.99 to any data in the validation or test datasets, and the maximum similarity between any validation and test data is 0.9 for protein sequence similarity and 0.99 for ligand similarity.

### Compilation of the BDB2020+ dataset

Because many of the recent SFs have been trained on PDBBind, which means utilizing a subset of PDBBind as the test dataset risks data leakage, we have created a new benchmark dataset that is independent of PDBBind. To fulfill the need for a fair benchmark, we looked for the latest records deposited in BindingDB,^35,46^ one of the largest public binding affinity repositories, which provides additional experimental conditions including assay information, pH and temperature. However, it does not guarantee each record has an associated 3D complex structure.

We have developed a workflow to match complex structures in RCSB PDB with records in BindingDB, and the flowchart is illustrated in Supplementary Figure S1. Starting with the original BindingDB dataset, the records with potential matched structures in the RCSB database were identified by searching the InChi keys that occur in BindingDB to find all PDB records that contain the same ligand, and then further filtering based on the PDBID of the target chain that was also recorded in the BindingDB dataset. Release dates of the PDB structures were extracted from the RCSB PDB records and only records after year 2020 were selected for further processing. Additionally, we employed the same similarity criterion as what was used in developing LP-PDBBind, and removed all data that has sequence similarity greater than 50% or ligand similarity greater than 99%.

The filtered PDB files were downloaded from the RCSB database and small molecule ligands were extracted from the PDB files. Since a complex may contain multiple ligands, but only one matches the record in BindingDB, we selected the ligand in the PDB that has the best structural match with the SMILEs provided in BindingDB, and ensuring that the number of heavy atoms is exactly the same. We then used rdkit to reassign bond orders to the extracted ligands using the BindingDB SMILES as reference. This step was necessary because bond orders are usually not present in a PDB file and are typically inferred from local atomic geometries, which sometimes result in unreasonable bonding structures; thus the bond order reassignment step ensures the rationality of the processed structures. Additionally, any chain that is within 5Å of the ligand was compared with the interacting chain sequence in BindingDB record. A reliable match was only made when the consecutive aligned residues are exactly the same. The reason to keep strict alignment criterion is that if the protein contains mutations, the binding affinity might change significantly, in which case the BindingDB record will not represent the true binding affinity for the complex structure in the PDB and is not usable for the benchmark. After discarding all unmatched data, we obtained 130 data records, out of which 115 contains accurate binding affinity data, and defines the BDB2020+ test dataset.

### Model retraining procedures

All models were retrained using the CL1 version of the LP-PDBBind training dataset to achieve balance between data cleanliness and the amount of training data available. For model validation and testing, the non-covalent LP-PDBBind validation and test data at CL2 were used to ensure data quality is higher, and can be considered as an optimistic estimation of the performance of these models on high quality data.

### AutoDock Vina Retraining

For each molecule in the LP-PDBBind training set, the 6 individual terms (gauss1, gauss2, repulsion, hydrophobic, hydrogen, rot) of AutoDock Vina^3^ were calculated using the vina binary by setting weight of one term to one and the rest of the weight to 0. The weights of these six terms were optimized by minimizing the mean absolute error between the weighted sum of six terms and binding free energy through the Nelder-Mead optimization algorithm.^47^ The final retrained AutoDock Vina parameters are provided in Supplementary Table 4 and all further evaluations of retrained models are done with these modified weights.

### RF-Score Retraining

RF-Score (RF) is a Random Forest model that predicts binding affinities from the number of occurrences of a particular protein-ligand atom type pair interacting within a certain distance range.^14^ We here followed the RF-Score-v1 approach, where nine common elemental atom types (C, N, O, F, P, S, Cl, Br, I) for both the protein and the ligand were considered and neighboring contacts between a protein-ligand atom pair were defined within 12 Å. For fairness of comparison, we trained a RF model on the PDBbind2007 refined set, denoted as the original model, and on the LP-PDBBind training data, as the retrained model, using the same script.

### IGN Retraining

InteractionGraphNet (IGN) utilizes two independent graph convolution modules to sequentially learn the intramolecular and intermolecular interactions from the 3D structures of protein-ligand complexes to predict binding affinities.^15^ The code for retraining the IGN model (https://github.com/THGLab/LP-PDBBind/tree/master/model_retraining/IGN) was adapted from the original training scripts using the same feature size and layer numbers as the original published model with only the training data modified. Due to molecule generation errors in RDKit,^38^ which is required for featurizing the 3D structures into graph representations, only 7277 complexes from the LP-PDBBind training set were used.

### DeepDTA Retraining

DeepDTA is a Y-shaped 1d convolutional neural network that takes in protein sequences and ligand isomeric SMILES strings as input and outputs the predicted binding affinities.^18^ Since the method did not originally train on the PDBBind dataset, we retrained the model with the original PDBBind general and refined sets using a PyTorch implementation of the original code: https://github.com/THGLab/LP-PDBBind/tree/master/model_retraining/deepdta. More specifically, we did a 90–10 train-validation split on the data for training and then tested its performance on the core set. Different protein and ligand kernel sizes were used as suggested by the original work and the best-performed parameters were chosen to serve as the original model in our performance comparison tables. The retrained model with LP-PDBBind was also generated following the same scheme.

Since the 1d convolution requires a fixed size of the protein sequence and ligands, during all training processes, proteins with sequence lengths longer than 2000 and ligands with isomeric SMILES lengths longer than 200 were discarded, resulting in a loss of around 100 protein-ligand pairs. Also, since the ligand encoding was based on the training set only, all ligands with unseen tokens from their isomeric SMILES strings in the test set and real-world examples are discarded, resulting in a loss of around 15 data points. It is worth noting that these losses of data happen to both the original model and the retrained model, so it won’t affect our conclusions in this work.

## Results

### Analysis of PDBBind Splittings

**Figure 2:**
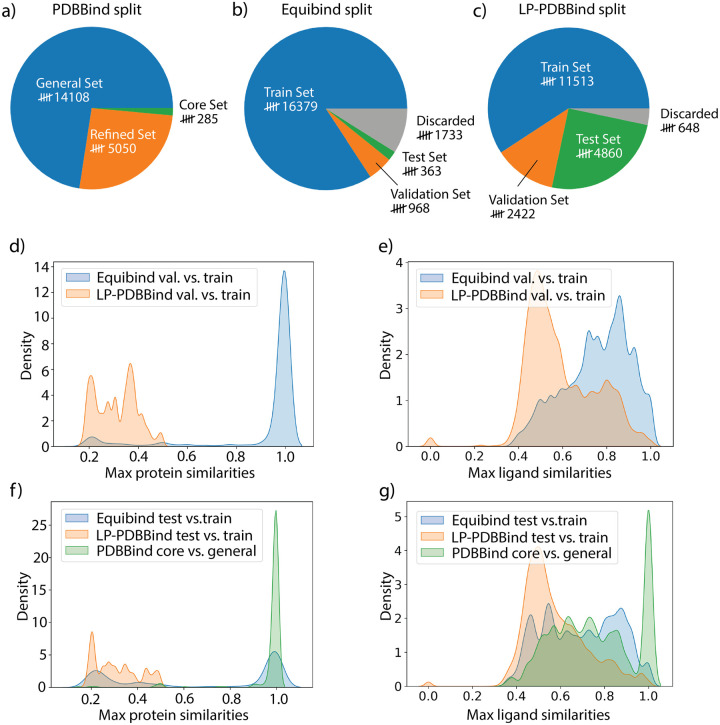
Data statistics under different splits of the PDBBind dataset. Number of data in different splits of the PDBBind dataset: (a) general, refined and core set in original PDBBind split; (b) train, validation, test set and discarded data in Equibind split; (c) train, validation, test set and discarded data in LP-PDBBind. Comparison of (d) maximum protein similarities and (e) ligand similarities between validation set and train set under Equibind split and LP-PDBBind. Comparison of (f) maximum protein similarities and (g) ligand similarities between test set and train set (or core set and general set in original PDBBind split) under different splittings.

Data distributions of PDBBind under the original split (general set/refined set/core set), Equibind Split (train/validation/test) and LP-PDBBind (train/validation/test) are provided in Figure 2 a–c. LP-PDBBind has significantly extended the size of the test set compared with the original PDBBind split and the more recent Equibind split. A bigger test set provides more accurate evaluation of model performance when the model is applied to data that has not been trained on. By contrast, the number of data in the training set is much smaller in LP-PDBBind. As we will show later, the shrinkage in the training set is necessary, because it keeps the similarity with validation/test set sufficiently low. Compared with the Equibind split, we have also decreased the number of discarded data, because we only discard data when they are highly similar to any other data in train, validation or test set.

One of the main purpose of defining the new split of PDBBind is to prevent data leakage between training, validation, and test data. To understand whether the new split has solved the data leakage issue, the maximum similarity for proteins and ligands between the training, validation, and test data under the EquiBind split and LP-PDBBind, and maximum similarity between the general set and core set under the original PDBBind split are summarized in Figure 2(d–g). The original PDBBind split has significant protein and ligand overlap between the general and core sets, as can be seen in the sharp peak at similarity of 1.0 in Figure 2(f–g). The same level of similarity between the refined and core sets in the original PDBBind split are shown in Supplementary Figure 2. Since many machine learning models use the PDBBind core set as the test dataset without carefully excluding similar data from their respective training dataset, the performance of these models reported may be overly optimistic and do not reflect their true generalizability and needs to be reevaluated.

The Equibind split is a significant step forward in reorganizing the PDBBind data more reasonably. Its time-based cutoff in defining the test dataset decreased the chance of data leakage, but still did not eliminate the possibility of highly-similar data that occurs in both train and test set. By comparison, LP-PDBBind minimizes overlap between data in the train and test set by design, keeping the maximum sequence similarty between any protein in the test set and any protein in the train set below 0.5, and the maximum ligand similarity below 0.99 (Figure 2(f–g)). Therefore, results for a model trained with LP-PDBBind train set and evaluated with LP-PDBBind test set should better reflect the performance of the model when applied to a new protein-ligand complex that may be very different than data used for training the model.

To help with training more transferable models, we have also defined the validation set to be equally different than the train set as the test set. Figure 2(d–e) illustrates the maximum protein similarities and maximum ligand similarities between the validation and training sets for the Equibind split and LP-PDBBind, respectively. The similarity between validation and training sets under LP-PDBBind is also well controlled so that the validation set can be used to select the most transferable model or hyperparameters when training the model. By comparison, the validation set in Equibind split is too similar to the train set, hence overfitting will not be effectively captured when monitoring model performance on the validation set. As is provided in Supplementary Figure 3, both the Equibind and LP-PDBBind splits of PDBBind, the validation and test set have a wide range of (dis)/similarities on proteins and ligands. Therefore using the validation set for model selection will not lead to overfitting and thus increase transferability to the test set.

### Evaluating Retrained Models with LP-PDBBind

The LP-PDBBind CL1 cleaned data for non-covalent binders of PDBBind were used to retrain Autodock vina,^3^ IGN,^15^ the 2010 RF-Score^14^ and DeepDTA.^18^ These models cover a wide range of different approaches for scoring, and are representative of different dimensions of the SF space of models. AutoDock vina is a PSF that contains molecular interaction terms consisting of a van der Waals-like potential (defined by a combination of a repulsion term and two attractive Gaussians), a nondirectional hydrogen-bond term, a hydrophobic term, and a conformational entropy penalty, all of which are weighted by empirical parameters.^3,48^ The other three methods belong to MLSF category, but vastly differ in their feature set. The original RF-Score model utilizes the occurrence count of intermolecular distances between elemental atom types of protein and ligands based on 3D structures, and utilizes a random forest model to make predictions on binding affinities.^14^ IGN utilizes a graph neural network operating on the 3D complex structures, and the node and edge features are straightforward information about atoms and bonds, including atom types, atom hybridization, bond order, etc.^15^ Finally, DeepDTA is a purely data-driven approach which does not rely on physical interactions or 3D structural information. Instead, it uses 1D convolutions on the string representations of the proteins and ligands to make predictions.^18^ After retraining, the new AutoDock vina, RF-Score, IGN, and DeepDTA model performances are compared with the old models as tested on the non-covalent LP-PDBBind test set using the CL2 data, the BDB2020+ new benchmark data, and the Mpro and EFGR applications.

Table 2 provides the root mean square error (RMSE) of the binding affinity prediction (Δ*G*_*bind*_) on the training, validation, and test data for the models retrained with LP-PDBBind, and comparing it to the original models and their performance on the LP-PDBBind test data. Supplementary Figure 4 shows the scatter plots of the same data and reports the correlation coefficient between predicted and experimental binding affinities. Due to the data leakage issue, performance of the original models on the LP-PDBBind test data are over-estimated for the MLSFs. By comparison, AutoDock vina due to its small number of trainable parameters does not suffer from the data leakage issue, and has achieved lower RMSE after retraining. Among the MLSF models, IGN has the smallest generalization gap, and is also the best performing model when evaluated using the LP-PDBBind test set. Since random forest models can overfit training data relatively easily,^27^ the RF-Score model has exceptional performance on the LP-PDBBind training dataset, but its validation and test performance is slightly worse than the IGN model. DeepDTA also performs quite well on the training dataset, but exhibits a large generalization gap with respect to the validation dataset, and has similar performance as AutoDock vina on the test dataset. Overall the original MLSF models have seen some of the LP-PDBBind test proteins and ligands in their training, and thus they appear to perform better than they actually do when data leakage is controlled for using the LP-PDBBind data.

**Figure 3:**
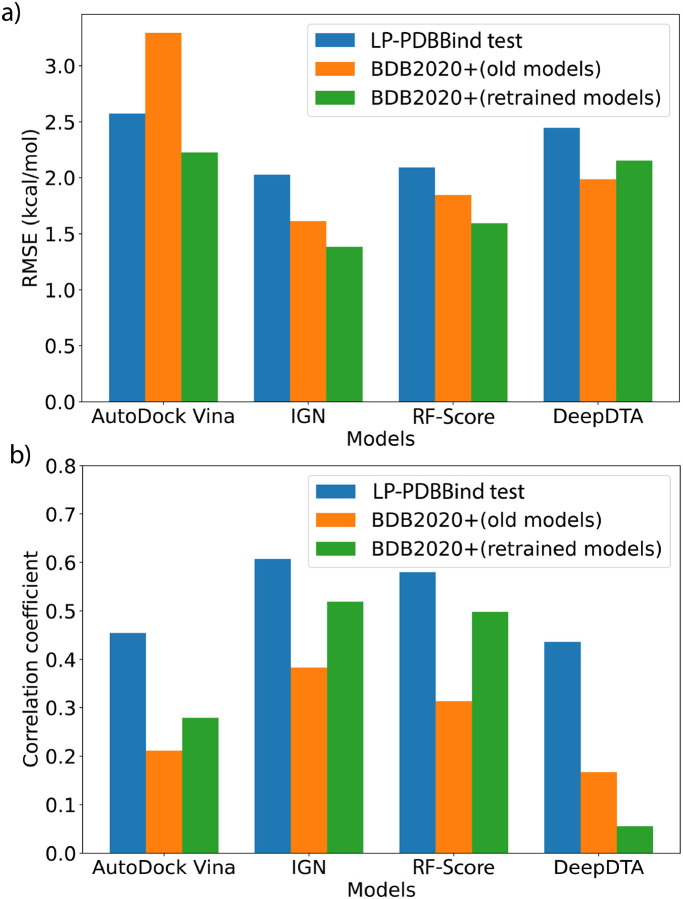
Performance comparisons using different models and different benchmark datasets. (a) Comparison on the root mean square error (RMSE) for different models. Lower is better. Blue bars indicate RMSEs on the LP-PDBBind test dataset using retrained models, orange bars indicate RMSEs for the models without retraining using LP-PDBBind, and green bars indicate RMSEs for the models retrained using LP-PDBBind. (b) Comparison on the Pearson correlation coefficient (*R*) for different models.

Figure 3 and Table 3 summarize the scoring performance of the original and retrained models on the independent BDB2020+ benchmark dataset; the corresponding scatter plots for the original and retrained models are provided in Supplementary Figure 5. We see that upon retraining, the three models based on 3D structures have all improved significantly on the BDB2020+ benchmark set. In terms of RMSE, AutoDock vina decreased by 1 kcal/mol while IGN and RF-Score decreased by 0.2–0.3 kcal/mol. The changes in the correlation coefficients are more profound: both IGN and RF-Score have achieved absolute correlation coefficients better than 0.5, and relative improvements for AutoDock vina and IGN increased by 30–40%, and RF-Score improved by more than 60%. On the other hand, the performance of DeepDTA declined after retraining. The RMSE has increased by 8% and the retrained model has almost no correlation with experimental measurements. It is reasonable to expect a purely sequence based method to not perform well when the training dataset contains more variety, since it poses a much greater challenge for the model to find the patterns of the interaction modes between the protein and ligand from sequence information alone. But for the 3D structure based models, a diverse training dataset actually helps with finding more transferable features, which leads to the overall performance enhancements on the BDB2020+ evaluation dataset.

Figure 3 also compares the performance of the four models on BDB2020+ with the results evaluated on the LP-PDBBind test dataset. Interestingly, we find that all models have achieved lower RMSE on the BDB2020+ dataset but also lower Pearson correlation coefficients, *R*. This seemingly contradictory result is actually reasonable due to the dataset distribution differences of the two evaluation benchmarks. The LP-PDBBind test dataset contains much more data than BDB2020+, and also spans a wider range of binding affinity values. The measured −log(*K*_*d*_) values in the LP-PDBBind test dataset ranges from 0 to 12 (i.e. 12 orders of magnitude), but the BDB2020+ dataset only ranges from 4 to 10. Given that extreme predictions from a robust ML model is unlikely, a narrower range of binding affinities means the overall error will be smaller. However, it also poses challenge for successfully differentiating the nuances between more clustered data points, and therefore it is also more difficult to achieve higher correlation coefficients.

Nevertheless, we see that the relative rankings of the four methods are consistent between different evaluation benchmarks and different metrics. IGN and RF-Score are performing better than AutoDock vina, and DeepDTA is not as competitive to the other models that use 3D structural information. In terms of a generalization gap to new data, we also see the correlation differences are the smallest for IGN and RF-Score, while DeepDTA has a huge generalization gap. These results are consistent with the overall performances of the models, and provide evidence that modern MLSFs can indeed surpass PSFs such as AutoDock vina, even when protein and ligand similarities are low. However, 3D information is essential to ensure that the model has transferable performance.

### Evaluating the Ranking Capabilities of the Retrained Models

The improvement in scoring power, despite being important, still does not fully reflect the performance capability of the SFs in real world applications. Therefore, we have prepared two additional datasets of protein-ligand complexes with the same protein and different ligands to evaluate their ranking accuracy of the SFs before and after retraining using LP-PDBBind. The first case is the SARS-CoV-2 main protease (Mpro) for which a wide variety of potential Mpro inhibitors have been developed.^36^ We have manually extracted published co-crystal structures of Mpro with a number of non-covalent inhibitors,^49–61^ and prepared a dataset containing 40 structures and corresponding experimental binding affinity measurements. The second dataset involves the epidermal growth factor receptor (EGFR), which is a receptor tyrosine kinase related to multiple cancers including lung cancer, pancreatic cancer and breast cancer.^37^ Similarly, we have selected 23 representative non-covalent protein-ligand complex structures of EGFR with binding affinities taken from BindingDB.^62–76^

**Figure 4:**
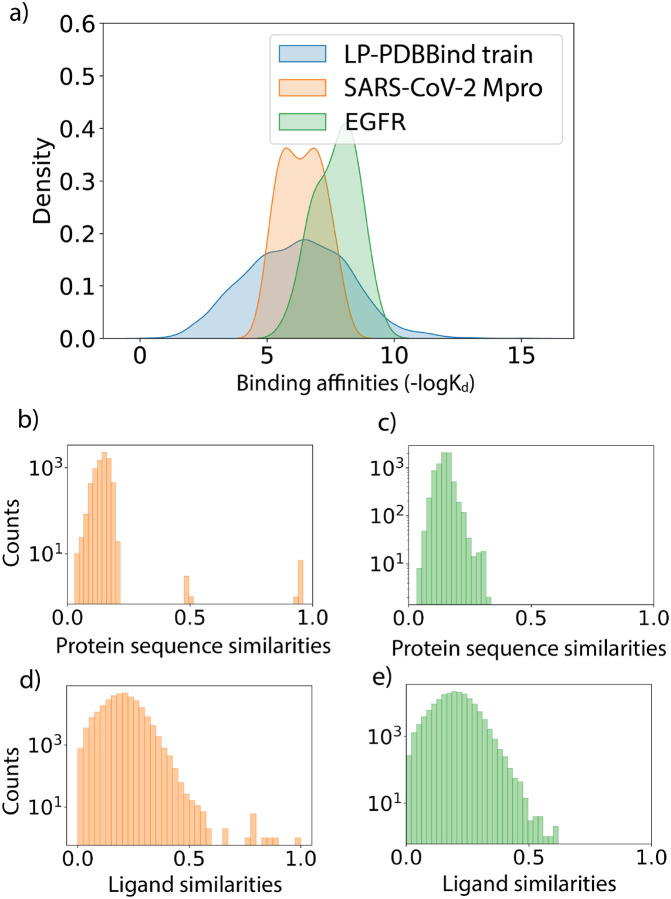
Data statistics for the SARS-CoV-2 main protease (Mpro) benchmark set and epidermal growth factor receptor (EGFR) benchmark set. (a) Distributions of the binding affinity data (−log*K*_*d*_) in the LP-PDBBind train dataset in blue, Mpro benchmark set in orange and EGFR set in green. (b-c) Distributions of protein sequence similarities between the Mpro protein (b) and EGFR protein (c) with proteins in the LP-PDBBind train dataset. (d-e) Distributions of ligand fingerprint similarities between molecules in the Mpro benchmark set (d) and EGFR benchmark set (e) with ligands in the LP-PDBBind train dataset.

Figure 4 shows the distributions of experimental free energies and protein and ligand similarities when comparing the LP-PDBBind training data with the Mpro and EGFR datasets. The binding affinities for the Mpro and EFGR systems are found to be in a narrower range than the LP-PDBBind training data. While the average binding affinity of the Mpro dataset is roughly in line with the LP-PDBBind training dataset, the ligands in the EGFR dataset have an overall tendency to bind stronger to their targets. This shift in the average indicates that the EGFR is more ”out-of-distribution” than the Mpro dataset. Figure 4b–e further show that the protein and sequence similarities of the two evaluation datasets are overall very dissimilar to the LP-PDBBind training dataset, with the exception of a small fraction of protein and ligand sequence similarity attributable to the SARS-CoV-1 Mpro training entry PDBID: 3V3M.^77^ Hence the Mpro and EFGR evaluations reflect two representative scenarios of using the SFs on a similar or different protein-ligand system than what was included in the PDBBind dataset.

Table 4 summarizes the RMSE, Pearson correlation coefficients (*R*) and Spearmann correlation coefficients (*R*_*S*_ or ranking power) for the data in the Mpro and EGFR evaluation sets using both the original models and the models retrained using LP-PDBBind. The prediction scatter plots for the Mpro benchmark dataset and EGFR benchmark dataset are provided in Supplementary Figures 6 and 7. For the Mpro dataset, AutoDock vina and RF-Score exhibit some modest improvement in all the metrics. The RMSE on the retrained IGN model decreased by 0.42 kcal/mol, but the correlations in terms of *R* and *R*_*S*_ have also become slightly lower. The performance of the DeepDTA model is largely unchanged on the Mpro dataset. Taken all metrics into account, AutoDock vina is the best performing SF for the Mpro dataset, although the differentiation among all models is not large.

However, the results on the EGFR dataset exhibit much larger variations for the different models, and the changes are significant for all the SFs. There is a dramatic decrease by ˜50% in RMSE for AutoDock vina, and both *R* and *R*_*S*_ have increased more than 50%. IGN has a slight decrease in RMSE but has increased remarkably in the correlation coefficients. The retrained IGN model achieves 0.65 in Pearson correlation coefficient and 0.62 in Spearmann correlation coefficient, which is the highest among all models, and does not differ much with the Mpro results and thus showing good generalizability. RF-Score has also benefitted from retraining quite significantly, with a 0.6 kcal/mol decrease in RMSE, and the correlation coefficients have improved from −0.15 to 0.52 in terms of *R* and from −0.18 to 0.45 in terms of *R*_*S*_. The importance of 3D information is also illustrated by finally considering a method that ignores it, in which DeepDTA performs relatively comparable to the other SFs for the Mpro dataset with *R*_*S*_ = 0.66, but with *R*_*S*_ = 0.23 for the EGFR dataset. Overall, the performance rankings of the four SFs are still consistent with that obtained from the LP-PDBBind test set benchmark and the BDB 2020+ benchmark. But the EFGR benchmark emphasizes that new applications will benefit from better generalizability of the newly retrained models, and that 3D information is vital for exploiting the PDBBind data.

## Discussion and Conclusion

The area of computational drug discovery relies on generalizable scoring functions that have robust scoring and ranking power of binding affinities of ligand-protein complexes. However due to the lack of independently built datasets that test the true generalizability of the SFs, it is hard to differentiate among the plethora of many models which have been trained and tested on the original split of the PDBBind dataset. As we have shown, there is too much overlap between the PDBBind general and refined data used for training with the core subset, leading to the possibility of inflated performance metrics that in turn lead to false confidence in how such models will perform on new protein-ligand complexes.

In order to reduce data similarity between training, validation, and test data of the PDBBind dataset, we have developed LP-PDBBind using an iterative process to select most similar data first into the test set, and then validation set, so that the final training dataset has low similarity with validation or test dataset. We have also cleaned the PDBBind data in multiple ways: CL1 removed covalent ligand-protein complexes, the low populations of drug molecules with underrepresented chemical elements, and complexes with steric clashes. In addition to CL1, the CL2 level of cleaning aimed for consistent measures of binding free energies by either converting *K*_*d*_ or eliminating data reported as IC_50_. Finally, CL3 eliminated the general set to perform splits on the refined and core set data which is deemed of higher quality. However, it is always a trade-off between the quality of data and the amount of data, and all of the results reported here were based on training on CL1 data and testing on CL2. One possibility to achieve better performance in the future is to train a base model using the larger amount of data with CL1, and then fine-tune using the least available but higher quality data such as CL3. In addition we have isolated the covalent binding data from PDBBind that may help newly formulated SF predict these type of protein-ligand complexes.

It is important to understand whether the improved performance of latest SFs are due to better methodologies that can truly generalize to unseen data. This is especially true for MLSFs because their complex architecture and large amount of parameters allow them to memorize data in the training dataset, which if leaked to the test will obscure the benchmarks. To provide a benchmark dataset truly independent of PDBBind, we have compiled the BDB2020+ dataset derived from the BindingDB database deposited after 2020, and further ensuring that there is no overlap with PDBBind. Additionally, we also tested ranking power through construction of the Mpro and EFGR ligand-protein complex series. The SARS-CoV-2 Mpro protein has high similarity counterparts in the PDBBind dataset, and the other series involving EGFR does not have anything similar in the training dataset of PDBBind. These new data should also be useful in future evaluation and/or finetuning of any scoring function.

In this work we utilized the new split of PDBBind using CL1 to retrain AutoDock vina, IGN, RF-Score and DeepDTA and compared the old models with the retrained models on the LP-PDBBind test set, as well as the fully independent BDB2020+ data, Mpro series, and EGFR series. We have demonstrated that using a different splitting of the same dataset leads to significant performance improvements, but the SF has to rely on features that take into account the 3D structures. Furthermore, we have shown that retrained MLSFs can indeed surpass traditional PSFs, even when protein and sequence similarities are low. The comparisons between the different benchmark results for the SFs can be well explained by the ”difficulty level” of these datasets, and provide insights about the generalizability of various models. We found that well performing models also have more stable ranking results for different ligands towards a protein target. When the target system is similar to data included in the training dataset, the differentiation between models are not obvious. However, when the protein target is not similar to anything in the training dataset, we found that different SFs demonstrate quite different generalization capabilities, and IGN model retrained with LP-PDBBind is recommended due to its reliable good scoring and ranking power.

Given that the benchmarks are done with experimental protein-ligand complex structures, the superior performance of the retrained models do not necessarily mean they also have better docking power for recognizing native ligand bound poses. Neverthless, the improvements shown for scoring power and ranking power are meaningful, because the virtual screening process can be broken into multiple steps, and we could use one SF for docking and another for scoring. Furthermore, it is reasonable to expect SFs trained with LP-PDBBind will also have better generalizablity in terms of docking or screening capabilities, and it would be worthwhile to generate decoy structures for the BDB2020+ dataset to better benchmark the docking and screening powers of the SFs independent of the PDBBind dataset. In summary, the cleaned LP-PDBBind data in its current form is a valuable resource for training more transferable SFs, and we hope reporting evaluation metrics on the BDB2020+ dataset can also become a common practice for future SFs.

## Supplementary Material

1

## Figures and Tables

**Table 1:** Number of train, validation and test data in each protein category before merging for the new split of PDBBind

Protein type	Train	Validation	Test
hydrolase	4038	318	1377
transferase	2226	1228	1837
other	3050	405	134
transcription	642	52	298
lyase	331	71	465
transport	503	35	83
oxidoreductase	342	52	182
ligase	342	38	90
isomerase	199	70	64
chaperone	50	66	184
membrane	157	56	71
viral	196	18	53
metal containing	85	13	22

**Table 2:** The training and test errors for the original and retrained models using LP-PDBBind for AutoDock vina, IGN, RF-Score, and DeepDTA in terms of root mean square error (RMSE) in kcal/mol

Model	RMSE Original	RMSE Retrained		
	test	train	validation	test
AutoDock Vina	2.85	2.42	2.29	2.56
IGN	1.82	1.69	1.93	2.03
RF-Score	1.89	0.68	2.13	2.09
DeepDTA	1.34	0.64	2.74	2.45

**Table 3:** Performance comparisons on the BDB2020+ benchmark set in terms of root mean square error (RMSE) and Pearson correlation coefficient (*R*) for different models before and after retraining using LP-PDBBind.

Model	RMSE (kcal/mol)			*R*		
	original	retrained	difference	original	retrained	difference
AutoDock Vina	3.29	2.23	−32%	0.21	0.28	+33%
IGN	1.61	1.38	−14%	0.38	0.52	+37%
RF-Score	1.84	1.59	−14%	0.31	0.50	+61%
DeepDTA	1.99	2.15	+8%	0.17	0.06	−65%

**Table 4:** Performance comparisons on the SARS-CoV-2 main protease (Mpro) and epidermal growth factor receptor (EGFR) benchmark set in terms of root mean square error (RMSE) in kcal/mol, Pearson correlation coefficient (*R*) and Spearmann correlation coefficient (*R*_*S*_) for different models before and after retraining using LP-PDBBind.

Mpro Model	RMSE		*R*		*R*_*S*_	
	original	retrained	original	retrained	original	retrained
AutoDock Vina	1.20	1.17	0.55	0.66	0.51	0.68
IGN	1.86	1.44	0.64	0.61	0.69	0.65
RF-Score	2.06	1.64	0.43	0.52	0.47	0.58
DeepDTA	1.18	1.12	0.59	0.63	0.60	0.66
EFGR Model	RMSE			*R*		*R*_*S*_
	original	retrained	original	retrained	original	retrained
AutoDock Vina	3.11	1.59	0.25	0.38	0.21	0.36
IGN	1.06	0.96	0.36	0.65	0.17	0.62
RF-Score	1.57	0.97	−0.15	0.52	−0.18	0.45
DeepDTA	1.22	1.38	0.23	0.06	0.20	0.23
