# Leak Proof PDBBind: A Reorganized Dataset of Protein-Ligand Complexes for More Generalizable Binding Affinity Prediction

**Published:** 2024-05-03

**Authors:** Jie Li, Xingyi Guan, Oufan Zhang, Kunyang Sun, Yingze Wang, Dorian Bagni, Teresa Head-Gordon

## Abstract

Many physics-based and machine-learned scoring functions (SFs) used to predict protein-ligand binding free energies have been trained on the PDBBind dataset. However, it is controversial as to whether new SFs are actually improving since the general, refined, and core datasets of PDBBind are cross-contaminated with proteins and ligands with high similarity, and hence they may not perform comparably well in binding prediction of new protein-ligand complexes. In this work we have carefully prepared a cleaned PDBBind data set of non-covalent binders that are split into training, validation, and test datasets to control for data leakage, defined as proteins and ligands with high sequence and structural similarity. The resulting leak-proof (LP)-PDBBind data is used to retrain four popular SFs: AutoDock Vina, Random Forest (RF)-Score, InteractionGraphNet (IGN), and DeepDTA, to better test their capabilities when applied to new protein-ligand complexes. In particular we have formulated a new independent data set, BDB2020+, by matching high quality binding free energies from BindingDB with co-crystalized ligand-protein complexes from the PDB that have been deposited since 2020. Based on all the benchmark results, the retrained models using LP-PDBBind consistently perform better, with IGN especially being recommended for scoring and ranking applications for new protein-ligand systems.

## Introduction

Scoring functions (SFs) are crucial in computer aided drug discovery, utilized for selecting the most probable binding pose geometry and/or free energy of binding between a ligand and a protein.[1,2] There are a plethora of SFs being developed and widely used by computational chemists, but they can be broadly categorized into either classical scoring functions (CSFs),[3–13] or machine learning scoring functions (MLSFs).[14–21] CSFs such as the commonly used AutoDock Vina[3] are designed to model intermolecular interactions or missing free energy components, and can benefit both from better design of the functional form, many-body physics, as well as the availability of training data for parameterization of their semi-empirical or empirical functions.[3–9] Data-driven MLSFs by contrast are less reliant on physical interaction modeling and are far more dependent on experimental information,[11,22,23] culminating in the current development and use of sophisticated machine learning (ML) models[14–21] whose much larger parameter spaces are optimized on large, high quality datasets. Some representative MLSF designs include Random Forest Score (RF-Score)[14] that is based on atom-pair distance counts, InteractionGraphNet (IGN)[15] that uses graph neural network (GNN) to represent raw 3-dimensional protein and ligand structures, and DeepDTA[18] that predicts binding affinities using amino acid sequences of the proteins and SMILES strings of the ligands. More recent models have been proposed that achieve better accuracy at binding affinity prediction, including RFScore-V3[24] that incorporates terms from AutoDock Vina, and also PIGNet,[16] RTMScore[17] and GIGN[21] which are more advanced GNN-based models.

The majority of protein-ligand interaction SF predictors, whether physical or machinelearned, have been trained on the PDBBind dataset.[25] The Comparative Assessment of Scoring Functions (CASF) benchmark, which assesses the scoring power, ranking power, docking power and screening power of various SFs, was also conducted on the PDBBind core set.[26] More specifically PDBBind is a curated set of ∼20K protein-ligand complex structures and their experimentally measured binding affinities, in which the "general" and "refined" data subsets are used for training, and a separate "core" set is used for testing. However, not all protein-ligand complex structures in the general and refined data sets are as high-quality as those in the core set which contains protein-ligand complexes with the best structural resolution and most reliable binding affinity data.[25] Additionally, the average size of the ligands in the core set is also smaller than that in the rest of the PDBBind dataset, which may make the core set easier prediction targets. Furthermore, as we will show later, the majority of the core data records in the PDBBind dataset have identical proteins and/or ligands with that found in the general and/or refined sets. As such, most empirical SFs and MLSF models have been trained with significant data leakage, and thus their reported performance on the core set is only a true measure of new protein-ligand complexes with high similarity, but will inevitably have limited transferability to low-similarity ligand-proteins scenarios.

The definition of data leakage in PDBBind is not as literal a scenario in which the *exact* occurrence of input representation occurs in both training and test sets. Instead data leakage here refers to highly similar protein or ligands measured by sequence and chemical similarity and similar structure occurring in both the training and testing datasets. This will lead to artificially high evaluation results that do not fully reflect the performance of the models in real world applications.[27] Therefore, the most standard data splitting protocol in the machine learning community, which is to randomly split the dataset into train, validation and test subsets will inevitably introduce significant data leakage issue. Given that many MLSFs training on PDBBind do not carefully investigate the data similarity issue, whether these new MLSFs have truly surpassed traditional CSFs in actual predictive performance is still an open question, or how well any given CSF or MLSF performs on new protein-ligand binding applications.[28–31] It is already known that any SF model can be overtrained so that it has exceptional performance on the training dataset, a problem which can be mitigated by several known regularization strategies to provide better generalization to an independent test set.[32,33] Equally important, however, is to control for data leakage into the test set itself, without which can lead to false confidence in predictive capacity when the training dataset has high similarity to the test set, but manifests as poor generalization when the sequence or structure similarity is low.[29]

Two typical solutions have been taken to mitigate this data leakage issue: one is to perform data splits based on scaffolds, protein families or protein clusters, and the other is to perform time-based cutoff which was proposed on earlier versions of PDBBind,[24] and was suggested by the authors of the EquiBind model on the PDBBind 2020 release.[34] The scaffold or target based splitting can make sure the proteins in the complex are not similar between training and testing datasets, but they usually do not take the ligand similarities into account. The idea of a time-based splitting mimics a "blind test" setting, by which the model can only be trained with data released in year 2019 or earlier, and predictions are made with data after year 2019, so that the test data will never be covered by training data. However, since new drugs are being developed that can interact with popular protein targets that have been established for years,[35,36] and existing drug molecules may also be tested on new proteins,[37] there are still frequent encounters with almost identical proteins or ligands in the latest experiments with earlier assays. As such, a time based splitting of the dataset is still not an ideal solution.

For the purpose of retraining SFs that can either truly generalize, or just as importantly to anticipate when it will not work well when similarity of protein-ligand complexes is low, we build a new data split to mitigate data leakage of highly similar sequence and structures of both proteins and ligands in the training and test set. In this work we reorganize the PDBBind data into new train, validation and test datasets which we call Leak Proof PDB-

Bind (LP-PDBBind), by minimizing sequence and chemical similarity of the proteins and ligands between the datasets as well as making sure the protein-ligand structural interaction patterns are different among the data splits as well. Our new data splitting can be regarded as an improvement over just a protein scaffold split by providing similarity control on the ligands as well, which we hypothesize will decrease the chance of a model making predictions by memorizing ligand structures.[38,39] Furthermore we have cleaned the PDBBind data to eliminate covalent bound ligand-protein complexes (thus focusing on non-covalent binding), certain ligands with very low frequency occurrences of certain atomic elements, to remove obvious steric clashes, as well as maintaining consistency in reported binding free energies and their units.

We then use the cleaned LP-PDBBind data for the development of new versions of several foundational SFs, including AutoDock Vina,[3] RF-Score,[14] IGN,[15] and DeepDTA.[18] This provides a standardized way to train and benchmark different SFs with the exact same train, validation and test data. Furthermore, in order to provide a true independent benchmark for the new SFs that result from retraining using the cleaned LP-PDBBind, we created a new evaluation dataset, BDB2020+. BDB2020+ is compiled based on entries into the BindingDB dataset[40] that were deposited after 2020, and further filtered according to the same similarity control criteria used for the development of the new LP-PDBBind. As a further test of ranking power, we additionally prepared two sets of experimental binding affinity data for different ligand complexes of the SARS-CoV-2 main protease (Mpro)[41] and epidermal grow factor receptor (EGFR),[42] neither of which was included in the LP-PDBBind training dataset (although Mpro has similar SARS-CoV-1 protease proteins in PDBBind). Finally, we perform two control evaluated the performance of retrained SFs on docked structures using the old Vina SF to mimic a more typical drug discovery scenario.

The results show that all SFs trained on PDBBind show better performance on the LP-PDBBind test set due to data leakage, while the retrained models using the LP-PDBBind split are found to give a more true measure of performance. Furthermore, the newly trained

SFs in general now perform much better on the completely independent BDB2020+ data set, showing that the new split can help with better generalization. Furthermore, even though the models were trained using cocrystal structures, improvements are also seen on redocked structures, thus demonstrating the applicability for the retrained SFs in actual practice. Hence, we believe LP-PDBBind provides a better way of utilizing the existing PDBBind dataset, and provides a more realistic and meaningful benchmark to help develop higher quality and more generalizable SFs in the future.

## Methods

### Cleaning the PDBBind dataset

The majority of protein-ligand affinity prediction models are designed for non-peptide drug-like small molecules. However, a careful investigation of the protein-ligand complexes in PDBBind dataset elucidated that all the ligands are not necessarily drug like. Figure 1(a-c) shows the distributions of the number of heavy atoms, molecular weights and quantitative estimate of drug-likeness (QED)[43] for ligands in the general, refined and core set in the PDBBind dataset. Figure 1(d) shows that some ligands contain unusually large macrocycles, are peptide like, or contain long aliphatic chains. Including these data in the training of a ML model might make it harder for the model to learn key features related to the binding of drug-like small molecules to protein targets.

Some proteins and ligands in the PDBBind dataset contain uncommon elements, such as Hg, Cu and Sr in proteins or Co, Se or V in ligands; the proportion of occurrence for each element is summarized in Supplementary Table 3 for proteins and Supplementary Table 4 for ligands. A small portion of the structures in PDBBind dataset also contained steric clashes, and there was one structure for which the ligand was far away from any peptide chain (PDB ID: 2R1W). These should be excluded from the dataset due to the low quality of the complex structures, and the PDB IDs containing steric clashes (minimum heavy atom distance <1.75

Å) are also provided in the Supplementary Information. It is difficult for ML models to learn protein-ligand interactions when poor data and uncommon elements are present in the PDBBind dataset. Therefore, we have defined a "cleaner" version of PDBBind which only contains all data with ligand QED values larger than 0.2, protein and ligand elements that have at least 1‰ (more than 19 occurrences in the dataset), and minimum heavy-atom distance between protein and ligand fall within 1.75 and 4 Å. We call this subset of the data Clean Level 1 (CL1).

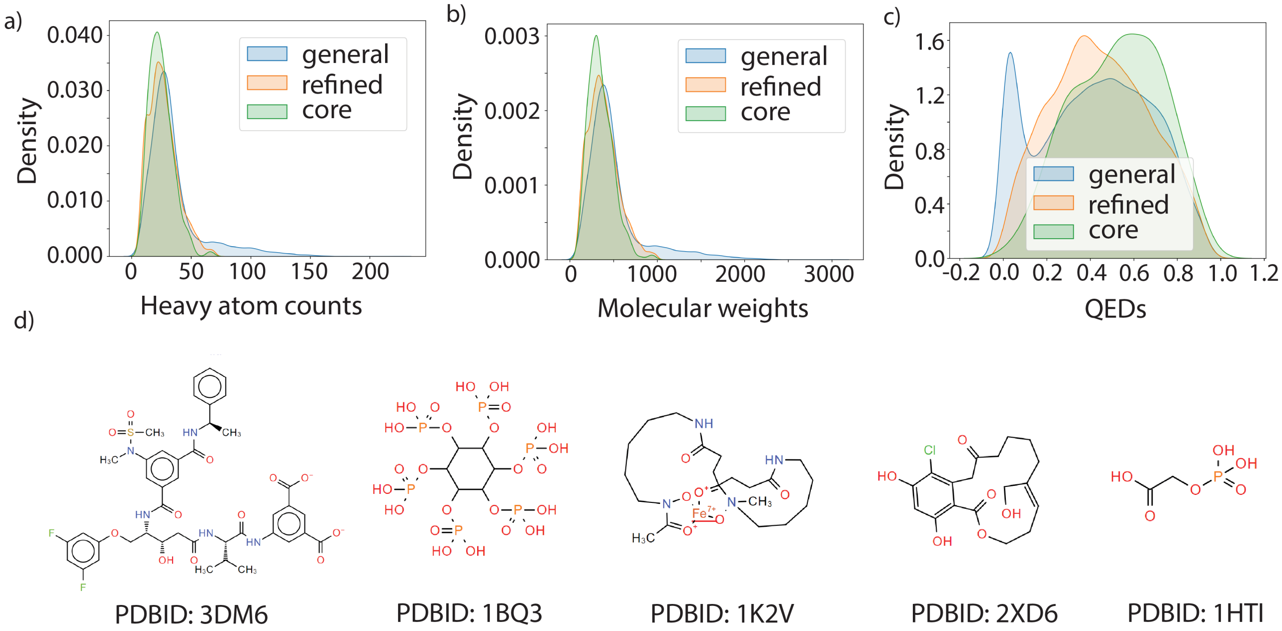

Figure 1: *Property distributions and example ligands in the PDBBind dataset.* (a) Distribution of number of heavy atoms, (b) molecular weights and (c) QED values for ligands in general, refined and core set of PDBBind dataset. (d) Example of ligand structures in the PDBBind dataset, with the corresponding PDB IDs

The binding affinity in terms of $\Delta G$ is directly related to the dissociation coefficient $K_d$ or $K_i$ through the formula $\Delta G = -RT\ln(K)$.[44] However, a large portion of the data in PDBBind is reported in terms of $IC_{50}$, which cannot be easily translated to $\Delta G$s due to its dependence on other experimental conditions and inhibition mechanisms.[45] The $IC_{50}$ values for the same protein-ligand complex can vary up to one order of magnitude in different assays. In addition, some data in PDBBind were not reported as exact values. Therefore, a second clean level (CL2) was defined on top of CL1, that additionally requires the target values are converted from records with "$K_d$ = xxx" or "$K_i$ = xxx", to ensure the reliability of experimental binding free energy data. Finally, considering the original splitting of PDBBind into general/refined/core was based on structure quality,[25] we also defined a third and highest quality level of data (CL3) that only retained data from refined and core set of PDBBind while still complying with all other quality-control metrics that we have defined. The total amount of data in PDBBind v2020 filtered with CL1, CL2 and CL3 are 14324, 7985 and 4404, respectively. The CL1 level dataset was used for retraining models described later because it contains the most amount of data. The CL2 level dataset was used for validation and test because the binding affinity data are more reliable at this level. We did not use CL3 in this study because it had the smallest amount of data, but as it may be useful for others we have provided identifiers of these protein-ligand complexes which are available at https://github.com/THGLab/LP-PDBBind/ along with the CL1, CL2 data sets.

### New splitting of PDBBind dataset

We have formulated a new splitting of the PDBBind dataset to minimize the similarity between training, validation and test data as much as possible in order to eliminate the risk of data leakage. Ligand similarities were calculated as the Dice similarity[46] between a pair of ligands based on Morgan fingerprints of the ligands using 1024 bits.[47] Similarities for proteins were calculated as the percentage of matched number of residues over the length of aligned sequences after sequences were aligned using the Needleman-Wunsch alignment.[48] More details on the calculation of similarity for proteins and ligands and data splitting procedure are given in the Supplementary Information. The total amount of data in the training, validation and test data after the new splitting are 11513, 2422 and 4860, respectively, and the data for each category is reported in Supplementary Table 5. The new splitting procedure ensures the data in the training set has a maximum protein sequence similarity of 0.5 and maximum ligand similarity of 0.99 to any data in the validation or test datasets, and the maximum similarity between any validation and test data is 0.9 for protein sequence similarity and 0.99 for ligand similarity. The similarity cutoffs are carefully chosen to reach a balance between the dis-similarity of train and test datasets and the amount of data in each subset. A higher similarity cutoff for the ligands was used compared to the protein similarity cutoff because similar ligands might interact differently with different types of proteins, which is useful for a ML model to learn.

Additionally, we explored the protein-ligand interaction similarities using proteo-chemometrics interaction fingerprints[49] and validated the exclusion of highly similar interaction patterns in the dataset using our splitting procedure. The proteo-chemometric interaction fingerprints extend the connectivity fingerprints (ECFPs) by the interactions between ligand atoms and nearby residues from the protein in 3D space, and maps the interaction patterns into a fixed size integer vector with a length of 256. The pairwise interaction fingerprint similarities were then calculated using a weighted Jaccard similarity score

$$S_{\mathrm{IFP}}(X, Y) = \frac{\sum_i(\min(X_i, Y_i))}{\sum_i(\max(X_i, Y_i))}$$

where $X$ and $Y$ are the interaction fingerprints for two complexes. As we will show later, our splitting procedure also ensures distinct separation of data in the train, validation and test subset of PDBBind in terms of interaction fingerprints.

### Compilation of the BDB2020+ dataset

Because many of the recent SFs have been trained on PDBBind, which means utilizing a subset of PDBBind as the test dataset risks data leakage, we have created a new benchmark dataset that is independent of PDBBind. To fulfill the need for a fair benchmark, we looked for the records deposited in BindingDB[40,50] after year 2020. Given that many data in BindingDB overlap with the PDBBind dataset which contains data up to year 2020, we have only looked at data in BindingDB deposited after year 2020 to minimize the chance of data leakage. BindingDB is one of the largest public binding affinity repositories, which also provides additional experimental conditions including assay information, pH and temperature. However, it does not guarantee each record has an associated 3D complex structure.

We have developed a workflow to match complex structures in RCSB PDB with records in BindingDB. Since a complex may contain multiple ligands, but only one matches the record in BindingDB, we selected the ligand in the PDB that has the best structural match with the SMILEs provided in BindingDB, and ensuring that the number of heavy atoms is exactly the same. We then used rdkit[51] to reassign bond orders to the extracted ligands using the BindingDB SMILES as reference. This step was necessary because bond orders are usually not present in a PDB file and are typically inferred from local atomic geometries, which sometimes result in unreasonable bonding structures; thus the bond order reassignment step ensures the rationality of the processed structures.

Additionally, any chain that is within 5 Å of the ligand was compared with the interacting chain sequence in BindingDB record. A reliable match was only made when the consecutive aligned residues are exactly the same. The reason to keep strict alignment criterion is that if the protein contains mutations, the binding affinity might change significantly, in which case the BindingDB record will not represent the true binding affinity for the complex structure in the PDB and is not usable for the benchmark. After discarding all unmatched data, we obtained 130 data records, out of which 115 contains accurate binding affinity data, and defines the BDB2020+ test dataset. The flowchart of the building process of BDB2020+ dataset is illustrated in Supplementary Figure S1.

We have further prepared the redocked structures using the original AutoDock Vina for each protein-ligand complex in the BDB2020+ dataset to evaluate the performance of the retrained SFs on docked poses instead of co-crystal structures obtained from experiments, because the co-crystal structures are usually unavailable in real-world applications. Specifically, for each data in BDB2020+ dataset, we run AutoDock Vina docking to generate 30

poses with exhausiveness=64. The pose with the lowest Vina score and the pose that is most similar to the cocrystal structure in terms of symmetry-corrected RMSD[52] were both retained. However, any data with the lowest RMSD to the co-crystal structure larger than 2 Å were excluded to prevent the influence of bad docking, resulting a total of 104 data remained for the redocked set of BDB2020+ that is closest to the co-crystal structure.

### Preparation of two target-specific datasets

Finally, we have prepared two additional datasets of protein-ligand complexes with the same protein and different ligands so that we could evaluate the ranking accuracies of the SFs before and after retraining using LP-PDBBind. The first dataset is based on the SARS-CoV-2 main protease (Mpro) for which a wide variety of potential Mpro inhibitors have been developed.[41] We have manually extracted published co-crystal structures of Mpro with a number of non-covalent inhibitors,[53–65] and prepared a dataset containing 40 structures and corresponding experimental binding affinity measurements. The second dataset involves the epidermal growth factor receptor (EGFR), which is a receptor tyrosine kinase related to multiple cancers including lung cancer, pancreatic cancer and breast cancer.[42] Similarly, we have selected 23 representative non-covalent protein-ligand complex structures of EGFR with binding affinities taken from BindingDB.[66–80] The two dataset were selected as two representative scenarios of using the SFs on a similar or different protein-ligand complex than what was included in the training dataset.

### Retraining Scoring Functions with LP-PDBBind

The LP-PDBBind CL1 cleaned data for non-covalent binders of PDBBind were used to retrain AutoDock Vina,[3] IGN,[15] the 2010 RF-Score[14] and DeepDTA.[18] These models cover a wide range of different approaches for scoring, and are representative of different dimensions of the SF space of models. AutoDock vina is a CSF that contains molecular interaction terms consisting of a van der Waals-like potential (defined by a combination of a repulsion term and two attractive Gaussians), a nondirectional hydrogen-bond term, a hydrophobic term, and a conformational entropy penalty, all of which are weighted by empirical parameters. The other three methods belong to MLSF category but vastly differ in their feature set. The RF-Score model is based on random forest regression that predicts binding affinities from the number of occurrences of a particular protein-ligand atom type pair interacting within a certain distance range using 3D structures. InteractionGraphNet (IGN) utilizes a graph neural network operating on the 3D complex structures, and the node and edge features are straightforward information about atoms and bonds, including atom types, atom hybridization, bond order, etc. Finally, DeepDTA is a Y-shaped 1-dimensional convolutional neural network that takes in protein sequences and ligand isomeric SMILES strings as input and outputs the predicted binding affinities, making it a model that does not rely on the exact 3D structure of the complex.

Details on the retraining of these models are described in the Supplementary Information. In short, AutoDock vina was retrained by optimizing its six empirical parameters by minimizing the mean absolute error between predicted and experimental binding affinities using the Nelder-Mead optimization algorithm.[81] Both IGN and DeepDTA were retrained by minimizing the mean squared error using gradient descent method as implemented in PyTorch.[82] For RF-Score, the optimization was driven by variance reduction between prediction and experimental affinities in each decision tree using the scikit-learn implementation.[83] After retraining, the new AutoDock Vina, RF-Score, IGN, and DeepDTA model performances are compared with the old models as tested on the non-covalent LP-PDBBind test set using the CL2 data, the BDB2020+ new benchmark data, the Mpro and EFGR applications, and finally evaluated on the redocked structures in BDB2020+ dataset.

## Results

### Analysis of PDBBind Splittings

Data distributions of PDBBind under the original split (general set/refined set/core set), Equibind Split (train/validation/test) and LP-PDBBind (train/validation/test) are provided in Figure 2 a-c. LP-PDBBind has significantly extended the size of the test set compared with the original PDBBind split and the more recent Equibind split. A bigger test set provides more accurate evaluation of model performance when the model is applied to data that has not been trained on. By contrast, the number of data in the training set is much smaller in LP-PDBBind. As we will show later, the shrinkage in the training set is necessary, because it keeps the similarity with validation/test set sufficiently low. Compared with the Equibind split, we have also decreased the number of discarded data, because we only discard data when they are highly similar to any other data in train, validation or test set.

One of the main purpose of defining the new split of PDBBind is to prevent data leakage between training, validation, and test data. To understand whether the new split has solved the data leakage issue, the maximum similarity for proteins and ligands between the training, validation, and test data under the EquiBind split and LP-PDBBind, and maximum similarity between the general set and core set under the original PDBBind split are summarized in Figure 2(d-g). The original PDBBind split has significant protein and ligand overlap between the general and core sets, as can be seen in the sharp peak at similarity of 1.0 in Figure 2(d,f). The same level of similarity between the refined and core sets in the original PDBBind split are shown in Supplementary Figure 2. Since many ML models use the PDBBind core set as the test dataset without carefully excluding similar data from their respective training dataset, the performance of these models reported may be overly optimistic and do not reflect their true generalizability and needs to be reevaluated.

The Equibind split is a significant step forward in reorganizing the PDBBind data more reasonably. Its time-based cutoff in defining the test dataset decreased the chance of data

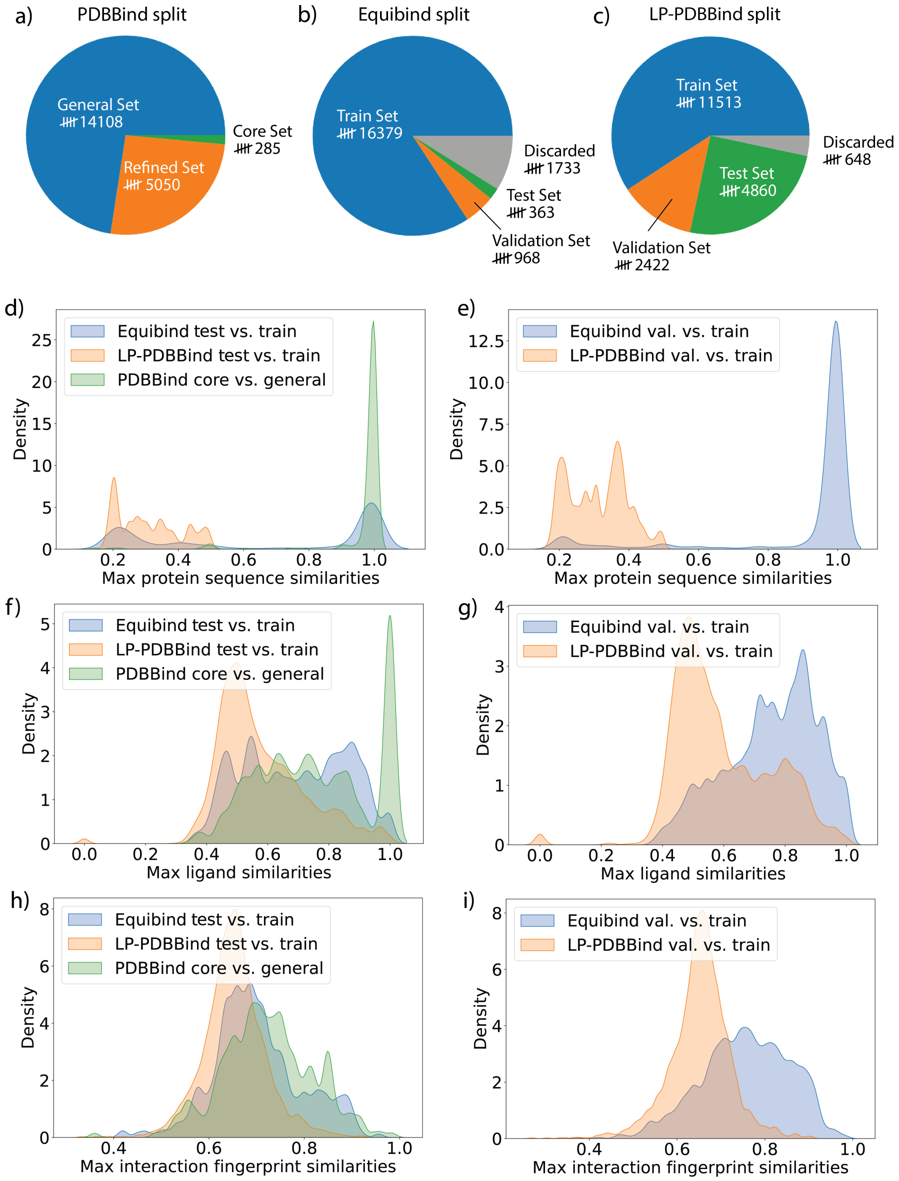

Figure 2: *Data statistics under different splits of the PDBBind dataset.* Number of data in different splits of the PDBBind dataset: (a) general, refined and core set in original PDBBind split; (b) train, validation, test set and discarded data in Equibind split; (c) train, validation, test set and discarded data in LP-PDBBind. Comparison of maximum protein sequence similarities between test set and train set (or core set and general set in original PDBBind split) (d) or between validation set and train set under Equibind split and LP-PDBBind(e). Comparison of maximum ligand similarities between test set and train set (or core set and general set in original PDBBind split) (f) or between validation set and train set under Equibind split and LP-PDBBind(g). Comparison of maximum interaction fingerprint similarities between test set and train set (or core set and general set in original PDBBind split) (h) or between validation set and train set under Equibind split and LP-PDBBind(i).

leakage, but still did not eliminate the possibility of highly-similar data that occurs in both train and test set. By comparison, LP-PDBBind minimizes overlap between data in the train and test set by design, keeping the maximum sequence similarty between any protein in the test set and any protein in the train set below 0.5, and the maximum ligand similarity below 0.99 (Figure 2(d,f)). Therefore, results for a model trained with the LP-PDBBind train set and evaluated with LP-PDBBind test set should better reflect the performance of the model when applied to a new protein-ligand complex that may be very different than data used for training the model.

Given that proteins with low sequence similarity might still be homologous and contain binding pockets that share similar interaction characteristics,[27] it is essential to double check on the similarities of protein-ligand interaction patterns for complexes in this new split of PDBBind dataset. The proteo-chemometrics based interaction fingerprints[49] were used to evaluate the interaction pattern similarities. Figure 2h shows the distribution of the maximum interaction fingerprint similarities for each complex in the test dataset with all complex structures in the training dataset under both the new LP-PDBBind split and the time-based Equibind split, and also the comparison between data in PDBBind core set and general set. LP-PDBBind has also eliminated data with high fingerprint similarity between train and test, which means the data with low protein sequence or ligand similarity but high interaction similarity is not an issue for the LP-PDBBind dataset, and the performance of any scoring function trained with LP-PDBBind train set and evaluated with the test set should be a realistic reflection of the model performance even when the protein-ligand interaction patterns are quite different from the complexes used for training.

To help with training more transferable models, we have also defined the validation set to be equally different than the train set as the test set. Figure 2(e,g,i) illustrates the maximum protein similarities, maximum ligand similarities and maximum interaction fingerprint similarities between the validation and training sets for the Equibind split and LP-PDBBind, respectively. The similarity between validation and training sets under LP-PDBBind is also well controlled so that the validation set can be used to select the most transferable model or hyperparameters when training the model. By comparison, the validation set in Equibind split is too similar to the train set, hence overfitting will not be effectively captured when monitoring model performance on the validation set. As is provided in Supplementary Figure 3, both the Equibind and LP-PDBBind splits of PDBBind, the validation and test set have a wide range of (dis)/similarities on proteins, ligands and their interactions. Therefore using the validation set for model selection will not lead to overfitting and thus increase transferability to the test set.

### Evaluating Retrained Models with LP-PDBBind

After the AutoDock Vina, IGN, RF-Score and DeepDTA models were retrained using LP-PDBBind, the performance were first compared on the LP-PDBBind test dataset filtered to CL2 for better reliability of data. Table 1 provides the mean and standard deviations on the root mean square error (RMSE) of the binding affinity prediction ($\Delta G_{bind}$) on the training, validation, and test data for the models retrained with LP-PDBBind from 3 different model initializations, and comparing it to the original models and their performance on the LP-PDBBind test data. Supplementary Figure 4 shows the scatter plots of the best model and reports the correlation coefficient between predicted and experimental binding affinities. Due to the data leakage issue, performance of the original models on the LP-PDBBind test data are over-estimated for the MLSFs. By comparison, AutoDock Vina due to its small number of trainable parameters does not suffer from the data leakage issue, and has achieved lower RMSE after retraining. Among the MLSF models, IGN has the smallest generalization gap, and is also the best performing model when evaluated using the LP-PDBBind test set within uncertainties. Since random forest models can almost perfectly fit training data,[84,85] the RF-Score model has exceptional performance on the LP-PDBBind training dataset, but its validation and test performance is on par with the IGN model. DeepDTA also performs quite well on the training dataset, but exhibits a large generalization gap with respect to the validation dataset, and has slightly higher RMSE on the test dataset than the other two MLSFs. Overall the original MLSF models have seen some of the LP-PDBBind test proteins and ligands in their training, and thus they appear to perform better than they actually do when data leakage is controlled for using the LP-PDBBind data.

Table 1: The training and test errors for the original and retrained models using LP-PDBBind for AutoDock Vina, IGN, RF-Score, and DeepDTA in terms of root mean square error (RMSE) in kcal/mol. Reported standard deviations were calculated with 3 different random initializations of model weights during training.

| **Model** | **RMSE Original** | **RMSE Retrained** | | |
|---|---|---|---|---|
| | test | train | validation | test |
| AutoDock Vina | 2.85 | 2.42 ± 0 | 2.29 ± 0 | 2.56 ± 0 |
| IGN | 1.82 | 1.65 ± 0.07 | 2.00± 0.03 | 2.16± 0.13 |
| RF-Score | 1.89 | 0.68 ± 0.003 | 2.14 ± 0.01 | 2.10 ± 0.003 |
| DeepDTA | 1.34 | 1.41± 0.11 | 2.07± 0.02 | 2.29± 0.04 |

Figure 3 and Table 2 summarize the scoring performance of the original and retrained models on the independent BDB2020+ benchmark dataset; the corresponding scatter plots for the original and retrained models are provided in Supplementary Figure 5. We see that upon retraining, all four models have achieved meaningful improvements on the BDB2020+ benchmark set. In terms of RMSE, AutoDock Vina decreased by 1.2 kcal/mol while the other three MLSFs decreased by 0.2-0.3 kcal/mol. The changes in the correlation coefficients are more profound: both IGN and RF-Score have achieved absolute correlation coefficients better than 0.5, and improvements for AutoDock Vina and DeepDTA are also clear. Overall

Table 2: Performance comparisons on the BDB2020+ benchmark set in terms of root mean square error (RMSE) and Pearson correlation coefficient ($R$) for different models before and after retraining using LP-PDBBind. Reported standard deviations were calculated with 3 different random initializations of model weights during training.

| **Model** | **RMSE (kcal/mol)** | | | ***R*** | |
|---|---|---|---|---|---|
| | original | retrained | difference | original | retrained |
| AutoDock Vina | 3.31 | 2.10 ± 0 | -37% | 0.23 | 0.29±0 |
| IGN | 1.62 | 1.38± 0.09 | -9∼20% | 0.41 | 0.54±0.04 |
| RF-Score | 1.80 | 1.61 ± 0.008 | -10∼11% | 0.36 | 0.51± 0.008 |
| DeepDTA | 1.98 | 1.72±0.10 | -9∼19% | 0.18 | 0.26±0.07 |

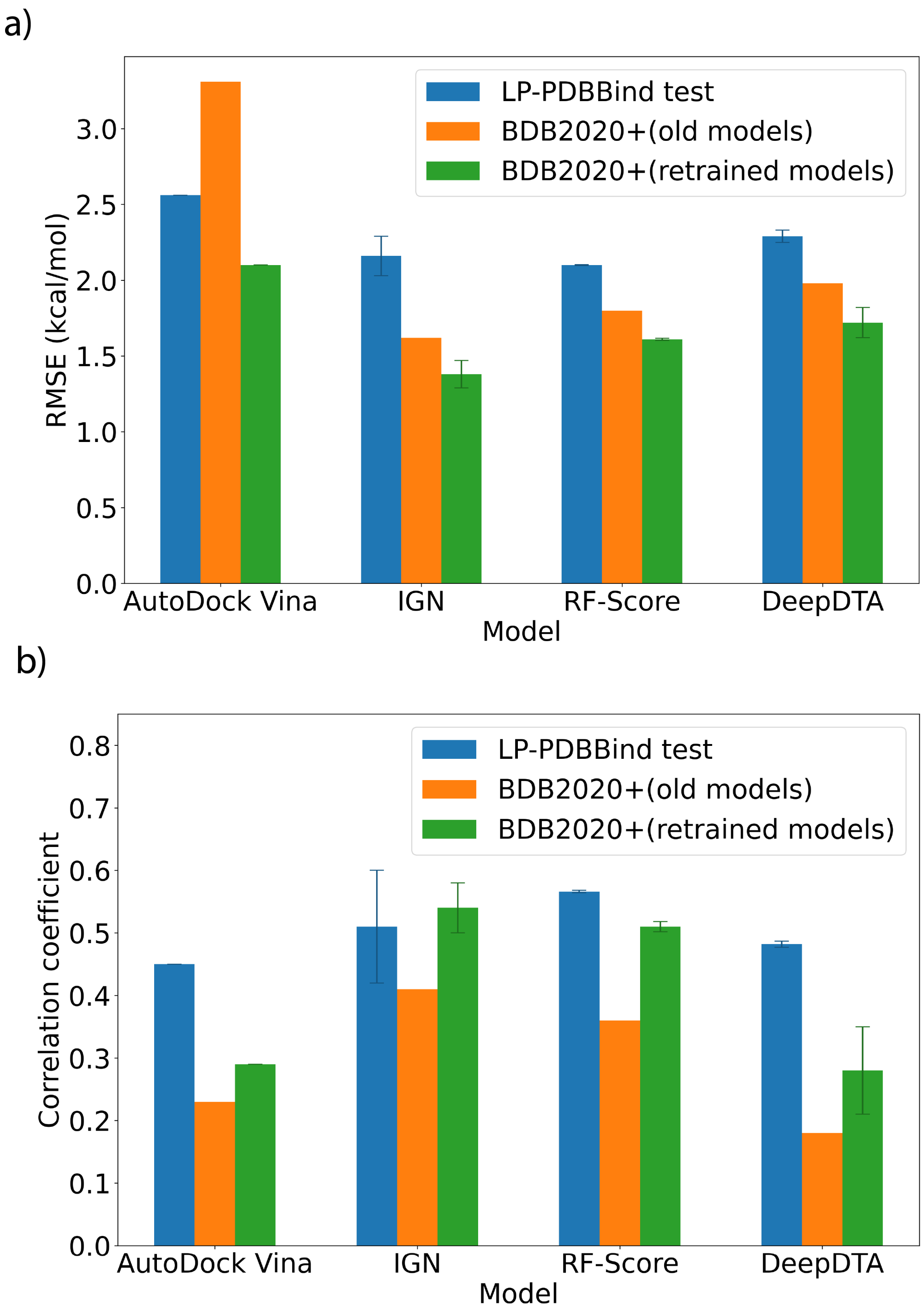

Figure 3: *Performance comparisons using different models and different benchmark datasets.* (a) Comparison on the root mean square error (RMSE) for different models. Lower is better. Blue bars indicate RMSEs on the LP-PDBBind test dataset using retrained models, orange bars indicate RMSEs for the models without retraining using LP-PDBBind, and green bars indicate RMSEs for the models retrained using LP-PDBBind. (b) Comparison on the Pearson correlation coefficient ($R$) for different models.

Overall, IGN and RFScore have improved more significantly than the other two, especially IGN that has achieved $1.38 \pm 0.09$ kcal/mol in RMSE and $0.54\pm 0.04$ in correlation coefficient when evaluated on the BDB2020+ dataset. We attribute the improvements on the model performances to our resplit of the PDBBind dataset because the training dataset becomes more diverse and representative, and that helps the SFs to find more transferable features and make more accurate predictions.

Figure 3 also compares the performance of the four models on BDB2020+ with the results evaluated on the LP-PDBBind test dataset. Interestingly, we find that all models have achieved lower RMSE on the BDB2020+ dataset but also lower Pearson correlation coefficients, $R$, other than IGN that has similar correlation coefficients between LP-PDBBind test dataset and BDB2020+ benchmark set. This seemingly contradictory result is actually reasonable due to the dataset distribution differences of the two evaluation benchmarks. The LP-PDBBind test dataset contains much more data than BDB2020+, and also spans a wider range of binding affinity values. The measured $-\log(K_d)$ values in the LP-PDBBind test dataset ranges from 0 to 12 (i.e. 12 orders of magnitude), but the BDB2020+ dataset only ranges from 4 to 10. Given that extreme predictions from a robust ML model is unlikely, a narrower range of binding affinities means the overall error will be smaller. However, it also poses challenge for successfully differentiating the nuances between more clustered data points, and therefore it is also more difficult to achieve higher correlation coefficients.

Nevertheless, we see that the relative rankings of the four methods are consistent between different evaluation benchmarks and different metrics. We find that IGN and RF-Score perform consistently better than AutoDock Vina and DeepDTA when trained on the new split. In terms of a generalization gap to new data, we also see the correlation differences are the smallest for IGN and RF-Score. These results are consistent with the overall performances of the models, and provide evidence that modern MLSFs can indeed surpass CSFs such as AutoDock Vina, even when protein and ligand similarities are low. While DeepDTA as a MLSF is an exception to this conclusion, we suggest that it is because it does not explicitly take molecular geometries as input, and it would perhaps explain why it does not achieve the same level of accuracy as MLSF or CSF models relying on 3-dimensional information of protein-ligand complexes.

### Evaluating the Ranking Capabilities of the Retrained Models

The improvement in scoring power, despite being significant, still does not fully reflect the ranking capabilities of the SFs which are more important in deciding which compound to prioritize in a real-world drug discovery campaign. Therefore, we have prepared an Mpro dataset and an EGFR dataset to evaluate the ranking accuracy of the SFs before and after retraining using LP-PDBBind.

Figure 4 shows the distributions of experimental binding affinities and protein and ligand similarities when comparing the LP-PDBBind training data with the Mpro and EGFR datasets. The binding affinities for these two systems are found to be in a narrower range than the LP-PDBBind training data. While the average binding affinity of the Mpro dataset is roughly in line with the LP-PDBBind training dataset, the ligands in the EGFR dataset have an overall tendency to bind stronger to their targets. This shift in the average indicates that the EGFR is more ”out-of-distribution” than the Mpro dataset. By calculating the Kullback-Leibler divergence (KL-divergence)[86] with the kernel density estimations between binding affinities in the LP-PDBBind train dataset and the two target-specific datasets, we found that the KL-divergence between LP-PDBBind train dataset and SARS-COV-2 Mpro dataset is 3.45, and the KL-divergence between LP-PDBBind train dataset and EGFR dataset is 4.46, also validating the EFGR dataset has more dissimilar binding affinities to the training dataset. Figure 4b-e further show that the protein and sequence similarities of the two evaluation datasets are overall very dissimilar to the LP-PDBBind training dataset, with the exception of a small fraction of protein and ligand sequence similarity attributable to the SARS-CoV-1 Mpro training entry PDB ID: 3V3M[87] for the Mpro dataset. Hence the Mpro and EFGR evaluations reflect two representative scenarios of using the SFs on a

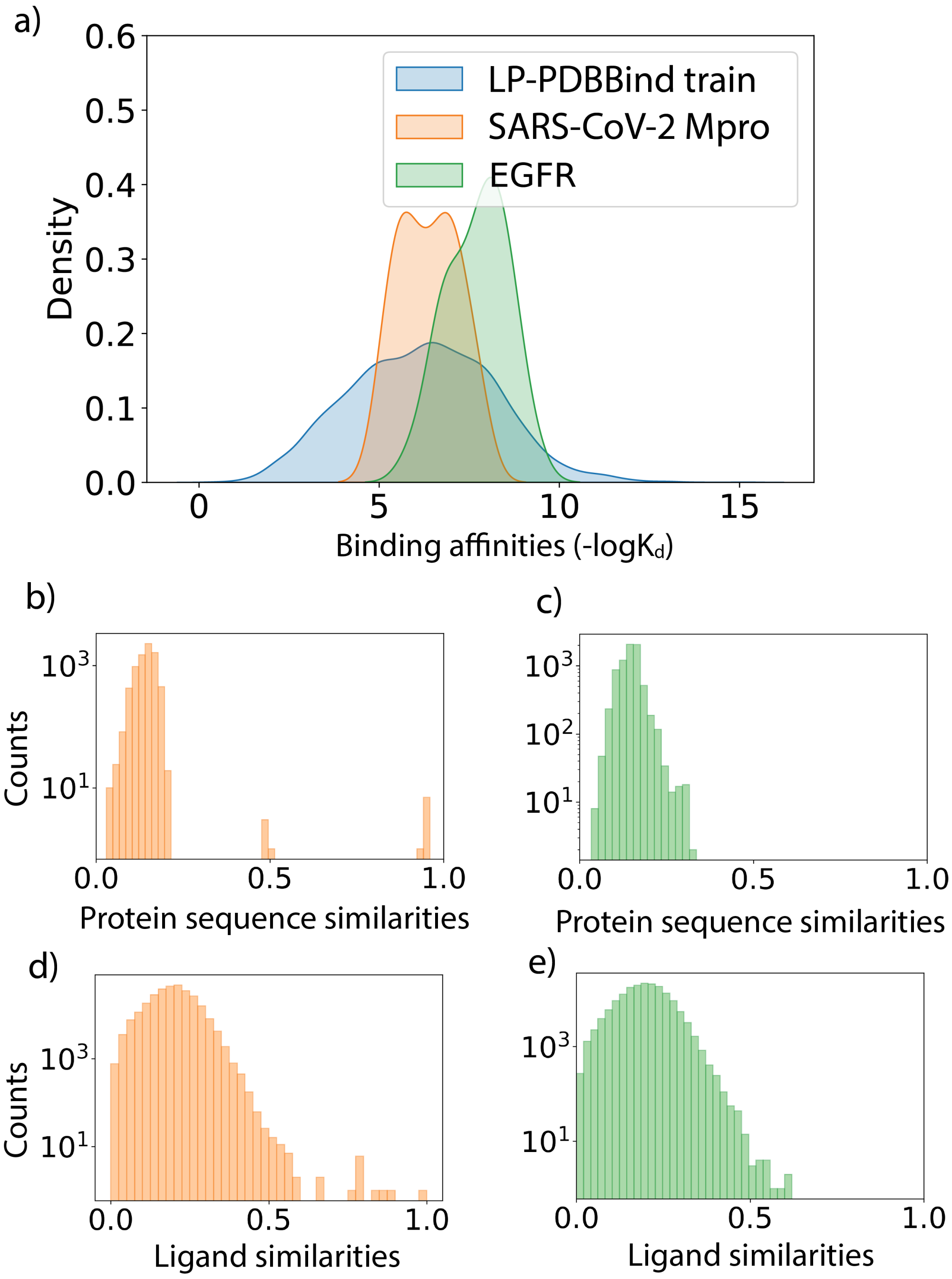

Figure 4: *Data statistics for the SARS-CoV-2 main protease (Mpro) benchmark set and epidermal growth factor receptor (EGFR) benchmark set.* (a) Distributions of the binding affinity data ($-\log K_d$) in the LP-PDBBind train dataset in blue, Mpro benchmark set in orange and EGFR set in green. (b-c) Distributions of protein sequence similarities between the Mpro protein (b) and EGFR protein (c) with proteins in the LP-PDBBind train dataset. (d-e) Distributions of ligand fingerprint similarities between molecules in the Mpro benchmark set (d) and EGFR benchmark set (e) with ligands in the LP-PDBBind train dataset.

similar or different protein-ligand system than what wasincluded in the PDBBind dataset.

Table 3 summarizes the RMSE, Pearson correlation coefficients ($R$) and Spearmann correlation coefficients ($R_S$ or ranking power) for the data in the Mpro and EGFR evaluation sets using both the original models and the models retrained using LP-PDBBind. The prediction scatter plots for the Mpro benchmark dataset and EGFR benchmark dataset are provided in Supplementary Figures 6 and 7. For the Mpro dataset, all four models exhibit some modest improvement in terms of RMSE. AutoDock Vina, RF-Score and DeepDTA have all achieved higher correlations in terms of $R$ and $R_S$. The correlation on the retrained IGN model has slightly decreased but is still similar to the original values. Taken all metrics into account, AutoDock Vina is the best performing SF for the Mpro dataset, although the differentiation among all models is not large.

Table 3: Performance comparisons on the SARS-CoV-2 main protease (Mpro) and epidermal growth factor receptor (EGFR) benchmark set in terms of root mean square error (RMSE) in kcal/mol, Pearson correlation coefficient ($R$) and Spearmann correlation coefficient ($R_S$) for different models before and after retraining using LP-PDBBind. The retrained model with the best performance on the BDB2020+ benchmark dataset was used to predict binding affinities in the Mpro and EGFR dataset.

| **Mpro Model** | **RMSE (kcal/mol)** | | $\boldsymbol{R}$ | | $\boldsymbol{R_S}$ | |
|---|---|---|---|---|---|---|
| | original | retrained | original | retrained | original | retrained |
| AutoDock Vina | 1.20 | 1.17 | 0.55 | 0.66 | 0.51 | 0.68 |
| IGN | 1.86 | 1.44 | 0.64 | 0.61 | 0.69 | 0.65 |
| RF-Score | 2.06 | 1.64 | 0.43 | 0.52 | 0.47 | 0.58 |
| DeepDTA | 1.18 | 0.88 | 0.59 | 0.64 | 0.60 | 0.65 |
| **EFGR Model** | **RMSE (kcal/mol)** | | $\boldsymbol{R}$ | | $\boldsymbol{R_S}$ | |
| | original | retrained | original | retrained | original | retrained |
| AutoDock Vina | 3.11 | 1.59 | 0.25 | 0.38 | 0.21 | 0.36 |
| IGN | 1.06 | 0.96 | 0.36 | 0.65 | 0.17 | 0.62 |
| RF-Score | 1.57 | 0.97 | -0.15 | 0.52 | -0.18 | 0.45 |
| DeepDTA | 1.22 | 1.05 | 0.23 | 0.44 | 0.20 | 0.43 |

However, the results on the EGFR dataset exhibit much larger variations for the different models, and the changes are significant for all the SFs. There is a dramatic decrease by ~50% in RMSE for AutoDock Vina, and both $R$ and $R_S$ have increased significantly. IGN has a slight decrease in RMSE but the correlation coefficients have improved remarkably from 0.36 to 0.65 in Pearson correlation coefficient and from 0.17 to 0.62 in in Spearmann correlation coefficient. These values are the highest among all retrained models, and do not differ much with the Mpro results and thus showing good generalizability. RF-Score has also benefitted from retraining quite significantly, with a 0.6 kcal/mol decrease in RMSE, and the correlation coefficients have improved from -0.15 to 0.52 in terms of $R$ and from -0.18 to 0.45 in terms of $R_S$. Finally, DeepDTA has also seen improvements upon retraining, but the ranking capability of the retrained DeepDTA model is still inferior to the other two MLSFs. Overall, the performance rankings of the four SFs are still consistent with that obtained from the LP-PDBBind test set benchmark and the BDB 2020+ benchmark. But the EFGR benchmark emphasizes that new applications will benefit from better generalizability of the newly retrained models, especially for IGN.

### Rescoring Docked Poses and Pose Selection using the Retrained Scoring Functions

Cocrystal structures are usually unavailable during the early stages of the drug discovery process. This it is important to quantify the binding affinity estimations from the retrained SFs using predicted structures, such as the complex structures obtained from molecular docking. Using the BDB2020+ structures redocked with AutoDock Vina and the original Vina scoring function, we have evaluated the accuracies of the retrained AutoDock Vina, IGN and RF-Score as a rescoring method, and the results are summarized in Table 4; DeepDTA was not compared because its predictions do not rely on the exact 3-dimensional structures of the complex.

Following a recent study that suggests a modified set of empirical parameters for AutoDock Vina that improves its ranking capabilities, we have also evaluated its performance on the redocked BDB2020+ benchmark set.[88] To better separate pose selection capabilities from scoring capabilities, we report metrics on both the redocked structures with the best docking scores, and the redocked poses that have the lowest RMSDs to the cocrystal structures.

Table 4: Performance comparisons on scoring with redocked structures of the BDB2020+ benchmark set in terms of root mean square error (RMSE) and Pearson correlation coefficient (R) for different models before and after retraining using LP-PDBBind. The retrained model with the best performance on the BDB2020+ benchmark dataset was used in this benchmark. AutoDock Vina with the newly suggested weights in Ref. 88 was also added for comparison.

| **Redocked Structures (Best Score)** | **RMSE (kcal/mol)** | | ***R*** | |
|---|---|---|---|---|
| | original | retrained | original | retrained |
| AutoDock Vina | 2.12 | 1.99 | 0.22 | 0.26 |
| AutoDock Vina with Modified Weights (Ref. 88) | 3.35 | / | 0.25 | / |
| RF-Score | 1.78 | 1.56 | 0.34 | 0.49 |
| IGN | 1.66 | 1.42 | 0.33 | 0.46 |
| **Redocked Structures (Closest to Cocrystal)** | **RMSE (kcal/mol)** | | ***R*** | |
| | original | retrained | original | retrained |
| AutoDock Vina | 2.18 | 2.02 | 0.13 | 0.22 |
| AutoDock Vina with Modified Weights (Ref. 88) | 3.40 | / | 0.17 | / |
| RF-Score | 1.83 | 1.61 | 0.27 | 0.46 |
| IGN | 1.65 | 1.43 | 0.34 | 0.43 |

Again, we observed a consistent improvement over the original models after retraining the SFs using LP-PDBBind, which means even though the models were trained using cocrystal structures, they could be used to rescore docked poses and achieve higher accuracies when compared with experimental binding affinities. In the top-scored redocked structure in BDB2020+ benchmark set, AutoDock Vina has improved from 2.12 to 1.99 kcal/mol in RMSE and the correlation coefficients improved from 0.22 to 0.26. Compared to the suggested new weights in Ref. 88, the AutoDock Vina scoring function retrained with LP-PDBBind still has better scoring and ranking capabilities. The improvements on the retrained RF-Score and IGN models are also distinct, especially in the correlation coefficients that have improved from 0.34 to 0.49 for RF-Score and from 0.33 to 0.46 for IGN. The performance on the redocked structures that are closest to the known cocrystal structure eliminates the influence of bad docked poses and purely reflects the scoring and ranking capabilities of the retrained models on correctly docked structures. The performance was similar to that evaluated on the best-scored poses, further showing that the improvements obtained by retraining the models using cocrystal structures are transferable to docked structures.

However, we would like to emphasize that the newly trained Vina scoring function is not intended for docking, because a scoring function designed for docking should be trained with high energy decoy poses which are missing in the PDBBind dataset. Consequently, the repulsion parameter in our retrained Vina scoring function (0.014212) is much smaller than the original value (0.840) and the scoring function cannot differentiate bad poses that have atoms too close to each other. Nevertheless, the improvements shown for scoring power and ranking power are meaningful, because the virtual screening process can be broken into multiple steps, and we could use the original Vina SF for docking and the retrained one to rescore top poses to achieve better accuracy.

## Discussion and Conclusion

The area of computational drug discovery relies on generalizable scoring functions that have robust scoring and ranking power of binding affinities of ligand-protein complexes. However due to the lack of independently built datasets that test the true generalizability of the SFs, it is hard to differentiate among the plethora of many models which have been trained and tested on the original split of the PDBBind dataset. As we have shown, there is too much overlap between the PDBBind general and refined data used for training with the core subset, leading to the possibility of inflated performance metrics that in turn lead to false confidence in how such models will perform on new protein-ligand complexes.[89]

In order to reduce data similarity between training, validation, and test data of the PDBBind dataset, we have developed LP-PDBBind using an iterative process to select most similar data first into the test set, and then validation set, so that the final training dataset has low similarity with validation or test dataset. We have also cleaned the PDBBind data in multiple ways: CL1 removed covalent ligand-protein complexes, the low populations of drug molecules with underrepresented chemical elements, and complexes with steric clashes. In addition to CL1, the CL2 level of cleaning aimed for consistent measures of binding free energies by either converting $K_d$ or eliminating data reported as $IC_{50}$. Finally, CL3 eliminated the general set to perform splits on the refined and core set data which is deemed of higher quality. However, it is always a trade-off between the quality of data and the amount of data, and all of the results reported here were based on training on CL1 data and testing on CL2. In addition we have isolated the covalent binding data from PDBBind that may help newly formulated SF predict these type of protein-ligand complexes.

Furthermore, to provide a benchmark dataset truly independent of PDBBind, we have compiled the BDB2020+ dataset derived from the BindingDB database deposited after 2020 to minimize time-cutoff based data leakage, and further ensuring that there is no overlap with PDBBind. Additionally, we also tested ranking power through construction of the Mpro and EFGR ligand-protein complex series. The SARS-CoV-2 Mpro protein has high similarity counterparts in the PDBBind dataset, and the other series involving EGFR does not have anything similar in the training dataset of PDBBind. These new data should also be useful in future evaluation and/or finetuning of any scoring function.

In this work we utilized the new split of PDBBind using CL1 to retrain AutoDock Vina, IGN, RF-Score and DeepDTA and compared the old models with the retrained models on the LP-PDBBind test set, as well as the fully independent BDB2020+ data, Mpro series, and EGFR series. We have demonstrated that using a different splitting of the same dataset leads to significant performance improvements, and the improvements are transferable from using co-crystal structures as input to using docked structures as input. The comparison between the different benchmark results for the SFs can be well explained by the "difficulty level" of these datasets, and provide insights about the generalizability of various models. We found that well performing models also have more stable ranking results for different ligands towards a protein target. When the target system is similar to data included in the training dataset, the differentiation between models are not obvious. However, when the protein target is not similar to anything in the training dataset, we found that different SFs demonstrate quite different generalization capabilities; the IGN model retrained with

LP-PDBBind is recommended due to its reliable good scoring and ranking power.

However, since the PDBBind dataset does not contain decoy structures that are important to allow the models to learn characteristics of true complex structures, the retrained SFs are not expected to have better docking power for recognizing native ligand bound poses. The usefulness of the retrained SF relies on their capability to rescore docked poses and achieve better accuracy when compared to experimental binding affinity measurements. Meanwhile, it would be worthwhile to generate decoy structures for the BDB2020+ dataset to better benchmark the docking and screening powers of the SFs independent of the PDBBind dataset. In summary, the cleaned LP-PDBBind data in its current form is a valuable resource for training more transferable SFs, and we hope reporting evaluation metrics on the BDB2020+ dataset can also become a common practice for future SFs.

### Acknowledgement

We fulsomely acknowledge Charles Brooks for his distinguished career and sustained excellence in biophysics. We sincerely thank Prof. Michael Gilson for assistance with the BDBind data set. This work was supported by National Institute of Allergy and Infectious Disease grant U19-AI171954. This research used computational resources of the National Energy Research Scientific Computing, a DOE Office of Science User Facility supported by the Office of Science of the U.S. Department of Energy under Contract No. DE-AC02-05CH11231.

### Availability and Implementation

All the code for creating LP-PDBBind dataset, building the BDB2020+ dataset, the prepared dataset files in the .csv format, and python scripts for retraining AutoDock Vina, IGN, RFScore and DeepDTA models are provided in a public accessible GitHub repository: https://github.com/THGLab/LP-PDBBind

### Supporting Information Available

Computational procedures and characterization of the PDBBind, EquiBind, and LP-PDBBind data sets, cleaning procedures, and results for original and retrained scoring functions.

## Supporting Information
# Leak Proof PDBBind: A Reorganized Dataset of Protein-Ligand Complexes for More Generalizable Binding Affinity Prediction

Jie Li,[†] Xingyi Guan,[†] Oufan Zhang,[†] Kunyang Sun,[†] Yingze Wang,[†] Dorian Bagni,[†] and Teresa Head-Gordon[*,†]

† *Pitzer Center for Theoretical Chemistry, Department of Chemistry, University of California, Berkeley CA 94720, USA*

‡*Departments of Bioengineering and Chemical and Biomolecular Engineering, University of California, Berkeley, CA, USA*

E-mail: thg@berkeley.edu

### Reformulation of the PDBBind Dataset

#### Description for processing the PDBBind dataset

All PDB files were downloaded from RCSB to recover the original headers, and the categories of the proteins were defined according to whether the keywords occurred in the header. The header information was extracted using the biopython package version 1.81.[?] The categories we considered include transport proteins, hydrolases, transferases, transcription proteins, lyases, oxidoreductases, isomerases, ligases, membrane proteins, viral proteins, chaperone proteins, and metalloproteins. A protein file with none of these keywords occurring in the header were categorized into a generic "other" category. Protein sequences were extracted directly from the PDBBind dataset files using the SEQRES records, and sequences for proteins with multiple chains were concatenated using a column (:) symbol. Additionally, the SMILES strings for each of the ligands were extracted using the rdkit package (2023.3.2 version)[?] from either the .mol2 or .sdf file provided in the original PDBBind dataset. The .mol2 files were used with higher priority due to their generally better description of bond orders in the ligands compared with the .sdf files in the PDBBind dataset. The latter were used instead when rdkit failed to read in .mol2 files.

### Similarity calculation and Data Resplitting of protein and ligands in the PDBBind dataset

Similarities for each ligand to any other ligand in the whole PDBBind dataset were calculated based on the Morgan fingerprints of the ligands using 1024 bits.[?] According to a study that compares multiple similarity score metrics, the Tanimoto index, Dice index, Cosine coefficient and Soergel distance were giving similar results on molecule similarity calculations and all worked pretty well.[?] In our work the Dice similarity is reported for the ligand pairs according to the following:[?]

$$sim = \frac{2|A \cup B|}{|A| + |B|} \tag{1}$$

where $|A \cup B|$ counts the number of bits set to ON in the fingerprints of both ligands A and B, and $|X|$ counts the number of bits set to ON in an single ligand X. A radius of 2 was first used when calculating Morgan fingerprints of the ligands; if the similarity between two ligands was calculated to be 1, it is recalculated using Morgan fingerprints of the ligands with radius of 4 to allow more careful validation of ligand identity, and the radius is extended to 10 if a radius of 4 still result in a similarity of 1. If the similarity was still 1 with the extended radius for calculating the fingerprint, the canonical SMILES string of the two molecules were compared and any discrepancy in the canonical SMILES will enforce the similarity to 0.99.

These steps were designed to ensure ligands with similarity of 1 are strictly identical.

Similarities for proteins were calculated based on the aligned sequences of the proteins. Considering that it is unlikely two proteins belonging to different functional categories are similar, the sequence similarities were calculated only for proteins in the same category (i.e. transport proteins, hydrolases, etc.), and any two proteins that belong to different categories were defined to have similarity of 0. Within each category, every pair of protein sequences were aligned using the Needleman-Wunsch alignment algorithm,[?] and the similarity was calculated as the number of aligned residues divided by the total length of the aligned sequence.

Data splitting was first done inside each protein category, and an iterative process was employed to separate the dataset step by step using the following algorithm: In the first iteration, 5 complexes were randomly selected as seed data in the test set, and all data in the same protein category that have protein sequence similarity greater than 0.9 or ligand similarity greater than 0.99 were added to the test set as well. If protein-ligand complex data that have protein sequence similarity greater than 0.5 or ligand similarity greater than 0.99 were found, they too were added to the validation set. Any data newly added to the test set in this iteration will become seed data in the next iteration, and the iteration continues until no new data are added to the test set. Next, a similar process was applied to the validation data, adding all remaining data that have protein sequence similarity greater than 0.5 or ligand similarity greater than 0.99 into the validation set. The remaining data is then defined as the training set.

After data splitting by protein category, the protein-ligand complexes for training, validation, and testing were combined to define the splitting for the whole dataset. However, after combination the ligand similarity might still exceed 0.99 for data from different categories. Therefore, any data in the combined training set that has ligand similarity greater than 0.99 to any other data in the validation or test set were discarded altogether. The resulting number of data in the training, validation, and test set were 11513, 2422 and 4860, respectively, after this cleaning step.

#### Covalent Binders in PDBBind v2020

The PDBBind v2020 dataset includes 14108 protein-ligand complexes in the general set, 5050 complexes in the refined set and 285 complexes in the core set.[?] It is important to treat covalent and non-covalent binders separately in PDBBind, because most existing algorithms that predict protein-ligand binding primarily focus on non-covalent interactions. As far as we are aware, there has been no systematic study of whether the binders in PDBBind are covalent or non-covalent. Relying on the CovBinderInPDB[?] repository, we have identified covalent binders in the PDBBind dataset. The ligand names were extracted from the PDB files by comparing the minimum distance between any atom from the ligands in PDBBind database and residues in the PDB files downloaded from the RCSB database. If the minimum distance is less than 1Å, the matched residue name was compared with the record in CovBinderInPDB to identify whether the ligand is a covalent binder. If the minimum distance is more than 1Å or the residue name did not match the record in CovBinderInPDB, the structures were manually checked to identify whether the ligands are covalent binders or not. Ultimately 893 covalent binders and 18550 non-covalent binders were identified in the PDBBind dataset.

### Model Retraining Procedures

All models were retrained using the CL1 version of the LP-PDBBind training dataset to achieve balance between data cleanliness and the amount of training data available. For model validation and testing, the non-covalent LP-PDBBind validation and test data at CL2 were used to ensure data quality is higher, and can be considered as an optimistic estimation of the performance of these models on high quality data.

### AutoDock Vina Retraining

There are six empirical parameters in the AutoDock Vina scoring function (gauss1, gauss2, repulsion, hydrophobic, hydrogen, and rotation penalty).[?,?] For each molecule in the LP-PDBBind training set, the 6 individual terms of AutoDock Vina were calculated using the vina binary (v1.2.3) by setting weight of one term to one and the rest of the weight to 0. The weights of these six terms were optimized by minimizing the mean absolute error between the weighted sum of six terms and binding free energy through the Nelder-Mead optimization algorithm.[?] The final retrained AutoDock Vina parameters are provided in Supplementary Table 1 and all further evaluations of retrained models are done with these modified weights.

Table 1: Retrained AutoDock Vina Weights

| **Term** | **Weight** |
|---|---|
| gauss1 | 0.003372 |
| gauss2 | -0.008098 |
| repulsion | 0.014212 |
| hydrophobic | -0.008361 |
| hydrogen | -0.227928 |
| rot | 0.05846 |

### RF-Score Retraining

The RF-Score (RF) uses Random Forest model to predicts binding affinities with counts of a particular protein-ligand atom type pair interacting within a certain distance range based on 3D structures.[?] We here followed the RF-Score-v1 approach, where nine common elemental atom types (C, N, O, F, P, S, Cl, Br, I) for both the protein and the ligand were considered and neighboring contacts between a protein-ligand atom pair were defined within 12 Å. Only atom pairs with non-zero occurrence over the whole training dataset were considered, which result in 36 features in total. For fairness of comparison, we trained a RF model on the PDBbind2007 refined set, denoted as the original model, and on the LP-PDBBind training data, as the retrained model, using the same script.

### IGN Retraining

InteractionGraphNet (IGN)[?] utilizes two independent graph convolution modules to sequentially learn the intramolecular and intermolecular interactions from the 3D structures of protein-ligand complexes to predict binding affinities.[?] Protein pockets were extracted using Chimera (v1.17.3) by selecting residues that are within 10 Å of any atom from the ligand, and then the protein and ligand structures were saved as RDKit objects. Due to molecule generation errors in RDKit,[?] which is required for featurizing the 3D structures into graph representations, only 6378 complexes from the LP-PDBBind training set were used. The code for retraining the IGN model (https://github.com/THGLab/LP-PDBBind/tree/master/model_retraining/IGN) was adapted from the original training scripts using the same feature size and layer numbers as the original published model with only the training data modified. The exact hyperparameters for retraining are provided below:

Table 2: Retrained IGN hyperparameters

| **Term** | **Weight** |
|---|---|
| epochs | 500 |
| batch_size | 128 |
| graph_feat_size | 128 |
| num_layers | 2 |
| outdim_g3 | 128 |
| d_FC_layer | 128 |
| repetitions | 3 |
| lr | 0.001 |
| l2 | 0.00001 |
| dropout | 0.2 |

### DeepDTA Retraining

DeepDTA is a purely data-driven approach which does not rely on physical interactions or 3D structural information. Instead, it uses 1D convolutions on the string representations of the proteins and ligands to make predictions.[?] Since the method did not originally train on the PDBBind dataset, we retrained the model with the original PDBBind general and refined sets using a PyTorch implementation of the original code: https://github.com/THGLab/LP-PDBBind/tree/master/model_retraining/deepdta. More specifically, we did a 90-10 train-validation split on the data for training and then tested its performance on the core set. Different protein and ligand kernel sizes were used as suggested by the original work and the best-performed parameters with the protein kernel size of 12, ligand kernel size of 4 and channel size of 32 were chosen to serve as the original model in our performance comparison tables. The retrained model with LP-PDBBind was also generated following the same scheme.

Since the 1d convolution requires a fixed size of the protein sequence and ligands, during all training processes, proteins with sequence lengths longer than 2000 and ligands with isomeric SMILES lengths longer than 200 were discarded, resulting in the final number of training data, validation data and test data to be 7338, 956 and 2162, respectively. Also, since the ligand encoding was based on the training set only, all ligands with unseen tokens from their isomeric SMILES strings in the test set and real-world examples are discarded, resulting in a loss of 15 data points. It is worth noting that these losses of data happen to both the original model and the retrained model, so it won't affect our conclusions in this work.

## Supporting Tables

Table 3: Counts and proportion of chemical element occurrences in all proteins in the PDB-Bind dataset

| Element | Number of data containing element | Proportion of data containing element |
|---|---|---|
| C | 19398 | 1.00 |
| S | 16375 | 0.84 |
| O | 19398 | 1.00 |
| N | 19398 | 1.00 |
| Zn | 1842 | 0.09 |
| Ca | 400 | 0.02 |
| Co | 66 | 0.003 |
| Mg | 858 | 0.04 |
| Ni | 79 | 0.004 |
| Mn | 356 | 0.02 |
| Fe | 104 | 0.01 |
| Na | 138 | 0.01 |
| K | 78 | 0.004 |
| Se | 107 | 0.01 |
| Hg | 6 | 0.000 |
| Cu | 16 | 0.001 |
| Cs | 1 | 0.000 |
| P | 105 | 0.01 |
| Cd | 22 | 0.001 |
| Sr | 1 | 0.000 |
| Ga | 1 | 0.000 |
| Rb | 1 | 0.000 |

Table 4: Counts and proportion of chemical element occurrences in all ligands in the PDB-Bind dataset. Only elements that occur in the binding pocket were considered.

| **Element** | **Number of data containing element** | **Proportion of data containing element** |
|---|---|---|
| C | 19398 | 1.00 |
| S | 5146 | 0.27 |
| O | 18338 | 0.95 |
| N | 17515 | 0.90 |
| Cl | 2255 | 0.12 |
| P | 2087 | 0.11 |
| F | 2500 | 0.13 |
| Br | 459 | 0.02 |
| I | 133 | 0.01 |
| B | 240 | 0.01 |
| Se | 16 | 0.001 |
| Fe | 21 | 0.001 |
| Co | 4 | 0.000 |
| V | 2 | 0.000 |
| Ru | 10 | 0.000 |
| Be | 1 | 0.000 |
| Ir | 5 | 0.000 |
| Zn | 1 | 0.000 |
| Mg | 1 | 0.000 |
| Re | 1 | 0.000 |
| As | 4 | 0.000 |
| H | 6 | 0.000 |
| Cu | 1 | 0.000 |
| Pt | 2 | 0.000 |
| Rh | 1 | 0.000 |
| Si | 5 | 0.000 |
| Sb | 1 | 0.000 |

Table 5: Number of train, validation and test data in each protein category before merging for the new split of PDBBind

| Protein type | Train | Validation | Test |
|---|---|---|---|
| hydrolase | 4038 | 318 | 1377 |
| transferase | 2226 | 1228 | 1837 |
| other | 3050 | 405 | 134 |
| transcription | 642 | 52 | 298 |
| lyase | 331 | 71 | 465 |
| transport | 503 | 35 | 83 |
| oxidoreductase | 342 | 52 | 182 |
| ligase | 342 | 38 | 90 |
| isomerase | 199 | 70 | 64 |
| chaperone | 50 | 66 | 184 |
| membrane | 157 | 56 | 71 |
| viral | 196 | 18 | 53 |
| metal containing | 85 | 13 | 22 |

## Supporting Figures

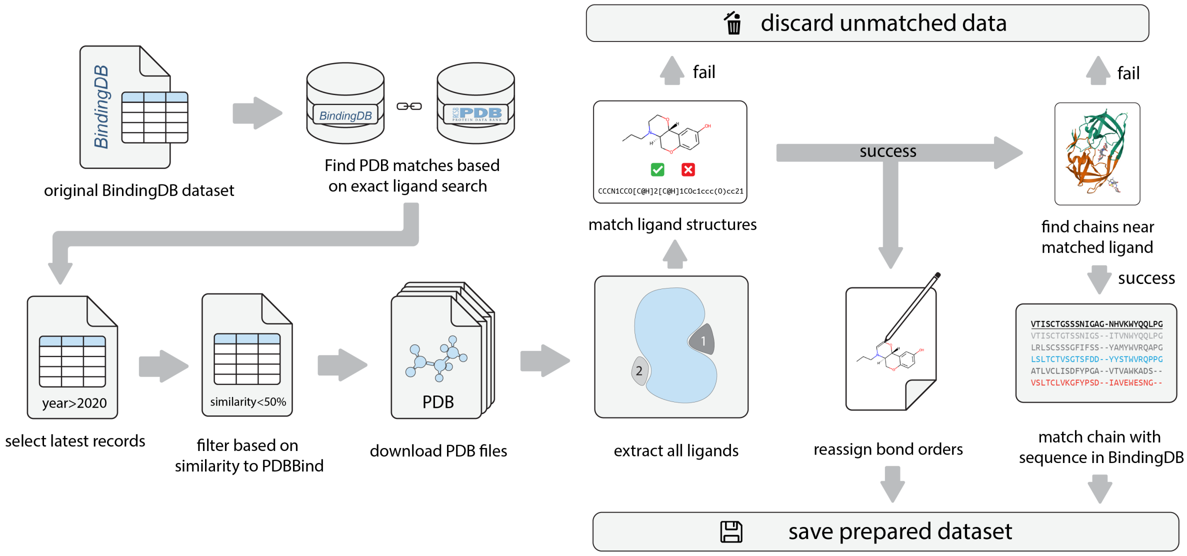

Figure 1: *Flowchart of the BDB2020+ curation process.* Starting with the original BindingDB dataset, the records with potential matched structures in the RCSB database were identified by searching the InChi keys that occur in BindingDB to find all PDB records that contain the same ligand, and then further filtering based on the PDBID of the target chain that was also recorded in the BindingDB dataset. Release dates of the PDB structures were extracted from the RCSB PDB records and only records after year 2020 were selected for further processing. Additionally, we employed the same similarity criterion as what was used in developing LP-PDBBind, and removed all data that has sequence similarity greater than 50% or ligand similarity greater than 99%. The filtered PDB files were downloaded from the RCSB database and small molecule ligands were extracted from the PDB files. The original BindingDB dataset[?,?] was matched with PDB database records by exact ligand matches. The matched records were further filtered based on release date and similarity with the PDBBind dataset. All ligands were extracted from the downloaded PDB files, and the ligand matching the SMILES in the BindingDB dataset was selected for further process. Protein chains in the vicinity of the ligand were also extracted. Both the matched ligand and protein chains were further processed to ensure identity with the record in PDBBind. Any discrepancy will result in a mismatch of data and will be discarded.

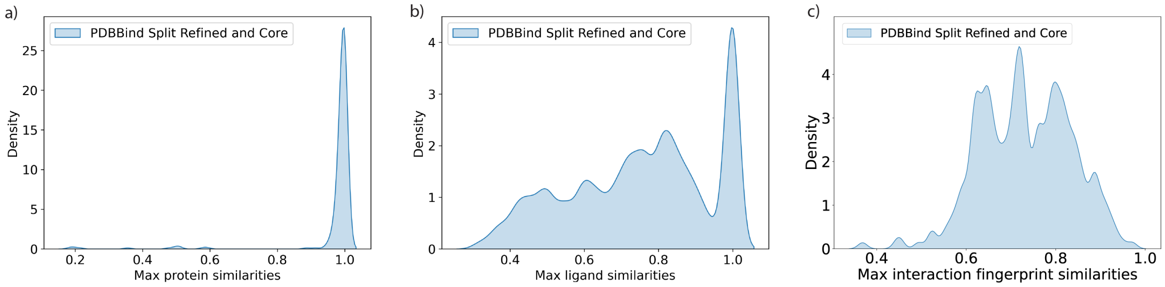

Figure 2: *Data similarity between refined set and core set in the PDBBind original split.* Comparison of maximum protein similarities (a), ligand similarities (b) and interaction fingerprint similarities (c) between refined set and core set under original split of PDBBind.

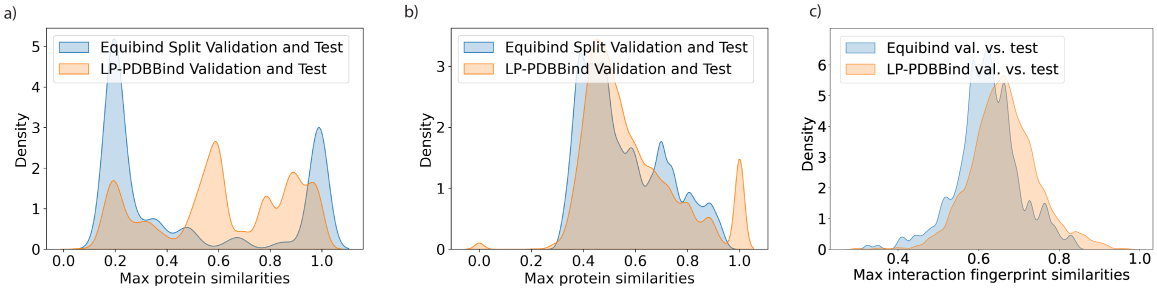

Figure 3: *Data similarity between validation set and test set in different splittings of PDB-Bind.* Comparison of maximum protein similarities (a), ligand similarities (b) and interaction fingerprint similarities (c) between validation set and test set under Equibind split and LP-PDBBind.

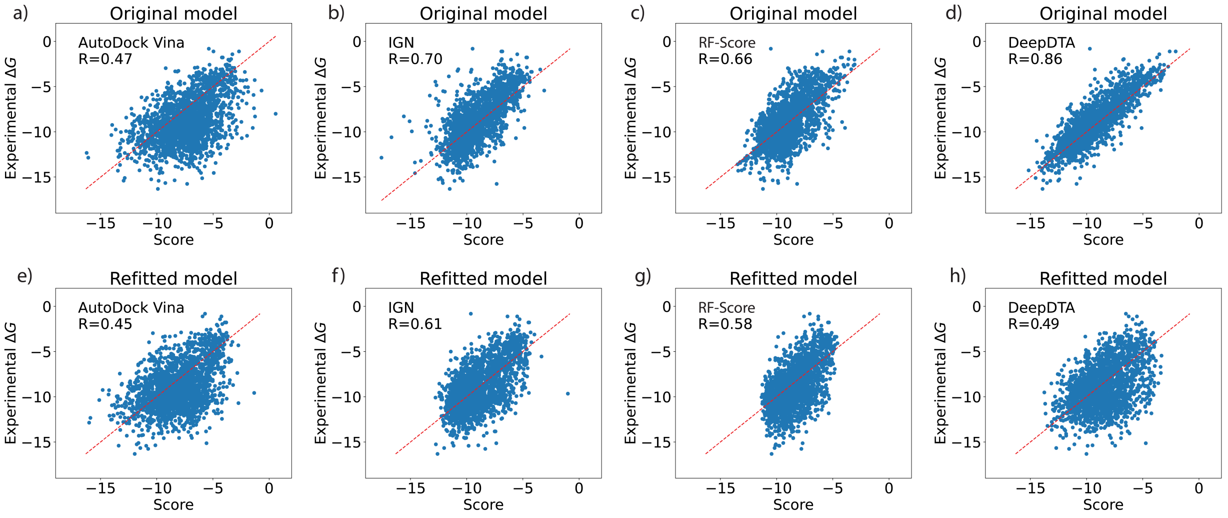

Figure 4: *Scatter plots for predicted scores and experimental binding affinities before and after model retraining for different models evaluated on UCBSplit test dataset.* Scatter plots for original models: (a) AutoDock vina, (b) IGN, (c) RF-Score, and (d) DeepDTA; Scatter plots for models retrained with UCBSplit: (e) AutoDock vina, (f) IGN, (g) RF-Score, and (h) DeepDTA.

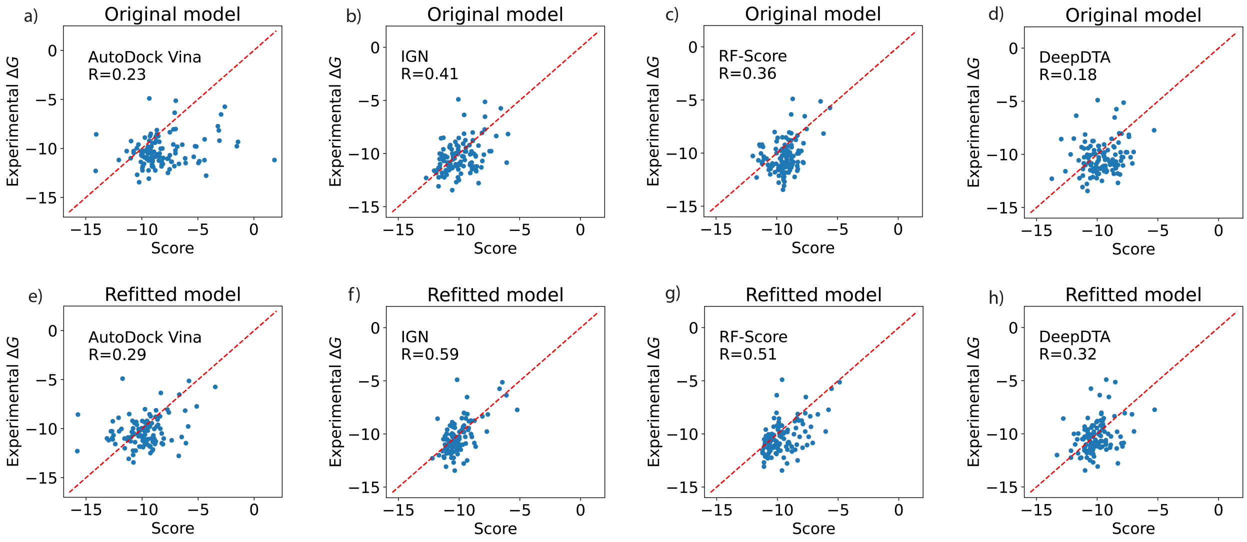

Figure 5: *Scatter plots for predicted scores and experimental binding affinities before and after model retraining for different models evaluated on BDB2020+ benchmark dataset.* Scatter plots for original models: AutoDock vina (a), IGN (b), RF-Score (c) and DeepDTA(d); Scatter plots for models retrained with UCBSplit: AutoDock vina (e), IGN (f), RF-Score (g) and DeepDTA (h)

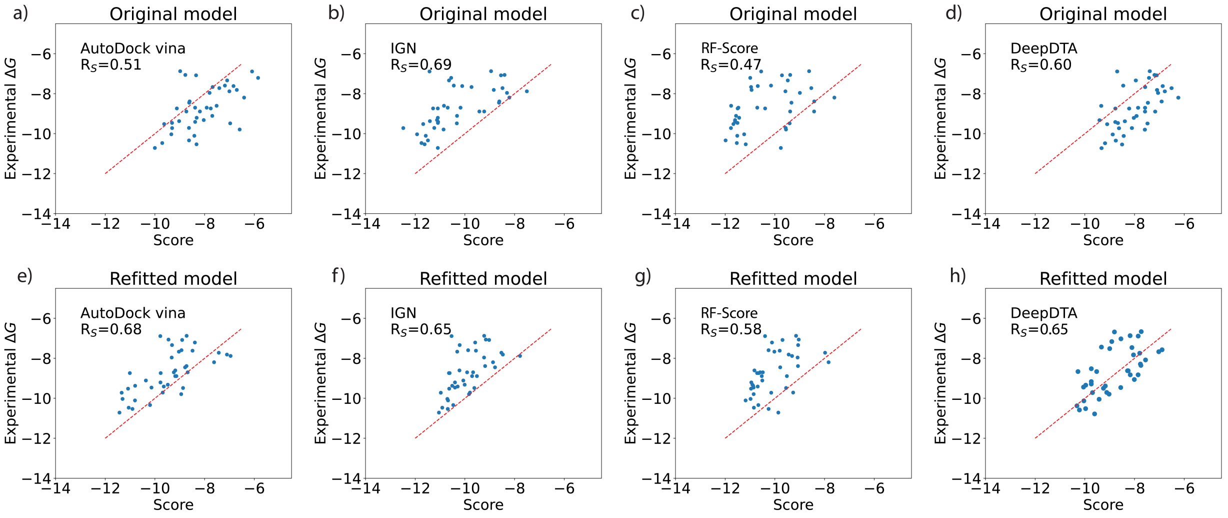

Figure 6: *Scatter plots for predicted scores and experimental binding affinities before and after model retraining for different models evaluated on SARS-CoV-2 main protease (Mpro) benchmark dataset.* Scatter plots for original models: AutoDock vina (a), IGN (b), RF-Score (c) and DeepDTA(d); Scatter plots for models retrained with UCBSplit: AutoDock vina (e), IGN (f), RF-Score (g) and DeepDTA (h)

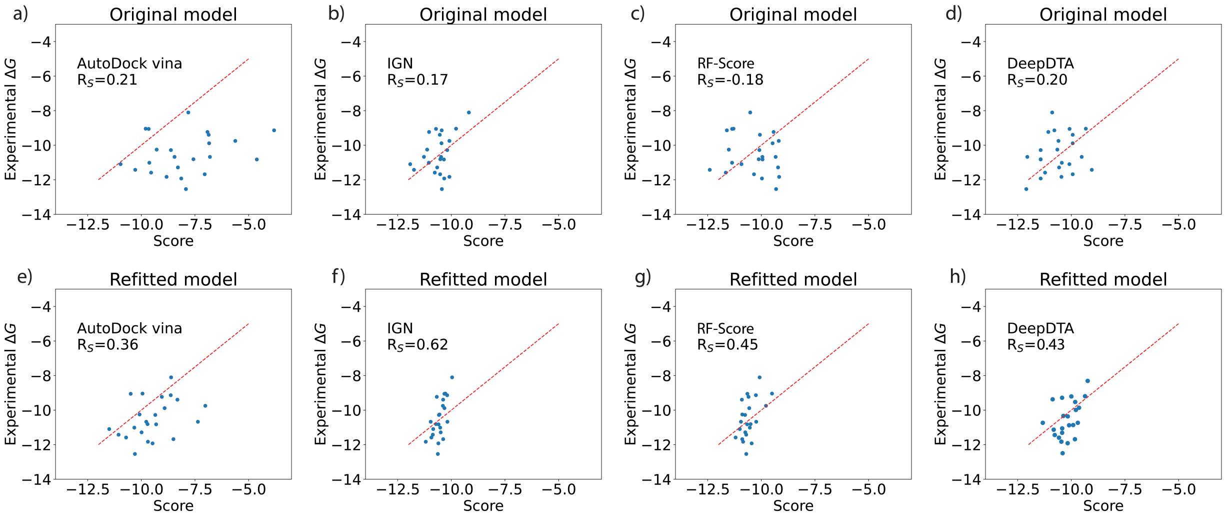

Figure 7: *Scatter plots for predicted scores and experimental binding affinities before and after model retraining for different models evaluated on epidermal growth factor receptor (EGFR) benchmark dataset.* Scatter plots for original models: AutoDock vina (a), IGN (b), RF-Score (c) and DeepDTA(d); Scatter plots for models retrained with UCBSplit: AutoDock vina (e), IGN (f), RF-Score (g) and DeepDTA (h)

### Covalent Binders

The identifiers of the 893 covalent binders in PDBBind v2020 (count: 893):

3HJ0, 2XLC, 6Q35, 4BCB, 5GWZ, 6P8X, 4DKT, 3B1T, 3BG8, 5E0G, 1EAS, 5WEJ, 1Z6F, 1BIO, 6B1H, 3BJM, 5I24, 3ZCZ, 4JJE, 6N4T, 4AXM, 2I72, 4QVM, 1KDW, 3HD3, 6HVS, 4R3B, 1P06, 5TG4, 5DGJ, 5DPA, 2OC1, 1P04, 5FAO, 4QWU, 5ZWH, 4WKS, 2O7V, 4GS6, 5C1Y, 1A3E, 4NO8, 1QX1, 2G5T, 1KE3, 3SN8, 4QWL, 3ZMH, 5T66, 3KJF, 5D9P, 5DP5, 3D4F, 1MXO, 1L6Y, 5WDL, 1LHE, 1PI4, 4BCC, 3LJ7, 1RHM, 5WAG, 6DA4, 2QL5, 6BL1, 5OM9, 6B1J, 5GWY, 1O45, 5OYD, 1QWU, 4OON, 1KDS, 6CHA, 6B1F, 4QWS, 6HHH, 6BKX, 5H6V, 3KW9, 1ONG, 4QVL, 5X02, 5J7S, 4AN1, 5FAT, 1P10, 1RHQ, 2H5D, 6RNI, 4DCD, 6DGE, 4WKV, 4AMZ, 5FA7, 6RNE, 4QZ2, 1QCP, 3P8E, 2AUX, 1I8J, 1K2I, 2FDA, 1O4K, 3NS7, 6B0Y, 1VSN, 5TYN, 1AU0, 1RWX, 1NC6, 5CYI, 4JG8, 4D8E, 3LLE, 1F1J, 3PA8, 6M9F, 3SNB, 4LEN, 4OOL, 4MNV, 1BGO, 1ZPC, 6B1Y, 3UR9, 6CN8, 4KQO, 1AHT, 2FS9, 6MKQ, 4GK7, 3NZI, 1QJ6, 4BS5, 4E3L, 4J70, 6HVU, 4PIQ, 1P01, 1F9E, 5INH, 6FV1, 3S1Y, 1RHJ, 4QW0, 5W14, 4MAO, 5DP6, 5HL9, 5ACB, 2MLM, 4O7D, 6ND3, 2WIJ, 4S2I, 6AFJ, 5KRE, 3TJM, 5TYL, 1GZG, 2G5P, 2WJ2, 2ZZ6, 4QVV, 3BM8, 2XNI, 3H0E, 2QNZ, 5SYS, 1Q6K, 4WX6, 5MJB, 4PKB, 6HVW, 1RTL, 3BH3, 4QW5, 5DP4, 1NY0, 3OYP, 1E34, 1U9W, 1YMX, 3SVV, 6SKD, 2WJ1, 4XBB, 5V2Q, 3W2P, 5VQY, 9LPR, 6DUD, 3LCE, 1GA9, 6HHI, 3RDH, 4Z16, 1YT7, 2XU5, 4ZRO, 5FOO, 1NMS, 2WOQ, 1FSW, 6HV7, 6NVG, 2NQG, 6UN3, 1QJ1, 5TTU, 3MXR, 6Q5B, 6CZU, 4YQV, 5JH6, 3SNA, 4WYY, 4IMZ, 5EEC, 6EYZ, 4F49, 5T6F, 2Y59, 4E3I, 3IKA, 6I0X, 1AYV, 4QZ3, 2XDM, 3KWB, 4LV3, 5L6O, 1RHR, 3ZMI, 6RN6, 8LPR, 4QWX, 4WKU, 1AU2, 1TMB, 3MBZ, 4PL5, 6FFS, 4XJR, 2LPR, 6FDU, 5TH7, 5HLB, 5MAJ, 2HWP, 1UOD, 1A46, 6NVI, 1GFW, 6QG7, 4WX4, 4J5P, 6QHR, 6FDQ, 3LPR, 5FAQ, 1MPL, 3VB5, 1FSY, 6MHB, 4X68, 3V4J, 6IUO, 6HVR, 4QPS, 6DB4, 4QBB, 1ORW, 2OZ2, 3EX6, 5WFJ, 6M8Y, 4X6J, 5VQV, 1NQC, 4EST, 6D3G, 6QHO, 1GMY, 6AFE, 2R4B, 2ALV, 5V2P, 4QW4, 4IMQ, 4XBD, 6HHG, 5J9Z, 2Y2N, 4YV8, 1INC, 5E7R, 5NE1, 3PR0, 6AFA, 3FMQ, 3O6T, 4OB2, 3FMR, 2WZX, 3W2Q, 6B95, 1O4I, 6RNU, 3QN7, 6S1S, 3KQA, 5VQX, 5U4G,

1MY8, 1ZPB, 4WKT, 3SZB, 6UN1, 6QW8, 4X69, 3ZEB, 4JG7, 6IB2, 3LOK, 6HUB, 7LPR, 6B1X, 1NYY, 5J87, 6IC6, 4WSK, 5SWH, 3V4X, 2FTD, 5YU9, 3VB7, 3O87, 4QVN, 4JK6, 5DG6, 6PNO, 6IYV, 5J8I, 3K84, 6NVJ, 5HG8, 2Y2I, 5VQE, 3ZVW, 2A4Q, 6HTD, 6BIC, 4WZ5, 1YLV, 3KJN, 5C91, 4JR0, 3UFA, 6S9W, 1IEM, 3A73, 1AD8, 6AFI, 5W13, 5J8X, 5WKL, 5NUD, 4E3N, 5X5G, 4YQU, 3BM6, 4QWI, 1H8Y, 2WGI, 5LCJ, 4WEF, 3QSD, 1TBZ, 2QLB, 1GGD, 2WIK, 4QVW, 1PAU, 6LPR, 4WM9, 4QVP, 6B41, 2XU1, 5TDI, 1ZOM, 3LOX, 2XDW, 6NVL, 2QQ7, 5DP8, 1YLY, 6M8W, 5XHR, 3SZ9, 4YAS, 1O4D, 3EWU, 2QKY, 5KYK, 6HVA, 1P02, 1ERO, 3GJS, 1W31, 2OP9, 3OPR, 1O43, 6HTR, 5GMP, 4VGC, 3OPP, 5WAD, 4GD6, 4NO1, 5LC0, 6O8I, 4WSJ, 4OLC, 1U9X, 6JPJ, 4QZ1, 3ZOT, 6K1S, 2XLN, 2XOW, 6G9F, 3VB6, 2EEP, 4QWR, 4MVN, 5LPR, 5TG2, 3FNM, 1MNS, 4MZO, 6DI1, 6MHM, 2AUZ, 1B0F, 2AJD, 3D62, 2Q9N, 1C3B, 5TYJ, 6RJP, 6PNN, 2Q3Z, 5J9Y, 4QXJ, 1P03, 6MZW, 5TTS, 3QKV, 3BLT, 5UG9, 3ZMJ, 2JAL, 3O1G, 4NNN, 1QHR, 4Q2K, 4PNC, 3KWZ, 4QQC, 6M9C, 3SV8, 1W12, 5V88, 6E5G, 2QLJ, 4JJ7, 2Y2K, 5ORL, 1A5G, 4I9O, 4NNW, 3ZVT, 2QLQ, 6M9D, 5TOZ, 5TYK, 6OVZ, 4QWJ, 2QLF, 3GPJ, 2Y4A, 2AJL, 3V6R, 4LV2, 2GVF, 1EXW, 4MNW, 4YHF, 6HHJ, 1BMQ, 4UUQ, 2GBG, 4WX7, 5ZWF, 4XUZ, 5HLD, 6AFL, 5D6F, 1IEW, 4TWY, 2O9A, 3C9E, 2HOB, 5YOF, 5L6P, 5TG6, 1VGC, 3O86, 1RHU, 4GHT, 4QZ4, 3G3M, 6CQZ, 1O4A, 5FQ9, 5EST, 5A3H, 1DOJ, 4E3J, 3K83, 5E0H, 4PIS, 5GTY, 3S22, 3S3Q, 5TEH, 1GBT, 1ERQ, 1NL6, 2XU4, 2H5I, 1A09, 4PL3, 2WIG, 1LHD, 4QW7, 7GCH, 2ZU4, 4QZX, 1W10, 4NO9, 5D11, 6F6R, 1LLB, 6DQB, 5TYP, 6E5B, 4E3K, 1LHG, 5LWN, 1LHF, 1RXP, 4MZS, 2ZU5, 4M1J, 6GOP, 6AFG, 1RWW, 4YRT, 4QWF, 1H1B, 5GNK, 4I7D, 3SND, 4PID, 5E0J, 1NJU, 1KE0, 5WAC, 2OC0, 1MS6, 2UZJ, 2AJB, 5JK3, 5TTV, 3HHA, 1NO9, 1NPZ, 5X79, 6PGP, 4INT, 2QL9, 5D6E, 2QCN, 6S9X, 3G3D, 6MHC, 3BLS, 1B5G, 4INH, 2OP3, 3MXS, 3VGC, 1RE1, 4XCU, 5UG8, 5VQZ, 2FM2, 6NVH, 2F9U, 3S3R, 1L6S, 2GBF, 4HCV, 4PL4, 3W2T, 6AFC, 4I7C, 6HUQ, 1G37, 6AFH, 2YJB, 5DP7, 5U4F, 4M8T, 5DP9, 4QVY, 3KRD, 4JMX, 2XU3, 4WBG, 4NO6, 2F9V, 2BDL, 6RN9, 2Y2J, 2YJC, 4QZ0, 4CCD, 1W14, 6N9P, 4QWG, 4PJI, 5GSO, 4HRD, 6MNY, 6G8N, 2V6N, 4MNX, 3BLU, 6HUC, 6G7F, 2XK1, 6OIM, 5WKM,

5FAS, 4E3M, 4D8I, 5TG1, 6HTC, 5V4Q, 4WMC, 6ALZ, 3HWN, 4MBF, 2HWO, 4QZ5, 1P05, 3MKF, 5C1X, 6CQT, 6B1O, 4YEC, 6QFT, 1EAT, 4HRC, 5WAE, 1IAU, 2ZU3, 6IYW, 3RJM, 1U9V, 1AUJ, 2QL7, 4HNP, 2Y2P, 6IC5, 3E90, 1YM1, 6FV2, 5W12, 1TYN, 6BL2, 4QKX, 3ZIM, 1QTN, 4AMY, 2NQI, 3OF8, 3EX3, 2H65, 5NE3, 1LHC, 1EKB, 1SNK, 5AHJ, 1MEM, 4KIO, 6ARY, 3OVX, 4INR, 5ZWE, 1NLJ, 1RHK, 4HCU, 2FXR, 4AMX, 6BIB, 4U0X, 6QW9, 1YK7, 2I03, 3LXS, 3SNC, 1ONH, 4QW1, 1TU6, 3B1U, 6B1W, 6HGY, 5UGC, 2Z3Z, 6MHD, 4MZ4, 4MNY, 5VND, 4JK5, 2QAF, 3OJ8, 6GCH, 1MWT, 6P8Y, 3KJQ, 4YRS, 6HUV, 2RCX, 2H5J, 5LVX, 4QWK, 5F02, 6D8E, 5WAF, 5EE8, 4QW3, 4NK3, 4JG6, 5G0Q, 2G63, 2Y55, 6B0V, 5MAE, 4R02, 5T6G, 5LCK, 6HUU, 5HG9, 6AFD, 6ERT, 4R6V, 4INU, 1A61, 6QG4, 5NGF, 4FZG, 5FAP, 4QQ5, 2WAP, 6GCR, 6BID, 5TYO, 4HBP, 2OBO, 6RTN, 5TG5, 4QZ7, 3EYD, 1PI5, 5MQY, 3SV7, 6HVT, 6GXY, 2FJ0, 4JJ8, 6RN7, 2XE4, 6QWB, 3VB4, 2A4G, 2Q9M, 4BSQ, 4AMW, 5ZA2, 3U1I, 3FKV, 6H0U, 4QW6, 3T9T, 1O41, 2FS8, 1E37, 3I4A, 6HV5, 4YQM, 4LV1, 6B1E, 2Y2H, 3BWK, 2YJ9, 1O4E, 5I23, 2OC7, 1AYU, 6FFN, 1QJ7, 5XYZ, 6AFF, 5TG7, 3BAR, 3I06, 5GWA, 5J5D, 4E3O, 6HVV, 4QZ6, 6PGO, 4WZ4, 2XZC, 1HBJ, 5HG7, 6G9S, 6AX1, 2LP8, 6GZY, 2YJ8, 4Q1S, 6AF9, 6QW7, 4L0L, 3SJO, 1AWF, 1F7B, 1TLO, 1QFS, 6IB0, 4FZC, 5NPB, 5NWZ, 6QWA, 1YMS, 4QZW, 2WZZ, 3IUT, 6SKB, 6J6M, 5C20, 1AWH, 2VGC, 1NKM, 6HV4, 6G8M, 6BQ0, 2R6N, 5HG5, 3N4C, 3K7F, 6PNM, 3SJI, 4IVK, 4QVQ, 4DMY, 6P8Z, 4RSP, 5CLS, 3O88, 2YJ2, 3V6S, 4X0U, 4AN0, 6HTP, 2XCN, 3SV6

## Structures Containing Steric Clashes in PDBBind

Here are the PDBIDs for structures in PDBBind v2020 with minimum distance between heavy atoms in the ligand and the protein less than 1.75Å (count: 1303): 3N9S, 4U1B, 3ZHX, 1EW8, 2FZK, 2Q8Z, 5GOF, 1EX8, 1OLX, 3A6T, 1KM3, 4K55, 5EFA, 3K1J, 4L51, 3NXQ, 6D1H, 1FZQ, 1JLR, 6O5T, 1I9N, 4U6C, 2ZZ2, 1X8R, 4U71, 2RK8, 2V8W, 6HH3, 4FL2, 1RNM, 3ISS, 3DJV, 1PVN, 4U6W, 1B38, 1MAI, 1V48, 2C94, 1LCP, 3B7I, 1V11, 4EU0,

4R3W, 6CZB, 4NXV, 6CZC, 6D1G, 1NKI, 3B7R, 4K5P, 2V2H, 1W4Q, 1MRS, 1AFL, 2ISW, 4AXD, 3HU3, 3EVD, 1M83, 4DEL, 3RV4, 3IAE, 6D1B, 4UOH, 3B3C, 1WUQ, 3WGG, 4U0W, 3RBU, 3AHO, 6JDI, 1G98, 3A1E, 4K3N, 4QJX, 2OI2, 1JVU, 1B55, 2W8W, 6IHT, 2RCN, 2R75, 3D2E, 3PCG, 2GSU, 4NCN, 4KAX, 2C92, 1TLP, 4QRH, 2YAY, 1TKB, 3S8L, 1YQ7, 1G53, 1KSN, 4DCS, 1JYQ, 1QK4, 4P5D, 1FT7, 6D9S, 4PVY, 6OF5, 2QTN, 5D6J, 2AMT, 4L50, 4IDN, 6D1J, 1WUR, 1RNT, 1SLN, 1BXR, 3G1V, 1PKX, 4ZCS, 5CBM, 1LVU, 6JON, 3I73, 3EBI, 3EXH, 2TMN, 5HVA, 4U69, 3OVE, 2WF5, 4JNE, 4FL1, 2E91, 1M5W, 5FSX, 2XBP, 4U73, 5HWU, 2WE3, 6FAA, 6D15, 1RP7, 6D3Q, 5NW7, 1M7Y, 1YFZ, 1U0G, 2PY4, 4LPS, 1YDD, 5LTN, 3DJO, 3K5X, 2QTA, 3BXF, 5TMP, 2RIO, 1PFU, 2PU1, 1B57, 3A1C, 3D7K, 1S5Z, 1M0Q, 1A4R, 6J72, 3UPK, 4U70, 1V16, 6MNV, 3JUK, 4U6Z, 1ATR, 6FHK, 3OZG, 1OLS, 3EGT, 3AHN, 3OV1, 3AAS, 6DD0, 3Q71, 3EXE, 3FZN, 6D1A, 1D2E, 2E92, 3N1C, 1MUE, 5EVZ, 4ETZ, 4JYC, 6D18, 2PQC, 3C56, 6HH5, 5C2O, 3FZY, 1HYO, 5BV3, 2NSL, 6H77, 3I9G, 5UV2, 1HI4, 3DJP, 2R59, 4DY6, 1EW9, 2VT3, 2FXV, 5YHE, 5LDP, 1Q54, 2WZF, 3FUC, 2W5G, 2PTZ, 5F2R, 5HJQ, 1X8T, 2X97, 4OR6, 1VSO, 3MKE, 3HJ0, 2XLC, 6Q35, 4BCB, 5KHG, 3LOO, 3L0K, 5TIG, 6JN4, 2Y4M, 6EJI, 2PJ4, 4QIR, 3BG8, 1QF5, 6G15, 1EAS, 1Z6F, 1BIO, 6B1H, 3BJM, 5I24, 3ZCZ, 5YBI, 3HU2, 6N4T, 2I72, 4QVM, 1KDW, 3POA, 1TMM, 4EFG, 6HVS, 3GGC, 2VF6, 4R3B, 1SRE, 1P06, 5O3R, 5TG4, 5JNL, 1NU8, 2OC1, 3ATV, 1P04, 5FAO, 5IZM, 4QWU, 2CFG, 6N97, 5EOU, 4WKS, 4OCP, 5C1Y, 3UIG, 2Y1G, 4NO8, 1ONP, 2G5T, 1KE3, 4QWL, 3ZMH, 5T66, 3KR5, 5D9P, 1DB4, 1WC6, 3D4F, 4F3H, 1MXO, 1L6Y, 1Y8P, 1LHE, 2PJA, 1PI4, 4ZQT, 4BCC, 3LJ7, 6CZD, 5JEK, 5WAG, 3TDH, 6DA4, 3UED, 3WNS, 5OM9, 6B1J, 3EWZ, 1O45, 3D9P, 5OYD, 1QWU, 1H79, 4DXJ, 4OON, 1KDS, 6B1F, 4UMJ, 4QWS, 1TKC, 6BKX, 5H6V, 1ONG, 5ZDG, 4KXM, 6I5J, 4P4F, 4QVL, 5X02, 5J7S, 5J7J, 4AN1, 4DN0, 4P4S, 5FAT, 1T29, 1P10, 1R0X, 2H5D, 5B5P, 1QJB, 4WKV, 4AMZ, 5FA7, 6FEL, 6RNE, 1Y8O, 4QZ2, 1QCP, 3GQO, 2MC1, 3ZP9, 2AUX, 2PJC, 1I8J, 3E0P, 1K2I, 6G01, 2FDA, 1O4K, 6AEC, 5W38, 3QXC, 6B22, 6B0Y, 1VSN, 6FA5, 4YX9, 5TYN, 4KP4, 6NNG, 5CYI, 4BXN, 4G8L, 3PA8, 6FAC, 6IPL, 6M9F, 4K5N, 4LEN, 1AO0, 4OOL, 4MNV, 4E1E, 1ZPC, 1KWR,

6B1Y, 3FYZ, 5L44, 4YB7, 6R4V, 1CSI, 1H7A, 2Z7H, 1OTH, 4KQO, 5F1C, 4JJF, 4EEJ, 1AHT, 2FS9, 6MKQ, 4GK7, 3NZI, 1QJ6, 4E3L, 2J4K, 4J70, 6HVU, 1P01, 5INH, 1H07, 3S1Y, 4QW0, 5W14, 5DP6, 5HL9, 4WYZ, 5ACB, 1TPZ, 4O7D, 3X1K, 2WIJ, 4S2I, 5ICV, 5KRE, 4R17, 3TJM, 2JBV, 5TYL, 4QLU, 2QPJ, 3TL0, 2G5P, 2WJ2, 2ZZ6, 4QVV, 2XNI, 2QNZ, 6AGP, 4PKB, 6HVW, 1LF8, 1RTL, 3BH3, 4QW5, 1EBG, 1NY0, 1E34, 1U9W, 2IOA, 1YMX, 4K8O, 6SKD, 2Z7I, 2WJ1, 1TL7, 1X07, 2Y1D, 9LPR, 1GA9, 1MN9, 3WDC, 6AK6, 4QLV, 6R4S, 6FMP, 3RDH, 3DPC, 5FOO, 2WOQ, 1FSW, 2PJ5, 6HV7, 5AB0, 3RYW, 6UN3, 1QJ1, 5TTU, 3L0N, 6AEH, 3MXR, 6Q5B, 1CSS, 6CZU, 4R18, 1G05, 5JH6, 6OM4, 4JG0, 4WYY, 4IMZ, 5EEC, 6EYZ, 3HU1, 2VHQ, 5NPF, 2Y59, 4E3I, 6PK7, 5UV1, 5Z68, 4K5M, 6IPM, 4QZ3, 2XDM, 4LV3, 2C1N, 3AAV, 4FUT, 4FR3, 3ZMI, 1PPW, 8LPR, 5BRN, 1NZV, 4QWX, 5F29, 4WKU, 3EXF, 6N54, 4GKC, 4RAB, 3MBZ, 4PL5, 4JJG, 2XAQ, 2P0X, 4XJR, 6IPH, 2LPR, 6FDU, 4U6E, 5HLB, 3HWX, 1UOD, 4DEM, 1P6D, 1AKQ, 1LV8, 1AMN, 6QG7, 4J5P, 6FDQ, 5NPR, 3FHE, 3LPR, 5FAQ, 1MPL, 5L6H, 3VB5, 1FSY, 4X68, 3V4J, 4QLQ, 6HVR, 1O8B, 6NZG, 4HWT, 6DB4, 1ML1, 1AKV, 5JAZ, 1ORW, 2OZ2, 3EX6, 5WFJ, 6M8Y, 4X6J, 1B39, 1NQC, 5UQ9, 4EST, 5W10, 4JN4, 6D3G, 1EIX, 1RYH, 6N94, 4QW4, 2A4R, 5YR5, 1KF0, 1D7X, 2QTR, 5EY8, 5JMP, 5J9Z, 2Y2N, 4YV8, 1INC, 3T2C, 3LXO, 6MLF, 1E8H, 5NE1, 3PR0, 1JBD, 3FMQ, 4OB2, 3FMR, 2WZX, 1O4I, 6RNU, 6S1S, 5U4G, 1MY8, 1YHM, 2C9D, 3D67, 5YR6, 1ZPB, 4WKT, 6UN1, 6QW8, 4X69, 3ZEB, 6IB2, 6HUB, 7LPR, 3ZKF, 4UV9, 5K6S, 2Z4W, 6B1X, 1NYY, 5IZL, 4WSK, 3VB7, 3K41, 3O87, 5BSK, 4QVN, 3L6F, 3CST, 5OD5, 3K84, 1VJC, 2Y2I, 5VQE, 3ZVW, 2A4Q, 4WUP, 6HTD, 5F5B, 1C7F, 4LOJ, 4WZ5, 3SHV, 1YLV, 2QM7, 1YON, 1VJD, 1U2R, 3ZY2, 3E16, 6Q30, 2Y4L, 1IEM, 1AD8, 6EUV, 5W13, 2QCD, 4I9S, 4GA3, 5AGJ, 5J8X, 4L2X, 4BG6, 5NUD, 3TDZ, 5KHD, 3AU6, 1GGN, 4E3N, 5X5G, 4EHM, 3GZN, 5NHZ, 3BM6, 4QWI, 1H8Y, 2WQP, 2WGI, 6J7L, 1RU2, 4NZM, 1TBZ, 3AXK, 5YKP, 4LIL, 4HE9, 1RGK, 1GGD, 5NZ2, 2WIK, 6RMM, 4QVW, 6LPR, 4WM9, 4QVP, 5GSW, 2PU0, 1ZOM, 6PXC, 5FB0, 1Y19, 3LOX, 1T3T, 4CLP, 2XDW, 1YLY, 6M8W, 5XHR, 4YAS, 1O4D, 1I9L, 3EWU, 2QKY, 2ORK, 5KYK, 6ISD, 6HVA, 6DD1, 1P02, 1ERO, 6GFM, 1AKU, 1W31, 3OPR,

1O43, 4RHU, 2PJ0, 6HTR, 6AR2, 3MLE, 4VGC, 6HYS, 3OPP, 3LLM, 4U0G, 5WAD, 1I9Q, 4EXZ, 4Y67, 4GD6, 4H38, 4NO1, 5LC0, 6E5S, 4FL3, 6AK5, 4WSJ, 1U9X, 4QZ1, 5NYZ, 3WDZ, 3ZOT, 3ZMQ, 6K1S, 2XLN, 2XOW, 3WNR, 6G9F, 3V7D, 3KC0, 3VB6, 4QWR, 3NCQ, 4MVN, 5LPR, 4A0J, 3RG2, 3Q7P, 1QJA, 3FNM, 3IQV, 3IUC, 1MNS, 3RBM, 4OAZ, 2AUZ, 6IA7, 4G5Y, 6G2N, 1B0F, 2AJD, 3D62, 2Q9N, 1C3B, 6GIU, 5TYJ, 6RJP, 1BWN, 2Q3Z, 1I9M, 1NJT, 5J9Y, 4U0U, 2L0I, 4KW6, 4QXJ, 1NZL, 2Y8L, 1P03, 4ZCW, 3EX2, 3QKV, 3BLT, 4UVA, 3ZMJ, 3NBA, 2JAL, 4NNN, 5NPS, 1QHR, 6C5J, 4Q2K, 4W5J, 4EDE, 4LQY, 4QQC, 2E95, 4GS9, 4I9R, 6M9C, 4P5E, 3SV8, 1W12, 2V0C, 2HQU, 4OEL, 6AK4, 2E98, 1O4F, 5J7P, 2Y2K, 3QT7, 2O1V, 4NNW, 3ZS1, 3ATU, 3ZVT, 2QLQ, 1RU1, 6M9D, 5ZDC, 5TYK, 6B4H, 6OVZ, 2HA0, 4QWJ, 3GPJ, 2Y4A, 3OB2, 2H5A, 2AJL, 1BJR, 3Q72, 4LV2, 2CMC, 2GVF, 5HH4, 4UUQ, 2GBG, 5W8V, 4WX7, 2ZA3, 5ZWF, 4XUZ, 1AKR, 5HLD, 3CF1, 1MF4, 5D6F, 4WN0, 2PJ6, 1IEW, 6BS5, 2HOB, 4DWG, 5YOF, 5TG6, 1VGC, 5DOH, 3O86, 1X6U, 4QZ4, 3G3M, 6CQZ, 1O4A, 5FQ9, 5EST, 4PRY, 5A3H, 1DOJ, 4E3J, 3K83, 4AY6, 1GVK, 6IPJ, 3S22, 5TEH, 3WYJ, 1GBT, 6PGX, 4W4S, 1ERQ, 2E93, 3G4F, 4N1Z, 1A09, 4PL3, 2WIG, 3EHW, 1LHD, 1AZL, 4QW7, 7GCH, 5MXQ, 2Z52, 2O4H, 3BH8, 4QZX, 1W10, 4NO9, 5D11, 1LLB, 5TYP, 5IM3, 1CSH, 6E5B, 4E3K, 1LHG, 2A29, 1NFS, 1Y98, 3U8D, 1LHF, 1RXP, 4M1J, 6GOP, 5HYX, 4QWF, 5YR4, 1H1B, 6QXS, 3A1S, 4PID, 4GV8, 1NJU, 2QCG, 1KE0, 5WAC, 2OC0, 6IPI, 6EGS, 3Q2G, 2AJB, 5W19, 5JK3, 1NO9, 5MOC, 6E7M, 5MI3, 5SVK, 5X79, 4INT, 4RXC, 3FCK, 1LRT, 5H63, 5D6E, 2QCN, 3G3D, 6AI9, 3V0P, 3BLS, 1H9L, 2OP3, 3MXS, 3VGC, 6NPF, 5L6J, 2FM2, 2F9U, 1VKJ, 2Q80, 4TMF, 1L6S, 5EWZ, 2GBF, 4PL4, 1RYF, 3W2T, 1XFV, 6HUQ, 5WRS, 1G37, 1CSR, 4JBS, 1MAU, 3QX8, 5DP7, 4M8T, 4QVY, 4K5O, 3KRD, 4JMX, 5AEL, 4RAQ, 4WBG, 6N9T, 5OLK, 2PJ2, 2K0X, 4NO6, 1FPY, 2F9V, 2BDL, 6RN9, 4K19, 2Y2J, 4QZ0, 3E4A, 4CCD, 1W14, 6N9P, 6RML, 4U82, 4QWG, 4PJI, 5GSO, 1GX4, 5M9D, 4HRD, 6G8N, 3BB1, 4QLS, 2E99, 3BLU, 6HUC, 6G7F, 3IOI, 2XK1, 5EY9, 4GZ3, 3UX0, 3UO9, 5FAS, 4E3M, 6HTC, 5V4Q, 4WMC, 3PJT, 4MBF, 4QZ5, 1P05, 3MKF, 5C1X, 6CQT, 1NYM, 6B1O, 6QFT, 1GX0, 3CPH, 5HH6, 1EAT, 1U0H, 4HRC, 4EUV, 5WAE, 1IAU, 2H1H, 1OXG, 3RJM,

1AUJ, 5EXX, 4HNP, 2Y2P, 6IC5, 3E90, 6H78, 3D9L, 1YM1, 5G1P, 4RAC, 5W12, 1TYN, 6BL2, 2ITK, 2G83, 3FV7, 3ZIM, 1QTN, 4AMY, 5HH5, 3EX3, 5NE3, 1LHC, 5A3R, 1EKB, 5AHJ, 5E2V, 3BUO, 6ARY, 6MMC, 4INR, 2FXR, 4AMX, 4U0X, 6QW9, 2P59, 2I03, 1BSK, 3D9M, 1ONH, 4QW1, 4K5L, 1I3Z, 6B1W, 2QCF, 3BU6, 2P1C, 1FT4, 5VND, 4YM4, 2QAF, 4DWB, 2PLL, 3RSB, 3LDW, 16PK, 3OJ8, 6GCH, 1MWT, 2QCM, 4V11, 6HUV, 2RCX, 5LVX, 4QWK, 2JG8, 5WAF, 5EE8, 4QW3, 4NK3, 1ELS, 2IT4, 5G0Q, 2WVA, 3B9S, 2G63, 2Y55, 6B0V, 5MAE, 4R02, 6IPK, 6HUU, 1YYY, 6ERT, 4R6V, 4INU, 1G9R, 5OSY, 4FZG, 2JBJ, 5FAP, 4QQ5, 2WAP, 5TYO, 5X9H, 4HBP, 2OBO, 6RTN, 5TG5, 1I9O, 4QZ7, 3EYD, 1PI5, 3SV7, 6HVT, 2FJ0, 5E2N, 2XE4, 6QWB, 4KXL, 3VB4, 2A4G, 2Q9M, 4BSQ, 5ZA2, 2WIC, 6G6X, 3U1I, 4JFX, 5KNU, 3FKV, 5JBI, 4QW6, 1O41, 2FS8, 1E37, 6CSE, 5LVF, 4OSF, 6HV5, 4LV1, 6B1E, 2Y2H, 3KSL, 1O4E, 5I23, 2OC7, 4RUU, 1QJ7, 5XYZ, 5KNR, 5K7H, 1SZ0, 6BUU, 5TG7, 2AD5, 3BAR, 4AY5, 5GWA, 4E3O, 6NNR, 6HVV, 3FZC, 4QZ6, 1M9N, 4WZ4, 2XZC, 1HBJ, 6G9S, 6AX1, 5EJE, 1TKA, 6GZY, 2L75, 3FSJ, 4OEM, 4Q1S, 3NCR, 5L6I, 6MU1, 6QW7, 4L0L, 5NI0, 4QLT, 1AWF, 1F7B, 6AEJ, 1QFS, 6IB0, 4FZC, 6OTT, 5NPB, 6QWA, 2F89, 1BA8, 2PSX, 4RAO, 3ZJC, 1YMS, 4QZW, 2WZZ, 2Y8Q, 6CJJ, 4GE5, 6SKB, 1AWH, 3HXE, 5JC1, 2VGC, 1SYO, 1NKM, 6HV4, 6G8M, 6BQ0, 3UEC, 5ZDE, 1JRS, 3K7F, 4IVK, 3WNT, 3BGM, 4QVQ, 2HR6, 5CLS, 3O88, 4P4T, 4X0U, 6E3P, 2ONB, 4AN0, 6HTP, 2XCN, 3SV6
