# Supplementary material for: Leak Proof PDBBind: A Reorganized Dataset of Protein-Ligand Complexes for More Generalizable Binding Affinity Prediction
Source: ArXiv. 2023 Aug 18:arXiv:2308.09639v1. Preprint. [Version 1] (PMC10462179)
Supplement: Supplementary file 1 [file NIHPP2308.09639V1-supplement-1.pdf]

arXiv:2308.09639v1 [physics.bio-ph] 18 Aug 2023

Supporting Information
Leak Proof PDBBind: A Reorganized Dataset of Protein-Ligand Complexes for More Generalizable Binding Affinity Prediction

Jie Li,[†] Xingyi Guan,[†] Oufan Zhang,[†] Kunyang Sun,[†] Yingze Wang,[†] Dorian Bagni,[†] and Teresa Head-Gordon[*,†]

† *Pitzer Center for Theoretical Chemistry, Department of Chemistry, University of California, Berkeley CA 94720, USA*

‡*Departments of Bioengineering and Chemical and Biomolecular Engineering, University of California, Berkeley, CA, USA*

E-mail: thg@berkeley.edu

Covalent Binders in PDBBind v2020

These are the PDBIDs for covalent binders in PDBBind v2020 (count: 893):

3HJ0, 2XLC, 6Q35, 4BCB, 5GWZ, 6P8X, 4DKT, 3B1T, 3BG8, 5E0G, 1EAS, 5WEJ, 1Z6F, 1BIO, 6B1H, 3BJM, 5I24, 3ZCZ, 4JJE, 6N4T, 4AXM, 2I72, 4QVM, 1KDW, 3HD3, 6HVS, 4R3B, 1P06, 5TG4, 5DGJ, 5DPA, 2OC1, 1P04, 5FAO, 4QWU, 5ZWH, 4WKS, 2O7V, 4GS6, 5C1Y, 1A3E, 4NO8, 1QX1, 2G5T, 1KE3, 3SN8, 4QWL, 3ZMH, 5T66, 3KJF, 5D9P, 5DP5, 3D4F, 1MXO, 1L6Y, 5WDL, 1LHE, 1PI4, 4BCC, 3LJ7, 1RHM, 5WAG, 6DA4, 2QL5, 6BL1, 5OM9, 6B1J, 5GWY, 1O45, 5OYD, 1QWU, 4OON, 1KDS, 6CHA, 6B1F, 4QWS, 6HHH,

6BKX, 5H6V, 3KW9, 1ONG, 4QVL, 5X02, 5J7S, 4AN1, 5FAT, 1P10, 1RHQ, 2H5D, 6RNI, 4DCD, 6DGE, 4WKV, 4AMZ, 5FA7, 6RNE, 4QZ2, 1QCP, 3P8E, 2AUX, 1I8J, 1K2I, 2FDA, 1O4K, 3NS7, 6B0Y, 1VSN, 5TYN, 1AU0, 1RWX, 1NC6, 5CYI, 4JG8, 4D8E, 3LLE, 1F1J, 3PA8, 6M9F, 3SNB, 4LEN, 4OOL, 4MNV, 1BGO, 1ZPC, 6B1Y, 3UR9, 6CN8, 4KQO, 1AHT, 2FS9, 6MKQ, 4GK7, 3NZI, 1QJ6, 4BS5, 4E3L, 4J70, 6HVU, 4PIQ, 1P01, 1F9E, 5INH, 6FV1, 3S1Y, 1RHJ, 4QW0, 5W14, 4MAO, 5DP6, 5HL9, 5ACB, 2MLM, 4O7D, 6ND3, 2WIJ, 4S2I, 6AFJ, 5KRE, 3TJM, 5TYL, 1GZG, 2G5P, 2WJ2, 2ZZ6, 4QVV, 3BM8, 2XNI, 3H0E, 2QNZ, 5SYS, 1Q6K, 4WX6, 5MJB, 4PKB, 6HVW, 1RTL, 3BH3, 4QW5, 5DP4, 1NY0, 3OYP, 1E34, 1U9W, 1YMX, 3SVV, 6SKD, 2WJ1, 4XBB, 5V2Q, 3W2P, 5VQY, 9LPR, 6DUD, 3LCE, 1GA9, 6HHI, 3RDH, 4Z16, 1YT7, 2XU5, 4ZRO, 5FOO, 1NMS, 2WOQ, 1FSW, 6HV7, 6NVG, 2NQG, 6UN3, 1QJ1, 5TTU, 3MXR, 6Q5B, 6CZU, 4YQV, 5JH6, 3SNA, 4WYY, 4IMZ, 5EEC, 6EYZ, 4F49, 5T6F, 2Y59, 4E3I, 3IKA, 6I0X, 1AYV, 4QZ3, 2XDM, 3KWB, 4LV3, 5L6O, 1RHR, 3ZMI, 6RN6, 8LPR, 4QWX, 4WKU, 1AU2, 1TMB, 3MBZ, 4PL5, 6FFS, 4XJR, 2LPR, 6FDU, 5TH7, 5HLB, 5MAJ, 2HWP, 1UOD, 1A46, 6NVI, 1GFW, 6QG7, 4WX4, 4J5P, 6QHR, 6FDQ, 3LPR, 5FAQ, 1MPL, 3VB5, 1FSY, 6MHB, 4X68, 3V4J, 6IUO, 6HVR, 4QPS, 6DB4, 4QBB, 1ORW, 2OZ2, 3EX6, 5WFJ, 6M8Y, 4X6J, 5VQV, 1NQC, 4EST, 6D3G, 6QHO, 1GMY, 6AFE, 2R4B, 2ALV, 5V2P, 4QW4, 4IMQ, 4XBD, 6HHG, 5J9Z, 2Y2N, 4YV8, 1INC, 5E7R, 5NE1, 3PR0, 6AFA, 3FMQ, 3O6T, 4OB2, 3FMR, 2WZX, 3W2Q, 6B95, 1O4I, 6RNU, 3QN7, 6S1S, 3KQA, 5VQX, 5U4G, 1MY8, 1ZPB, 4WKT, 3SZB, 6UN1, 6QW8, 4X69, 3ZEB, 4JG7, 6IB2, 3LOK, 6HUB, 7LPR, 6B1X, 1NYY, 5J87, 6IC6, 4WSK, 5SWH, 3V4X, 2FTD, 5YU9, 3VB7, 3O87, 4QVN, 4JK6, 5DG6, 6PNO, 6IYV, 5J8I, 3K84, 6NVJ, 5HG8, 2Y2I, 5VQE, 3ZVW, 2A4Q, 6HTD, 6BIC, 4WZ5, 1YLV, 3KJN, 5C91, 4JR0, 3UFA, 6S9W, 1IEM, 3A73, 1AD8, 6AFI, 5W13, 5J8X, 5WKL, 5NUD, 4E3N, 5X5G, 4YQU, 3BM6, 4QWI, 1H8Y, 2WGI, 5LCJ, 4WEF, 3QSD, 1TBZ, 2QLB, 1GGD, 2WIK, 4QVW, 1PAU, 6LPR, 4WM9, 4QVP, 6B41, 2XU1, 5TDI, 1ZOM, 3LOX, 2XDW, 6NVL, 2QQ7, 5DP8, 1YLY, 6M8W, 5XHR, 3SZ9, 4YAS, 1O4D, 3EWU, 2QKY, 5KYK, 6HVA, 1P02, 1ERO, 3GJS, 1W31, 2OP9, 3OPR, 1O43, 6HTR,

5GMP, 4VGC, 3OPP, 5WAD, 4GD6, 4NO1, 5LC0, 6O8I, 4WSJ, 4OLC, 1U9X, 6JPJ, 4QZ1, 3ZOT, 6K1S, 2XLN, 2XOW, 6G9F, 3VB6, 2EEP, 4QWR, 4MVN, 5LPR, 5TG2, 3FNM, 1MNS, 4MZO, 6DI1, 6MHM, 2AUZ, 1B0F, 2AJD, 3D62, 2Q9N, 1C3B, 5TYJ, 6RJP, 6PNN, 2Q3Z, 5J9Y, 4QXJ, 1P03, 6MZW, 5TTS, 3QKV, 3BLT, 5UG9, 3ZMJ, 2JAL, 3O1G, 4NNN, 1QHR, 4Q2K, 4PNC, 3KWZ, 4QQC, 6M9C, 3SV8, 1W12, 5V88, 6E5G, 2QLJ, 4JJ7, 2Y2K, 5ORL, 1A5G, 4I9O, 4NNW, 3ZVT, 2QLQ, 6M9D, 5TOZ, 5TYK, 6OVZ, 4QWJ, 2QLF, 3GPJ, 2Y4A, 2AJL, 3V6R, 4LV2, 2GVF, 1EXW, 4MNW, 4YHF, 6HHJ, 1BMQ, 4UUQ, 2GBG, 4WX7, 5ZWF, 4XUZ, 5HLD, 6AFL, 5D6F, 1IEW, 4TWY, 2O9A, 3C9E, 2HOB, 5YOF, 5L6P, 5TG6, 1VGC, 3O86, 1RHU, 4GHT, 4QZ4, 3G3M, 6CQZ, 1O4A, 5FQ9, 5EST, 5A3H, 1DOJ, 4E3J, 3K83, 5E0H, 4PIS, 5GTY, 3S22, 3S3Q, 5TEH, 1GBT, 1ERQ, 1NL6, 2XU4, 2H5I, 1A09, 4PL3, 2WIG, 1LHD, 4QW7, 7GCH, 2ZU4, 4QZX, 1W10, 4NO9, 5D11, 6F6R, 1LLB, 6DQB, 5TYP, 6E5B, 4E3K, 1LHG, 5LWN, 1LHF, 1RXP, 4MZS, 2ZU5, 4M1J, 6GOP, 6AFG, 1RWW, 4YRT, 4QWF, 1H1B, 5GNK, 4I7D, 3SND, 4PID, 5E0J, 1NJU, 1KE0, 5WAC, 2OC0, 1MS6, 2UZJ, 2AJB, 5JK3, 5TTV, 3HHA, 1NO9, 1NPZ, 5X79, 6PGP, 4INT, 2QL9, 5D6E, 2QCN, 6S9X, 3G3D, 6MHC, 3BLS, 1B5G, 4INH, 2OP3, 3MXS, 3VGC, 1RE1, 4XCU, 5UG8, 5VQZ, 2FM2, 6NVH, 2F9U, 3S3R, 1L6S, 2GBF, 4HCV, 4PL4, 3W2T, 6AFC, 4I7C, 6HUQ, 1G37, 6AFH, 2YJB, 5DP7, 5U4F, 4M8T, 5DP9, 4QVY, 3KRD, 4JMX, 2XU3, 4WBG, 4NO6, 2F9V, 2BDL, 6RN9, 2Y2J, 2YJC, 4QZ0, 4CCD, 1W14, 6N9P, 4QWG, 4PJI, 5GSO, 4HRD, 6MNY, 6G8N, 2V6N, 4MNX, 3BLU, 6HUC, 6G7F, 2XK1, 6OIM, 5WKM, 5FAS, 4E3M, 4D8I, 5TG1, 6HTC, 5V4Q, 4WMC, 6ALZ, 3HWN, 4MBF, 2HWO, 4QZ5, 1P05, 3MKF, 5C1X, 6CQT, 6B1O, 4YEC, 6QFT, 1EAT, 4HRC, 5WAE, 1IAU, 2ZU3, 6IYW, 3RJM, 1U9V, 1AUJ, 2QL7, 4HNP, 2Y2P, 6IC5, 3E90, 1YM1, 6FV2, 5W12, 1TYN, 6BL2, 4QKX, 3ZIM, 1QTN, 4AMY, 2NQI, 3OF8, 3EX3, 2H65, 5NE3, 1LHC, 1EKB, 1SNK, 5AHJ, 1MEM, 4KIO, 6ARY, 3OVX, 4INR, 5ZWE, 1NLJ, 1RHK, 4HCU, 2FXR, 4AMX, 6BIB, 4U0X, 6QW9, 1YK7, 2I03, 3LXS, 3SNC, 1ONH, 4QW1, 1TU6, 3B1U, 6B1W, 6HGY, 5UGC, 2Z3Z, 6MHD, 4MZ4, 4MNY, 5VND, 4JK5, 2QAF, 3OJ8, 6GCH, 1MWT, 6P8Y, 3KJQ, 4YRS, 6HUV, 2RCX, 2H5J, 5LVX, 4QWK, 5F02, 6D8E, 5WAF, 5EE8, 4QW3, 4NK3,

4JG6, 5G0Q, 2G63, 2Y55, 6B0V, 5MAE, 4R02, 5T6G, 5LCK, 6HUU, 5HG9, 6AFD, 6ERT, 4R6V, 4INU, 1A61, 6QG4, 5NGF, 4FZG, 5FAP, 4QQ5, 2WAP, 6GCR, 6BID, 5TYO, 4HBP, 2OBO, 6RTN, 5TG5, 4QZ7, 3EYD, 1PI5, 5MQY, 3SV7, 6HVT, 6GXY, 2FJ0, 4JJ8, 6RN7, 2XE4, 6QWB, 3VB4, 2A4G, 2Q9M, 4BSQ, 4AMW, 5ZA2, 3U1I, 3FKV, 6H0U, 4QW6, 3T9T, 1O41, 2FS8, 1E37, 3I4A, 6HV5, 4YQM, 4LV1, 6B1E, 2Y2H, 3BWK, 2YJ9, 1O4E, 5I23, 2OC7, 1AYU, 6FFN, 1QJ7, 5XYZ, 6AFF, 5TG7, 3BAR, 3I06, 5GWA, 5J5D, 4E3O, 6HVV, 4QZ6, 6PGO, 4WZ4, 2XZC, 1HBJ, 5HG7, 6G9S, 6AX1, 2LP8, 6GZY, 2YJ8, 4Q1S, 6AF9, 6QW7, 4L0L, 3SJO, 1AWF, 1F7B, 1TLO, 1QFS, 6IB0, 4FZC, 5NPB, 5NWZ, 6QWA, 1YMS, 4QZW, 2WZZ, 3IUT, 6SKB, 6J6M, 5C20, 1AWH, 2VGC, 1NKM, 6HV4, 6G8M, 6BQ0, 2R6N, 5HG5, 3N4C, 3K7F, 6PNM, 3SJI, 4IVK, 4QVQ, 4DMY, 6P8Z, 4RSP, 5CLS, 3O88, 2YJ2, 3V6S, 4X0U, 4AN0, 6HTP, 2XCN, 3SV6

Structures Containing Steric Clashes in PDBBind

Here are the PDBIDs for structures in PDBBind v2020 with minimum distance between heavy atoms in the ligand and the protein less than 1.75Å (count: 1303):

3N9S, 4U1B, 3ZHX, 1EW8, 2FZK, 2Q8Z, 5GOF, 1EX8, 1OLX, 3A6T, 1KM3, 4K55, 5EFA, 3K1J, 4L51, 3NXQ, 6D1H, 1FZQ, 1JLR, 6O5T, 1I9N, 4U6C, 2ZZ2, 1X8R, 4U71, 2RK8, 2V8W, 6HH3, 4FL2, 1RNM, 3ISS, 3DJV, 1PVN, 4U6W, 1B38, 1MAI, 1V48, 2C94, 1LCP, 3B7I, 1V11, 4EU0, 4R3W, 6CZB, 4NXV, 6CZC, 6D1G, 1NKI, 3B7R, 4K5P, 2V2H, 1W4Q, 1MRS, 1AFL, 2ISW, 4AXD, 3HU3, 3EVD, 1M83, 4DEL, 3RV4, 3IAE, 6D1B, 4UOH, 3B3C, 1WUQ, 3WGG, 4U0W, 3RBU, 3AHO, 6JDI, 1G98, 3A1E, 4K3N, 4QJX, 2OI2, 1JVU, 1B55, 2W8W, 6IHT, 2RCN, 2R75, 3D2E, 3PCG, 2GSU, 4NCN, 4KAX, 2C92, 1TLP, 4QRH, 2YAY, 1TKB, 3S8L, 1YQ7, 1G53, 1KSN, 4DCS, 1JYQ, 1QK4, 4P5D, 1FT7, 6D9S, 4PVY, 6OF5, 2QTN, 5D6J, 2AMT, 4L50, 4IDN, 6D1J, 1WUR, 1RNT, 1SLN, 1BXR, 3G1V, 1PKX, 4ZCS, 5CBM, 1LVU, 6JON, 3I73, 3EBI, 3EXH, 2TMN, 5HVA, 4U69, 3OVE, 2WF5, 4JNE, 4FL1, 2E91, 1M5W, 5FSX, 2XBP, 4U73, 5HWU, 2WE3, 6FAA, 6D15, 1RP7, 6D3Q, 5NW7, 1M7Y,

1YFZ, 1U0G, 2PY4, 4LPS, 1YDD, 5LTN, 3DJO, 3K5X, 2QTA, 3BXF, 5TMP, 2RIO, 1PFU, 2PU1, 1B57, 3A1C, 3D7K, 1S5Z, 1M0Q, 1A4R, 6J72, 3UPK, 4U70, 1V16, 6MNV, 3JUK, 4U6Z, 1ATR, 6FHK, 3OZG, 1OLS, 3EGT, 3AHN, 3OV1, 3AAS, 6DD0, 3Q71, 3EXE, 3FZN, 6D1A, 1D2E, 2E92, 3N1C, 1MUE, 5EVZ, 4ETZ, 4JYC, 6D18, 2PQC, 3C56, 6HH5, 5C2O, 3FZY, 1HYO, 5BV3, 2NSL, 6H77, 3I9G, 5UV2, 1HI4, 3DJP, 2R59, 4DY6, 1EW9, 2VT3, 2FXV, 5YHE, 5LDP, 1Q54, 2WZF, 3FUC, 2W5G, 2PTZ, 5F2R, 5HJQ, 1X8T, 2X97, 4OR6, 1VSO, 3MKE, 3HJ0, 2XLC, 6Q35, 4BCB, 5KHG, 3LOO, 3L0K, 5TIG, 6JN4, 2Y4M, 6EJI, 2PJ4, 4QIR, 3BG8, 1QF5, 6G15, 1EAS, 1Z6F, 1BIO, 6B1H, 3BJM, 5I24, 3ZCZ, 5YBI, 3HU2, 6N4T, 2I72, 4QVM, 1KDW, 3POA, 1TMM, 4EFG, 6HVS, 3GGC, 2VF6, 4R3B, 1SRE, 1P06, 5O3R, 5TG4, 5JNL, 1NU8, 2OC1, 3ATV, 1P04, 5FAO, 5IZM, 4QWU, 2CFG, 6N97, 5EOU, 4WKS, 4OCP, 5C1Y, 3UIG, 2Y1G, 4NO8, 1ONP, 2G5T, 1KE3, 4QWL, 3ZMH, 5T66, 3KR5, 5D9P, 1DB4, 1WC6, 3D4F, 4F3H, 1MXO, 1L6Y, 1Y8P, 1LHE, 2PJA, 1PI4, 4ZQT, 4BCC, 3LJ7, 6CZD, 5JEK, 5WAG, 3TDH, 6DA4, 3UED, 3WNS, 5OM9, 6B1J, 3EWZ, 1O45, 3D9P, 5OYD, 1QWU, 1H79, 4DXJ, 4OON, 1KDS, 6B1F, 4UMJ, 4QWS, 1TKC, 6BKX, 5H6V, 1ONG, 5ZDG, 4KXM, 6I5J, 4P4F, 4QVL, 5X02, 5J7S, 5J7J, 4AN1, 4DN0, 4P4S, 5FAT, 1T29, 1P10, 1R0X, 2H5D, 5B5P, 1QJB, 4WKV, 4AMZ, 5FA7, 6FEL, 6RNE, 1Y8O, 4QZ2, 1QCP, 3GQO, 2MC1, 3ZP9, 2AUX, 2PJC, 1I8J, 3E0P, 1K2I, 6G01, 2FDA, 1O4K, 6AEC, 5W38, 3QXC, 6B22, 6B0Y, 1VSN, 6FA5, 4YX9, 5TYN, 4KP4, 6NNG, 5CYI, 4BXN, 4G8L, 3PA8, 6FAC, 6IPL, 6M9F, 4K5N, 4LEN, 1AO0, 4OOL, 4MNV, 4E1E, 1ZPC, 1KWR, 6B1Y, 3FYZ, 5L44, 4YB7, 6R4V, 1CSI, 1H7A, 2Z7H, 1OTH, 4KQO, 5F1C, 4JJF, 4EEJ, 1AHT, 2FS9, 6MKQ, 4GK7, 3NZI, 1QJ6, 4E3L, 2J4K, 4J70, 6HVU, 1P01, 5INH, 1H07, 3S1Y, 4QW0, 5W14, 5DP6, 5HL9, 4WYZ, 5ACB, 1TPZ, 4O7D, 3X1K, 2WIJ, 4S2I, 5ICV, 5KRE, 4R17, 3TJM, 2JBV, 5TYL, 4QLU, 2QPJ, 3TL0, 2G5P, 2WJ2, 2ZZ6, 4QVV, 2XNI, 2QNZ, 6AGP, 4PKB, 6HVW, 1LF8, 1RTL, 3BH3, 4QW5, 1EBG, 1NY0, 1E34, 1U9W, 2IOA, 1YMX, 4K8O, 6SKD, 2Z7I, 2WJ1, 1TL7, 1X07, 2Y1D, 9LPR, 1GA9, 1MN9, 3WDC, 6AK6, 4QLV, 6R4S, 6FMP, 3RDH, 3DPC, 5FOO, 2WOQ, 1FSW, 2PJ5, 6HV7, 5AB0, 3RYW, 6UN3, 1QJ1, 5TTU, 3L0N, 6AEH, 3MXR, 6Q5B, 1CSS, 6CZU, 4R18, 1G05,

5JH6, 6OM4, 4JG0, 4WYY, 4IMZ, 5EEC, 6EYZ, 3HU1, 2VHQ, 5NPF, 2Y59, 4E3I, 6PK7, 5UV1, 5Z68, 4K5M, 6IPM, 4QZ3, 2XDM, 4LV3, 2C1N, 3AAV, 4FUT, 4FR3, 3ZMI, 1PPW, 8LPR, 5BRN, 1NZV, 4QWX, 5F29, 4WKU, 3EXF, 6N54, 4GKC, 4RAB, 3MBZ, 4PL5, 4JJG, 2XAQ, 2P0X, 4XJR, 6IPH, 2LPR, 6FDU, 4U6E, 5HLB, 3HWX, 1UOD, 4DEM, 1P6D, 1AKQ, 1LV8, 1AMN, 6QG7, 4J5P, 6FDQ, 5NPR, 3FHE, 3LPR, 5FAQ, 1MPL, 5L6H, 3VB5, 1FSY, 4X68, 3V4J, 4QLQ, 6HVR, 1O8B, 6NZG, 4HWT, 6DB4, 1ML1, 1AKV, 5JAZ, 1ORW, 2OZ2, 3EX6, 5WFJ, 6M8Y, 4X6J, 1B39, 1NQC, 5UQ9, 4EST, 5W10, 4JN4, 6D3G, 1EIX, 1RYH, 6N94, 4QW4, 2A4R, 5YR5, 1KF0, 1D7X, 2QTR, 5EY8, 5JMP, 5J9Z, 2Y2N, 4YV8, 1INC, 3T2C, 3LXO, 6MLF, 1E8H, 5NE1, 3PR0, 1JBD, 3FMQ, 4OB2, 3FMR, 2WZX, 1O4I, 6RNU, 6S1S, 5U4G, 1MY8, 1YHM, 2C9D, 3D67, 5YR6, 1ZPB, 4WKT, 6UN1, 6QW8, 4X69, 3ZEB, 6IB2, 6HUB, 7LPR, 3ZKF, 4UV9, 5K6S, 2Z4W, 6B1X, 1NYY, 5IZL, 4WSK, 3VB7, 3K41, 3O87, 5BSK, 4QVN, 3L6F, 3CST, 5OD5, 3K84, 1VJC, 2Y2I, 5VQE, 3ZVW, 2A4Q, 4WUP, 6HTD, 5F5B, 1C7F, 4LOJ, 4WZ5, 3SHV, 1YLV, 2QM7, 1YON, 1VJD, 1U2R, 3ZY2, 3E16, 6Q30, 2Y4L, 1IEM, 1AD8, 6EUV, 5W13, 2QCD, 4I9S, 4GA3, 5AGJ, 5J8X, 4L2X, 4BG6, 5NUD, 3TDZ, 5KHD, 3AU6, 1GGN, 4E3N, 5X5G, 4EHM, 3GZN, 5NHZ, 3BM6, 4QWI, 1H8Y, 2WQP, 2WGI, 6J7L, 1RU2, 4NZM, 1TBZ, 3AXK, 5YKP, 4LIL, 4HE9, 1RGK, 1GGD, 5NZ2, 2WIK, 6RMM, 4QVW, 6LPR, 4WM9, 4QVP, 5GSW, 2PU0, 1ZOM, 6PXC, 5FB0, 1Y19, 3LOX, 1T3T, 4CLP, 2XDW, 1YLY, 6M8W, 5XHR, 4YAS, 1O4D, 1I9L, 3EWU, 2QKY, 2ORK, 5KYK, 6ISD, 6HVA, 6DD1, 1P02, 1ERO, 6GFM, 1AKU, 1W31, 3OPR, 1O43, 4RHU, 2PJ0, 6HTR, 6AR2, 3MLE, 4VGC, 6HYS, 3OPP, 3LLM, 4U0G, 5WAD, 1I9Q, 4EXZ, 4Y67, 4GD6, 4H38, 4NO1, 5LC0, 6E5S, 4FL3, 6AK5, 4WSJ, 1U9X, 4QZ1, 5NYZ, 3WDZ, 3ZOT, 3ZMQ, 6K1S, 2XLN, 2XOW, 3WNR, 6G9F, 3V7D, 3KC0, 3VB6, 4QWR, 3NCQ, 4MVN, 5LPR, 4A0J, 3RG2, 3Q7P, 1QJA, 3FNM, 3IQV, 3IUC, 1MNS, 3RBM, 4OAZ, 2AUZ, 6IA7, 4G5Y, 6G2N, 1B0F, 2AJD, 3D62, 2Q9N, 1C3B, 6GIU, 5TYJ, 6RJP, 1BWN, 2Q3Z, 1I9M, 1NJT, 5J9Y, 4U0U, 2L0I, 4KW6, 4QXJ, 1NZL, 2Y8L, 1P03, 4ZCW, 3EX2, 3QKV, 3BLT, 4UVA, 3ZMJ, 3NBA, 2JAL, 4NNN, 5NPS, 1QHR, 6C5J, 4Q2K, 4W5J, 4EDE, 4LQY, 4QQC, 2E95, 4GS9, 4I9R, 6M9C, 4P5E, 3SV8, 1W12, 2V0C, 2HQU, 4OEL, 6AK4,

2E98, 1O4F, 5J7P, 2Y2K, 3QT7, 2O1V, 4NNW, 3ZS1, 3ATU, 3ZVT, 2QLQ, 1RU1, 6M9D, 5ZDC, 5TYK, 6B4H, 6OVZ, 2HA0, 4QWJ, 3GPJ, 2Y4A, 3OB2, 2H5A, 2AJL, 1BJR, 3Q72, 4LV2, 2CMC, 2GVF, 5HH4, 4UUQ, 2GBG, 5W8V, 4WX7, 2ZA3, 5ZWF, 4XUZ, 1AKR, 5HLD, 3CF1, 1MF4, 5D6F, 4WN0, 2PJ6, 1IEW, 6BS5, 2HOB, 4DWG, 5YOF, 5TG6, 1VGC, 5DOH, 3O86, 1X6U, 4QZ4, 3G3M, 6CQZ, 1O4A, 5FQ9, 5EST, 4PRY, 5A3H, 1DOJ, 4E3J, 3K83, 4AY6, 1GVK, 6IPJ, 3S22, 5TEH, 3WYJ, 1GBT, 6PGX, 4W4S, 1ERQ, 2E93, 3G4F, 4N1Z, 1A09, 4PL3, 2WIG, 3EHW, 1LHD, 1AZL, 4QW7, 7GCH, 5MXQ, 2Z52, 2O4H, 3BH8, 4QZX, 1W10, 4NO9, 5D11, 1LLB, 5TYP, 5IM3, 1CSH, 6E5B, 4E3K, 1LHG, 2A29, 1NFS, 1Y98, 3U8D, 1LHF, 1RXP, 4M1J, 6GOP, 5HYX, 4QWF, 5YR4, 1H1B, 6QXS, 3A1S, 4PID, 4GV8, 1NJU, 2QCG, 1KE0, 5WAC, 2OC0, 6IPI, 6EGS, 3Q2G, 2AJB, 5W19, 5JK3, 1NO9, 5MOC, 6E7M, 5MI3, 5SVK, 5X79, 4INT, 4RXC, 3FCK, 1LRT, 5H63, 5D6E, 2QCN, 3G3D, 6AI9, 3V0P, 3BLS, 1H9L, 2OP3, 3MXS, 3VGC, 6NPF, 5L6J, 2FM2, 2F9U, 1VKJ, 2Q80, 4TMF, 1L6S, 5EWZ, 2GBF, 4PL4, 1RYF, 3W2T, 1XFV, 6HUQ, 5WRS, 1G37, 1CSR, 4JBS, 1MAU, 3QX8, 5DP7, 4M8T, 4QVY, 4K5O, 3KRD, 4JMX, 5AEL, 4RAQ, 4WBG, 6N9T, 5OLK, 2PJ2, 2K0X, 4NO6, 1FPY, 2F9V, 2BDL, 6RN9, 4K19, 2Y2J, 4QZ0, 3E4A, 4CCD, 1W14, 6N9P, 6RML, 4U82, 4QWG, 4PJI, 5GSO, 1GX4, 5M9D, 4HRD, 6G8N, 3BB1, 4QLS, 2E99, 3BLU, 6HUC, 6G7F, 3IOI, 2XK1, 5EY9, 4GZ3, 3UX0, 3UO9, 5FAS, 4E3M, 6HTC, 5V4Q, 4WMC, 3PJT, 4MBF, 4QZ5, 1P05, 3MKF, 5C1X, 6CQT, 1NYM, 6B1O, 6QFT, 1GX0, 3CPH, 5HH6, 1EAT, 1U0H, 4HRC, 4EUV, 5WAE, 1IAU, 2H1H, 1OXG, 3RJM, 1AUJ, 5EXX, 4HNP, 2Y2P, 6IC5, 3E90, 6H78, 3D9L, 1YM1, 5G1P, 4RAC, 5W12, 1TYN, 6BL2, 2ITK, 2G83, 3FV7, 3ZIM, 1QTN, 4AMY, 5HH5, 3EX3, 5NE3, 1LHC, 5A3R, 1EKB, 5AHJ, 5E2V, 3BUO, 6ARY, 6MMC, 4INR, 2FXR, 4AMX, 4U0X, 6QW9, 2P59, 2I03, 1BSK, 3D9M, 1ONH, 4QW1, 4K5L, 1I3Z, 6B1W, 2QCF, 3BU6, 2P1C, 1FT4, 5VND, 4YM4, 2QAF, 4DWB, 2PLL, 3RSB, 3LDW, 16PK, 3OJ8, 6GCH, 1MWT, 2QCM, 4V11, 6HUV, 2RCX, 5LVX, 4QWK, 2JG8, 5WAF, 5EE8, 4QW3, 4NK3, 1ELS, 2IT4, 5G0Q, 2WVA, 3B9S, 2G63, 2Y55, 6B0V, 5MAE, 4R02, 6IPK, 6HUU, 1YYY, 6ERT, 4R6V, 4INU, 1G9R, 5OSY, 4FZG, 2JBJ, 5FAP, 4QQ5, 2WAP, 5TYO, 5X9H, 4HBP, 2OBO, 6RTN, 5TG5, 1I9O, 4QZ7, 3EYD,

1PI5, 3SV7, 6HVT, 2FJ0, 5E2N, 2XE4, 6QWB, 4KXL, 3VB4, 2A4G, 2Q9M, 4BSQ, 5ZA2, 2WIC, 6G6X, 3U1I, 4JFX, 5KNU, 3FKV, 5JBI, 4QW6, 1O41, 2FS8, 1E37, 6CSE, 5LVF, 4OSF, 6HV5, 4LV1, 6B1E, 2Y2H, 3KSL, 1O4E, 5I23, 2OC7, 4RUU, 1QJ7, 5XYZ, 5KNR, 5K7H, 1SZ0, 6BUU, 5TG7, 2AD5, 3BAR, 4AY5, 5GWA, 4E3O, 6NNR, 6HVV, 3FZC, 4QZ6, 1M9N, 4WZ4, 2XZC, 1HBJ, 6G9S, 6AX1, 5EJE, 1TKA, 6GZY, 2L75, 3FSJ, 4OEM, 4Q1S, 3NCR, 5L6I, 6MU1, 6QW7, 4L0L, 5NI0, 4QLT, 1AWF, 1F7B, 6AEJ, 1QFS, 6IB0, 4FZC, 6OTT, 5NPB, 6QWA, 2F89, 1BA8, 2PSX, 4RAO, 3ZJC, 1YMS, 4QZW, 2WZZ, 2Y8Q, 6CJJ, 4GE5, 6SKB, 1AWH, 3HXE, 5JC1, 2VGC, 1SYO, 1NKM, 6HV4, 6G8M, 6BQ0, 3UEC, 5ZDE, 1JRS, 3K7F, 4IVK, 3WNT, 3BGM, 4QVQ, 2HR6, 5CLS, 3O88, 4P4T, 4X0U, 6E3P, 2ONB, 4AN0, 6HTP, 2XCN, 3SV6

Supporting Tables

Table 1: Counts and proportion of chemical element occurrences in all proteins in the PDB-Bind dataset

**Element**	**Number of data containing element**	**Proportion of data containing element**
C	19398	1.00
S	16375	0.84
O	19398	1.00
N	19398	1.00
Zn	1842	0.09
Ca	400	0.02
Co	66	0.003
Mg	858	0.04
Ni	79	0.004
Mn	356	0.02
Fe	104	0.01
Na	138	0.01
K	78	0.004
Se	107	0.01
Hg	6	0.000
Cu	16	0.001
Cs	1	0.000
P	105	0.01
Cd	22	0.001
Sr	1	0.000
Ga	1	0.000
Rb	1	0.000

Table 2: Counts and proportion of chemical element occurrences in all ligands in the PDB-Bind dataset. Only elements that occur in the binding pocket were considered.

**Element**	**Number of data containing element**	**Proportion of data containing element**
C	19398	1.00
S	5146	0.27
O	18338	0.95
N	17515	0.90
Cl	2255	0.12
P	2087	0.11
F	2500	0.13
Br	459	0.02
I	133	0.01
B	240	0.01
Se	16	0.001
Fe	21	0.001
Co	4	0.000
V	2	0.000
Ru	10	0.000
Be	1	0.000
Ir	5	0.000
Zn	1	0.000
Mg	1	0.000
Re	1	0.000
As	4	0.000
H	6	0.000
Cu	1	0.000
Pt	2	0.000
Rh	1	0.000
Si	5	0.000
Sb	1	0.000

Table 3: Retrained AutoDock Vina Weights

**Term**	**Weight**
gauss1	0.003372
gauss2	-0.008098
repulsion	0.014212
hydrophobic	-0.008361
hydrogen	-0.227928
rot	0.05846

Supporting Figures

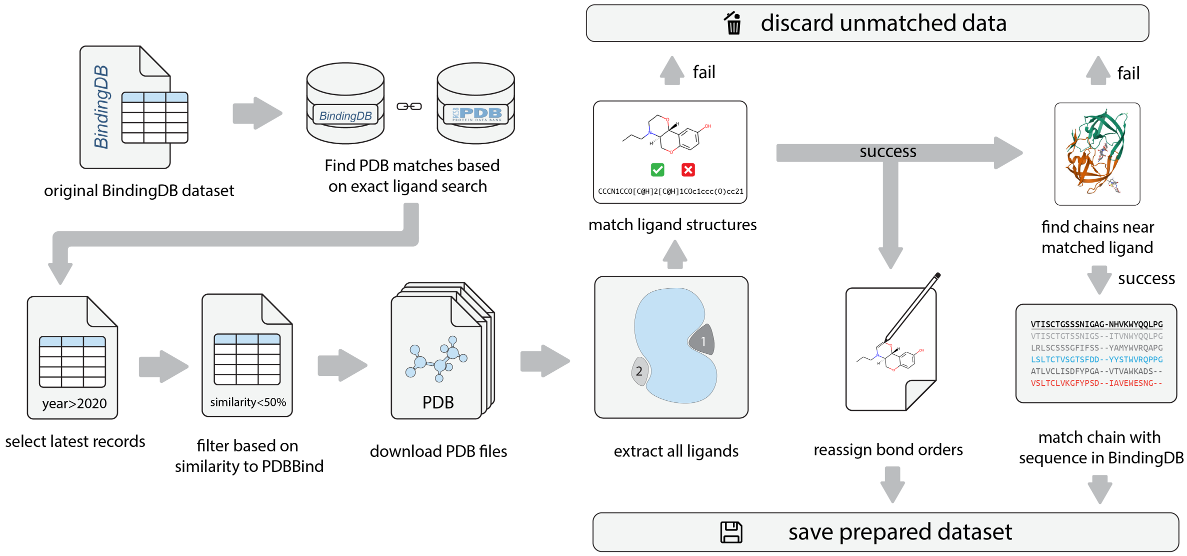

Figure 1: *Flowchart of the BDB2020+ curation process.* The original BindingDB dataset[?, ?] was matched with PDB database records by exact ligand matches. The matched records were further filtered based on release date and similarity with the PDBBind dataset. All ligands were extracted from the downloaded PDB files, and the ligand matching the SMILES in the BindingDB dataset was selected for further process. Protein chains in the vicinity of the ligand were also extracted. Both the matched ligand and protein chains were further processed to ensure identity with the record in PDBBind. Any discrepancy will result in a mismatch of data and will be discarded.

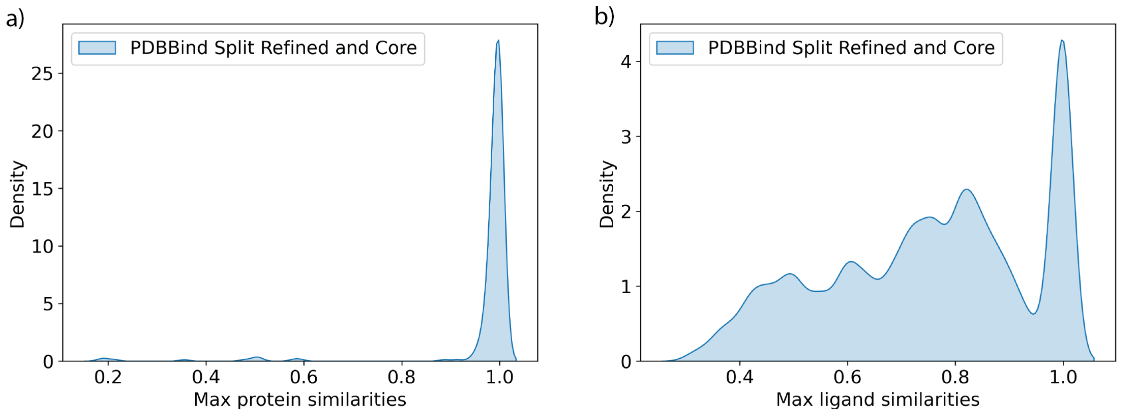

Figure 2: *Data similarity between refined set and core set in the PDBBind original split.* Comparison of maximum protein similarities (a) and ligand similarities (b) between refined set and core set under original split of PDBBind.

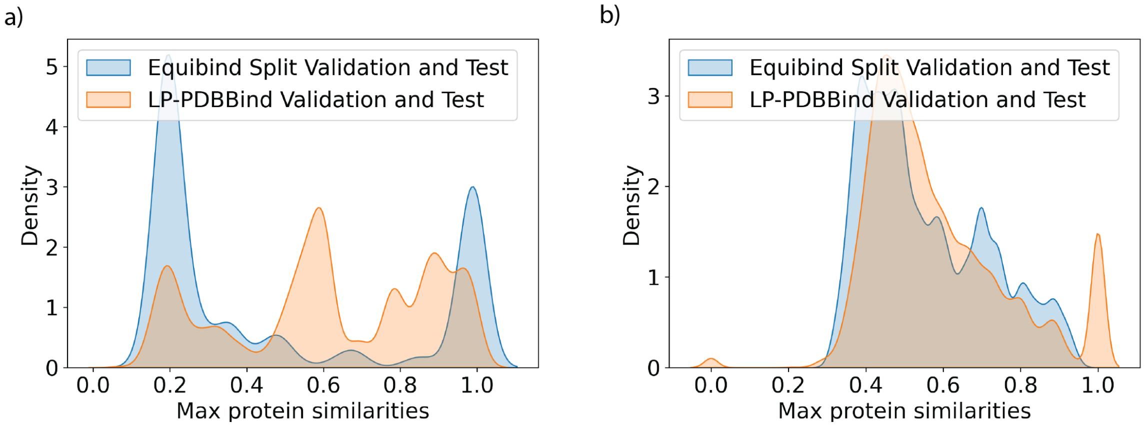

Figure 3: *Data similarity between validation set and test set in different splittings of PDB-Bind.* Comparison of maximum protein similarities (a) and ligand similarities (b) between validation set and test set under Equibind split and UCBSplit of PDBBind.

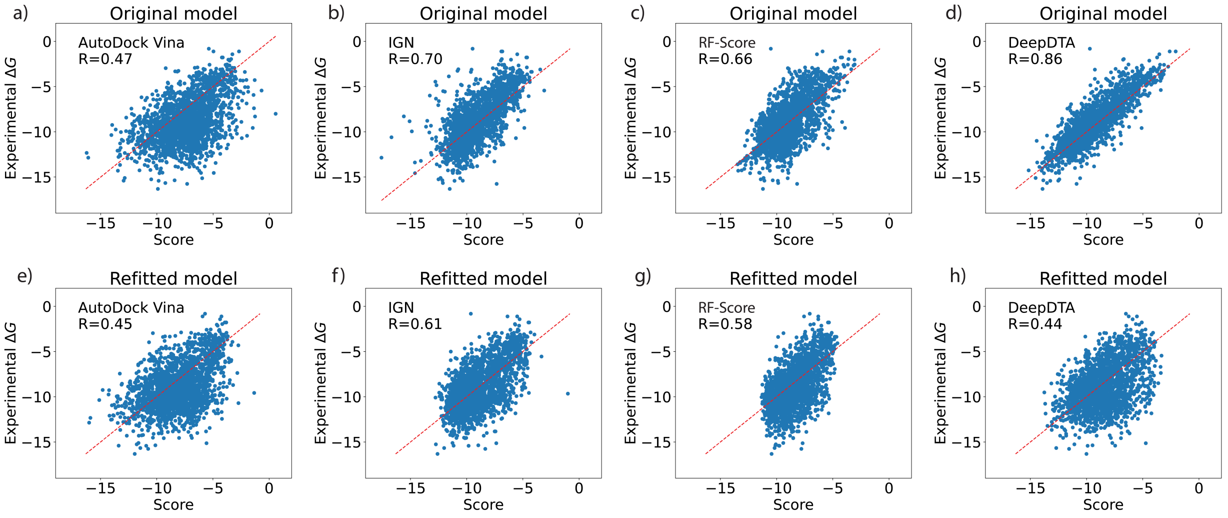

Figure 4: *Scatter plots for predicted scores and experimental binding affinities before and after model retraining for different models evaluated on UCBSplit test dataset.* Scatter plots for original models: (a) AutoDock vina, (b) IGN, (c) RF-Score, and (d) DeepDTA; Scatter plots for models retrained with UCBSplit: (e) AutoDock vina, (f) IGN, (g) RF-Score, and (h) DeepDTA.

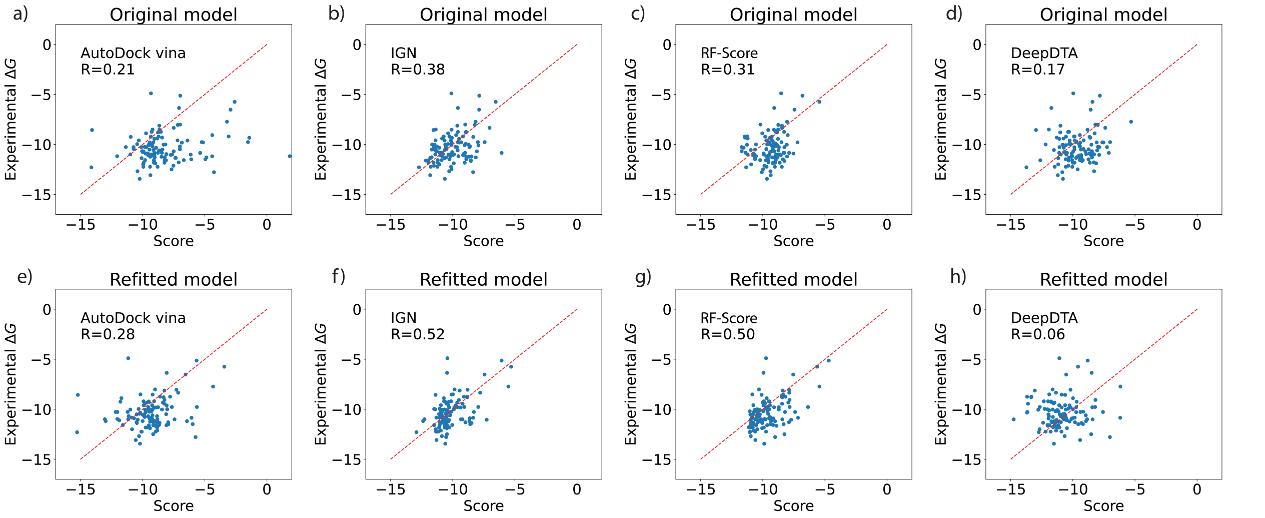

Figure 5: *Scatter plots for predicted scores and experimental binding affinities before and after model retraining for different models evaluated on BDB2020+ benchmark dataset.* Scatter plots for original models: AutoDock vina (a), IGN (b), RF-Score (c) and DeepDTA(d); Scatter plots for models retrained with UCBSplit: AutoDock vina (e), IGN (f), RF-Score (g) and DeepDTA (h)

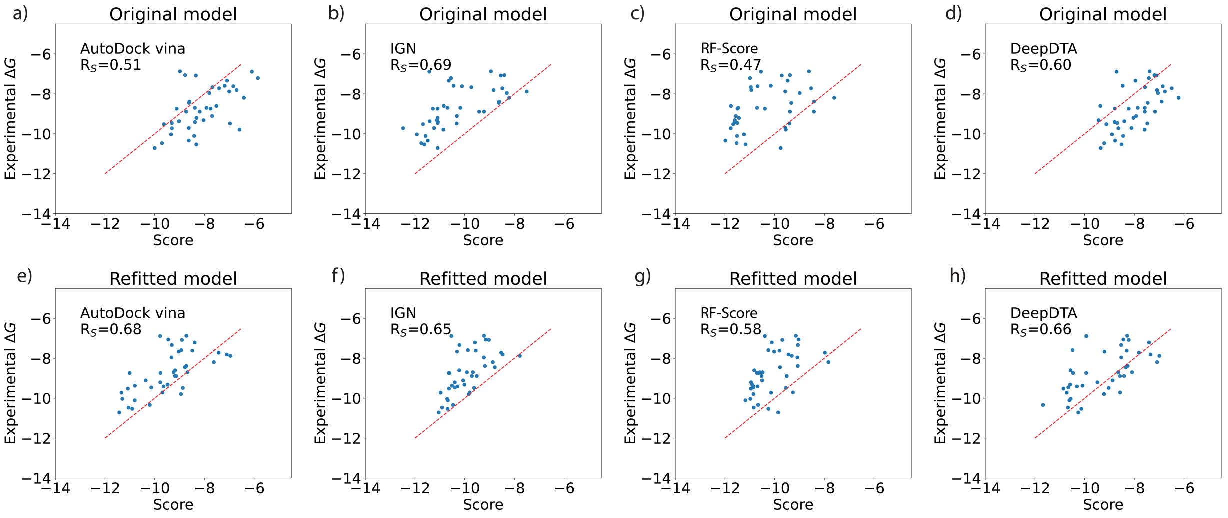

Figure 6: *Scatter plots for predicted scores and experimental binding affinities before and after model retraining for different models evaluated on SARS-CoV-2 main protease (Mpro) benchmark dataset.* Scatter plots for original models: AutoDock vina (a), IGN (b), RF-Score (c) and DeepDTA(d); Scatter plots for models retrained with UCBSplit: AutoDock vina (e), IGN (f), RF-Score (g) and DeepDTA (h)

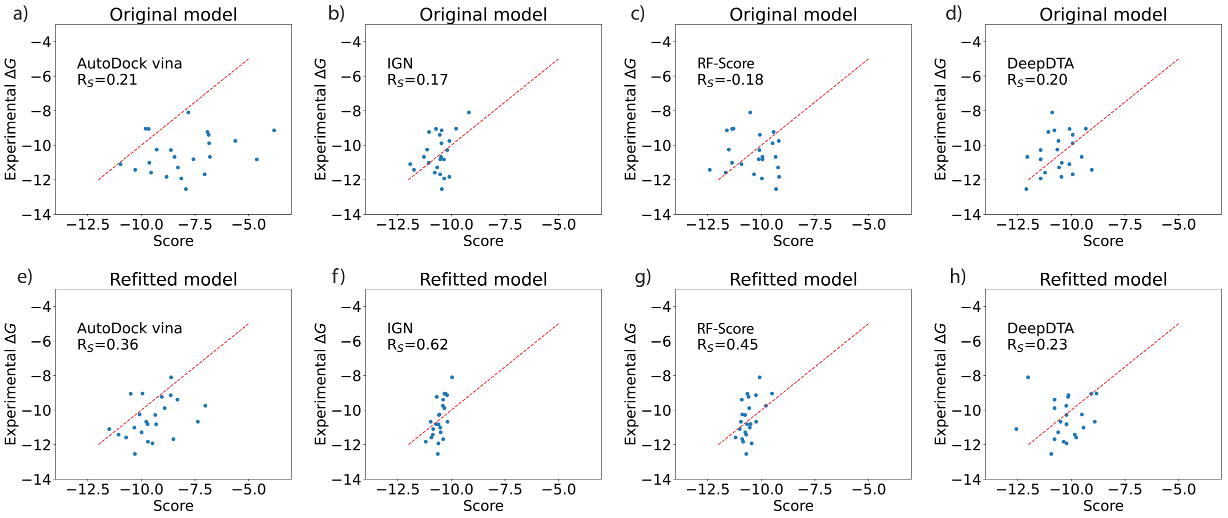

Figure 7: *Scatter plots for predicted scores and experimental binding affinities before and after model retraining for different models evaluated on epidermal growth factor receptor (EGFR) benchmark dataset.* Scatter plots for original models: AutoDock vina (a), IGN (b), RF-Score (c) and DeepDTA(d); Scatter plots for models retrained with UCBSplit: AutoDock vina (e), IGN (f), RF-Score (g) and DeepDTA (h)
